# Skin-derived G-CSF activates pathological granulopoiesis upon psoriasis

**DOI:** 10.1038/s44321-026-00456-y

**Published:** 2026-06-16

**Authors:** Tomson Kosasih, Tatsuya Morishima, Sohyeon Lee, Jungyeon Yoon, Kanako Wakahashi, Pilhan Kim, Aiko Sada, Hitoshi Takizawa

**Affiliations:** 1 Laboratory of Stem Cell Stress, International Research Center for Medical Sciences (IRCMS), Kumamoto University, Kumamoto, 860-811 Japan; 2 Laboratory of Epitranscriptome in Hematopoiesis, International Research Center for Medical Sciences (IRCMS), Kumamoto University, Kumamoto, 860-811 Japan; 3 IVIM Technology Inc., Daejeon, 34013 Republic of Korea; 4 Graduate School of Medical Science and Engineering, Korea Advanced Institute of Science and Technology (KAIST), Daejeon, 34141 Republic of Korea; 5 Division of Skin Regeneration and Aging, Medical Institute of Bioregulation (MIB), Kyushu University, Fukuoka, 812-8582 Japan; 6 Center for Metabolic Regulation of Healthy Aging (CMHA), Kumamoto University, Kumamoto, 860-811 Japan

**Keywords:** Immunology, Skin

## Abstract

Psoriasis is an inflammatory skin disease initiated by environmental triggers and driven by disruption of T cell cytokine network in the cutaneous milieu. The fact that complete resolution of disease by targeting the key inflammatory cytokines remains challenging indicates a contribution of other immune cells to the pathogenesis. Here, we study the role of neutrophils in psoriasis, the first-line innate immune defender that is short-lived but mobile and infiltrative into various tissues. We found that upon psoriasis induction, skin-resident endothelial cells are activated to produce G-CSF which contributes to emergency granulopoiesis in bone marrow and cutaneous accumulation of inflammatory neutrophils. Depletion of neutrophils or blockage of psoriasis-driven granulopoiesis by respective neutralizing antibodies lead to the reduction of cutaneous neutrophil burden and mitigates psoriasis pathogenesis. This mechanism appears to be conserved in human psoriasis, confirmed by public RNA-seq database reanalysis. Our findings uncovered and detailed the pathological crosstalk between skin and BM in psoriatic inflammation, proposing a potential therapeutic approach targeting cross-organ communication.

## Synopsis

A G-CSF-mediated skin-bone marrow axis that contributes to psoriasis pathogenesis was characterized both in mouse and human.

- Excess neutrophil number in psoriatic skin exacerbates inflammation.
- Skin endothelial cells are a key source of G-CSF in mouse and human psoriasis.
- Skin-derived G-CSF drives pathogenic granulopoiesis in psoriasis.
- G-CSF inhibition attenuates psoriasis symptoms.

## Abstract (web summary)

A G-CSF-mediated skin-bone marrow axis that contributes to psoriasis pathogenesis was characterized both in mouse and human.

## Graphical abstract

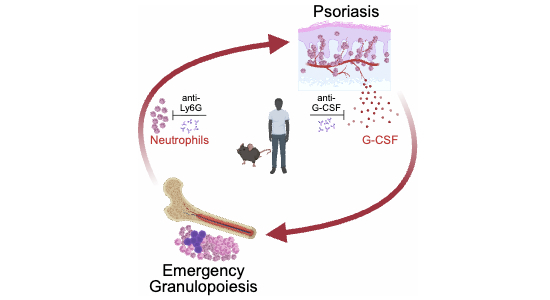

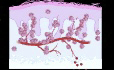

The paper explained **Problem**

Complete remission from psoriasis remains difficult to achieve. Although neutrophils are potent drivers of inflammation and their accumulation in skin is a hallmark of the disease, current therapeutic strategies primarily focus on T cells, often leaving neutrophil function understudied. This oversight stems from a long-standing uncertainty: is the excess of neutrophils in the skin a primary cause of the disease or merely a secondary consequence? If neutrophils are indeed pathogenic, identifying a viable therapeutic strategy to target them becomes essential.

**Results**

This study establishes that neutrophils accumulating in psoriatic skin are pathogenic effectors for psoriasis. In psoriatic skin, they exacerbate inflammation by increasing superoxide production and IL-17A expression. We demonstrated a systemic feedback loop: inflamed skin release G-CSF, which signals the bone marrow to trigger “emergency granulopoiesis,” fueling a continuous oversupply of neutrophils. Blocking G-CSF or depletion of neutrophils reduces skin-infiltrating neutrophils, and significantly mitigates disease severity. Analysis of human data confirms that this endothelial cell-derived G-CSF signal is conserved in patients.

**Impact**

Our findings have made a conceptual advancement to redefine psoriasis pathogenesis by highlighting the underappreciated role of neutrophils and their production via skin-bone marrow crosstalk. Clinically, targeting the G-CSF/neutrophil axis offers a promising translational strategy. This approach could complement existing T-cell-targeted biologics, potentially overcoming therapeutic resistance and providing a more comprehensive treatment for patients with psoriasis.

## Introduction

Psoriasis is an inflammatory skin disease which affects more than 60 million patients worldwide, and involves environmental triggers such as infections, air pollutants, and excessive sunlight exposure, progressively abolishing tissue integrity and aggravate the pathologies overtime (Griffiths et al, 2021). At its core, psoriasis is an immunological disorder driven by disruption of cytokine network, particularly IL-17 overtone caused by hyperactive T-helper 17 (Th17) in the cutaneous milieu. Although Th17-based immunopharmacological rewiring mitigates the disease, complete resolution remains largely unachievable (Mease et al, 2000; Leonardi et al, 2012; Gordon et al, 2018; Krulig and Gordon, 2010), indicating the likely contribution of other immune cell subsets.

A hallmark of psoriasis is the formation of microabscesses caused by neutrophil infiltration (Schön et al, 2017). Neutrophils are the first-line innate immune defenders which respond quickly and effectively to broad spectrum of antigens (Iwasaki and Medzhitov, 2015). Upon systemic infection, granulopoiesis is rapidly activated in the bone marrow (BM), shifting hematopoiesis toward neutrophil generation, often referred to as emergency granulopoiesis, in order to clear pathogen (Manz and Boettcher, 2014). However, little did it known whether local inflammation in peripheral organ can elicit hematopoietic response in distal BM, and if so, what is the underlying mechanism to mediate cross-organ communications.

Tissue and circulating neutrophils are naturally short-lived and constantly replenished through de novo granulopoiesis in the BM. IL-1, IL-3, IL-6, granulocyte-macrophage colony-stimulating factor (GM-CSF), and granulocyte-colony stimulating factor (G-CSF) are key cytokines that differentiate hematopoietic stem progenitor cells (HSPCs) into granulocyte lineage and determines cell pool in a systemic manner. Especially, G-CSF is shown to be secreted by BM-resident endothelial cells (ECs) upon its Toll-like receptors (TLRs) sensing and drive emergency granulopoiesis (Boettcher et al, 2014; Lieschke et al, 1994; Yvan-Charvet and Ng, 2019). Given the excess accumulation of short-living neutrophils in the skin upon psoriasis, we hypothesized that skin would initiate long-distance communication to the BM possibly through a blood-circulating signal, and activate emergency granulopoiesis program to meet the local demand in inflamed skin.

Here, using an inducible psoriasis mouse model (van der Fits et al, 2009), we demonstrated that psoriatic skin inflammation induced cutaneous neutrophil infiltration and skin-derived G-CSF production, with skin-resident ECs representing a notable contributing source. This systemic G-CSF response, in turn, activates emergency granulopoiesis in the BM. Transcriptomic analysis reveals that skin-infiltrating neutrophils were functionally overactive. Psoriasis-driven emergency granulopoiesis was lessen by anti-GCSF neutralization resulting in the lowered cutaneous neutrophil burden leading to disease mitigation. This mechanism reflects human psoriasis setting as RNA-seq public data reanalysis reveals the analogous phenomenon. Our findings uncovered and detailed the pathological crosstalk of skin-BM axis in psoriatic inflammation.

## Results

### Psoriasis induces progressive infiltration of neutrophils into the skin

**Figure 1:**
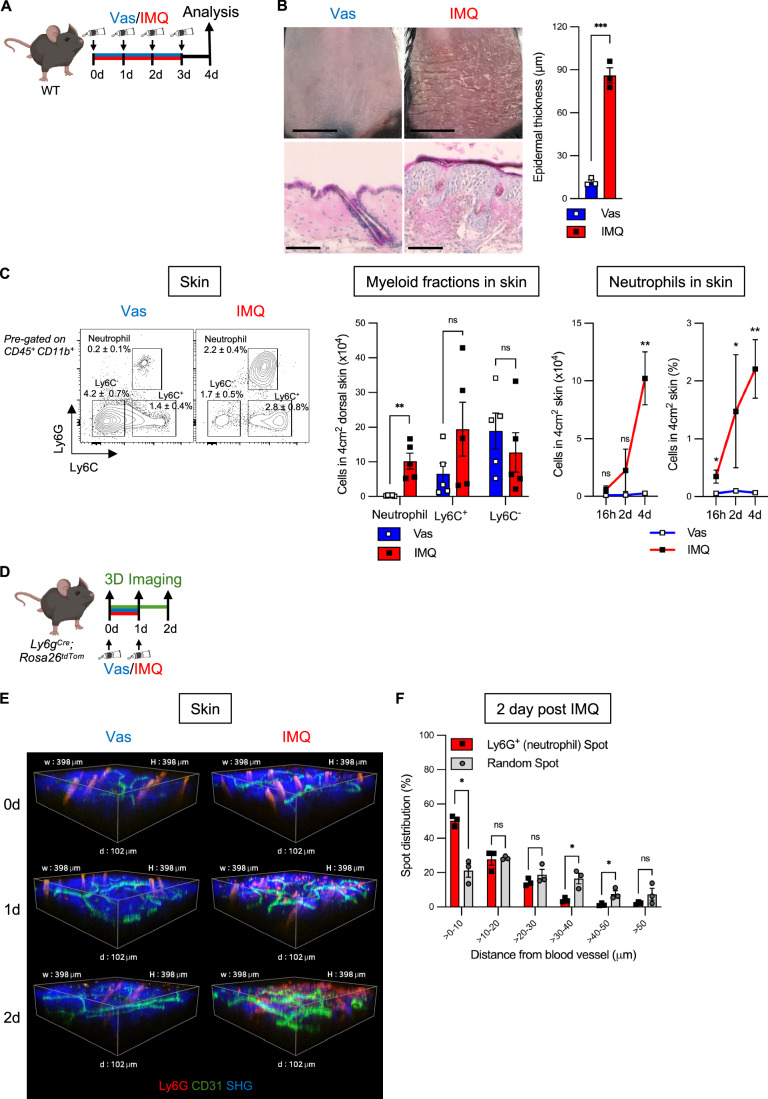
Psoriasis induces neutrophil migration and infiltration to the inflamed skin. (**A**) Experimental scheme of IMQ-induced psoriasis mouse model for results depicted in (B and C) (Vas, vaseline cream; IMQ, Imiquimod cream). (**B**) Clinical manifestation of psoriasis at 4 d. Representative skin images (upper panel, shaved dorsal skin with 1 cm scale bar; lower panel: HE histology with 100 µm scale bar). Bar graph shows epidermal thickness (µm) in mice treated with Vas (*n* = 3) or IMQ (*n* = 3). *t*-test: *p* = 0.0002. (**C**) FACS analysis of myeloid cells in skin. Representative FACS plot pregated on CD45^+^CD11b^+^ cells. Number of myeloid cells (neutrophil, Ly6C^+^ and Ly6C^-^ cells) at 4 d after treatment with Vas (*n* = 3–5) or IMQ (*n* = 3–5). *t*-test: *p* = 0.0025 (neutrophil), *p* = 0.1606 (Ly6C^+^), *p* = 0.4388 (Ly6C^-^). Time-course kinetics of skin neutrophils at 16 h, 2 d, and 4 d following topical application with Vas (*n* = 3–5) or IMQ (*n* = 3–5). *t*-test/Mann–Whitney test: *p* = 0.1199 (16 h), *p* = 0.2429 (2 d), *p* = 0.0025 (4 d) for cell number and *p* = 0.0341 (16 h), *p* = 0.0500 (2 d), *p* = 0.0014 (4 d) for percentage. (**D**) Experimental scheme of intravital microscopic skin imaging for results depicted in (**E**, **F**). (**E**) Representative time-course images of neutrophils (Ly6G^+^), blood vessel (CD31^+^), and collagen (SHG) at 0 d, 1 d, and 2 d post Vas/IMQ treatment. (**F**) Distribution of neutrophils localized from nearest blood vessel measured from 2 d post IMQ compared to random spots (*n* = 3). *t*-test: *p* = 0.0025 (> 0–10), *p* = 0.8548 (> 10–20), *p* = 0.2638 (> 20–30), *p* = 0.0166 (> 30–40), *p* = 0.0299 (> 40–50), *p* = 0.2129 (> 50). Data are pooled from ≥2 independent experiments. Each dot shown in the chart represents the measurement from 1 experimental subject and shown as mean±S.E. *p < 0.05; **p < 0.01; ***p < 0.001. Source data are available online for this figure.

**Figure EV1:**
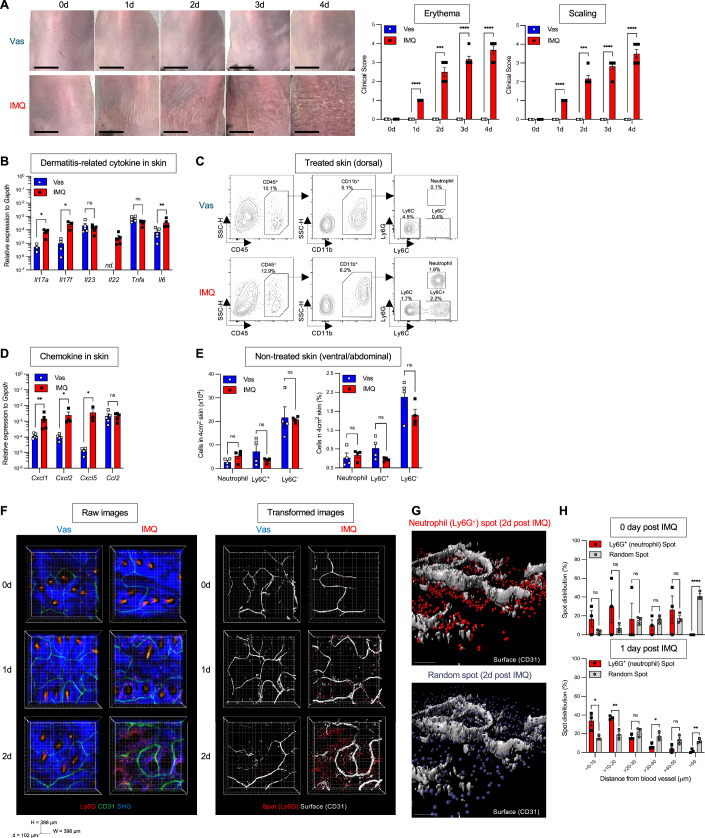
Characterization of clinical manifestations, myeloid cells, and the related factors in an imiquimod (IMQ)-induced skin psoriasis model. (**A**) Upper: representative images of dorsal skin treated topically with daily Vas/IMQ from 0–3 d (4 times). Lower: Clinical score such as erythema and scaling from the dorsal skin treated with Vas/IMQ (*n* = 3 each). 2way ANOVA/Sidak comparison: *p* = n.d. (0 d), *p* < 0.0001 (1 d), *p* = 0.0005 (2 d), *p* < 0.0001 (3 d), *p* = 0.0001 (4 d) for erythema, and *p* = n.d. (0 d), *p* < 0.0001 (1 d), *p* < 0.0002 (2 d), *p* < 0.0001 (3 d), *p* < 0.0001 (4 d) for scaling. The scale bars in each figure represent 1 cm long. (**B**) mRNA expression of dermatitis-related cytokines in dorsal skin treated with Vas (*n* = 3–5) or IMQ (*n* = 3–5). *t*-test/Mann–Whitney test: *p* = 0.0306 (*Il17a*), *p* = 0.0178 (*Il17f*), *p* > 0.9999 (*Il23*), *p* = n.d. (*Il22*), *p* = 0.0526 (*Tnfa*), *p* = 0.0079 (*Il6*). (**C**) Representative FACS gating strategy plots of myeloid cell fractions from dorsal skin treated with Vas or IMQ. (**D**) mRNA expression of chemokines in the dorsal skin treated with Vas or IMQ (*n* = 3–5 for each). *t*-test/Mann–Whitney test: *p* = 0.0079 (*Cxcl1*), *p* = 0.0159 (*Cxcl2*), *p* = 0.0500 (*Cxcl5*), *p* = 0.9758 (*Ccl2*). (**E**) Number and percentage of myeloid cell fractions in the non-lesional abdominal skin (Vas: *n* = 4, IMQ: *n* = 4); statistical analysis *t*-test: *p* = 0.6224 (Neutrophil), *p* = 0.0891 (Ly6C^+^), *p* = 0.1634 (Ly6C^-^) for cell number, *p* = 0.1255 (Neutrophil), *p* = 0.2411 (Ly6C^+^), *p* = 0.8402 (Ly6C^-^) for percentage. (**F**) Representative 3-dimensional (3D) intravital microscopic images of dorsal skin at 0 d, 1 d, and 2 d after Vas or IMQ treatment. Left images show raw data with Ly6G^+^ cells (red), CD31^+^ cells (green), and SHG (second harmonic generation) (blue). Right images show spot and surface transformed data with spot (Ly6G^+^) and surface (CD31^+^). (**G**) Representative images of the transformed 3D-intravital microscopy of neutrophil (left) and random spot (right) proximity distribution to blood vessel in the 2 d IMQ-skin, scale bar = 50 µm. (**H**) Bar graphs showing the comparative distribution of neutrophils and random spots to the blood vessel in the IMQ-skin at 0 d (upper) and 1 d of IMQ treatment (lower) (*n* = 3 for each). Data are pooled from ≥ 2 independent experiments with each dot in the graphs representing one experimental subject. *t*-test: *p* = 0.2027 (> 0–10), *p* = 0.2619 (> 10–20), *p* = 0.9444 (> 20–30), *p* = 0.3750 (> 30–40), *p* = 0.5581 (> 40–50), *p* < 0.0001 (> 50) for 0d-IMQ, *p* = 0.0338 (> 0–10), *p* = 0.0049 (> 10–20), *p* = 0.1529 (> 20–30), *p* = 0.0290 (> 30–40), *p* = 0.0572 (> 40–50), *p* = 0.0050 (> 50) for 1d-IMQ. For 3D-intravital microscopy, experiment was done in 1 subject per group with each dot in the graphs representing individual acquired tissue plane. Data are shown as mean ± S.E. *p < 0.05; **p < 0.01; ***p < 0.001; ****p < 0.0001.

We employed a well-established murine psoriasis model by topical application of a TLR7 agonist, imiquimod (IMQ) or vaseline control (Vas) for four consecutive days (Fig. 1A). This treatment induced acute psoriasis-like manifestations, including scaling, erythema, and epidermal thickening at the application site (Figs. 1B and EV1A), accompanied by increased expression of dermatitis-associated cytokines such as *Il17a, Il17f, Il22,* and *Il6* (Fig. EV1B) (van der Fits et al, 2009; Goodman et al, 2009). Flow cytometric analysis revealed that, among skin myeloid subsets, neutrophils were notably expanded following IMQ treatment (Fig. EV1C). Their numbers increased progressively, reaching approximately 10-fold by day 4 (4 d), whereas Ly6C^+^ and Ly6C^-^ myeloid cells were unchanged (Fig. 1C). Consistently, the neutrophil-attracting chemokines *Cxcl1, Cxcl2,* and *Cxcl5* were upregulated by 10–100-fold in the psoriatic skin, while *Ccl2*, a major monocyte chemoattract, remained unchanged (Fig. EV1D) (Metzemaekers et al, 2020; Deshmane et al, 2009). Neutrophil infiltration was restricted to IMQ-treated skin, as it was absent in untreated skin of the same animals (Fig. EV1E).

To further define neutrophil trafficking, we performed intravital 3D imaging in the dorsal skin of *Ly6G*^*Cre*^*;Rosa26*^*tdTom*^ mice in which neutrophils are labeled with tdTomato (Fig. 1D) (Hasenberg et al, 2015). The analysis was done between 0 d and 2 d due to high autofluorescence of plaques in the psoriatic skin after 2 d (Figs. 1E and EV1F). The CD31^+^ blood vessels expanded in parallel with neutrophil accumulation. At baseline (0 d), neutrophils were evenly distributed within 50 µm distance from the nearest vessels. By 1 d post psoriasis induction, ~40% of them are localized within 0–10 µm and another 40% within 10–20 µm. About 50% of neutrophils were concentrated within 0–10 µm and about 30% within 10–20 µm by 2 d, indicating progressive perivascular neutrophil clustering during psoriasis development (Figs. 1F and EV1G,H).

Collectively, these results suggest that neutrophils migrate to the skin and are retained in close proximity to blood vessels during psoriasis.

### Skin-infiltrating neutrophils contribute to psoriatic inflammation

**Figure 2:**
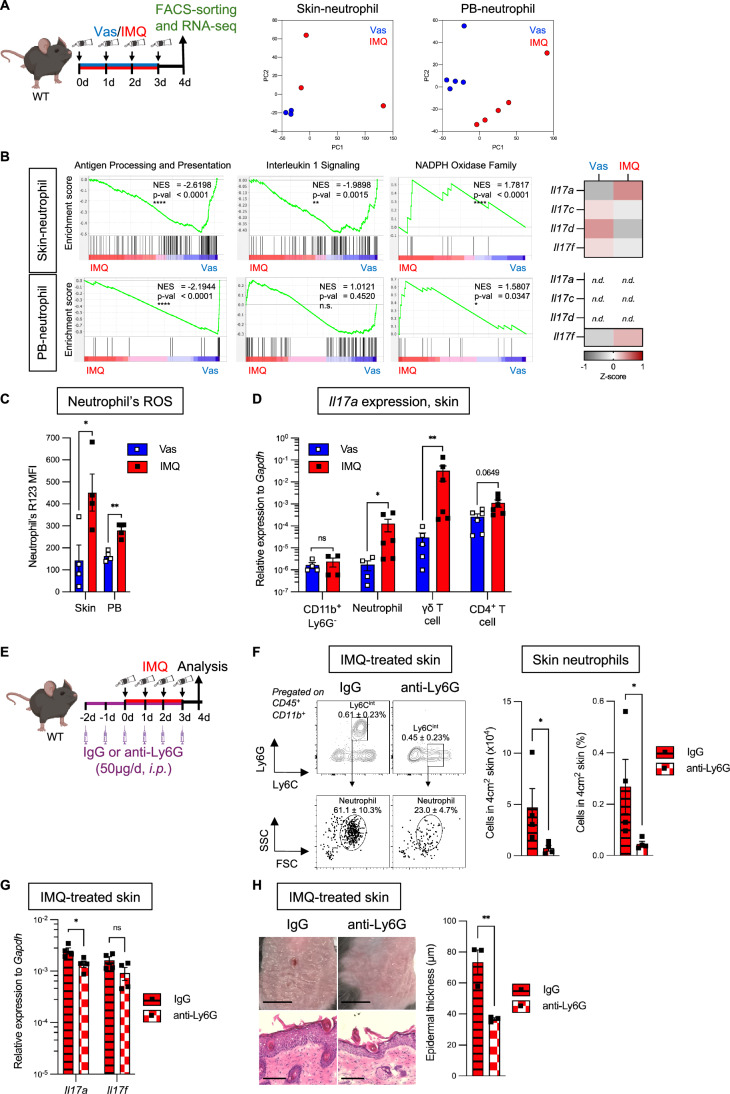
Neutrophils are primed for cutaneous inflammation in psoriasis condition. (**A**) Experimental scheme for bulk RNA-seq of skin- and PB-derived neutrophil and the resultant PCA plots. (**B**) GSEA and heatmap of *Il17* family gene in the neutrophils from Vas- and IMQ-treated skin and PB (*n* = 3–5 for each). Signal2Noise and FWER *p*-value: *p* < 0.0001 (Antigen Processing and Presentation), *p* = 0.0015 (Interleukin 1 Signaling), *p* < 0.0001 (NADPH Oxidase Family) for Skin neutrophils and *p* < 0.0001 (Antigen Processing and Presentation), *p* = 0.4520 (Interleukin 1 Signaling), *p* = 0.0347 (NADPH Oxidase Family) for PB neutrophils, *z* = −0.4452 (Vas)/0.4452 (IMQ) (*Il17a*), *z* = 0.1392 (Vas)/−0.1392 (IMQ) (*Il17c*), *z* = 0.4082 (Vas)/−0.4082 (IMQ) (*Il17d*), *z* = 0.1433 (Vas)/−0.1433 (IMQ) (*Il17f*) for skin neutrophils and *z* = −0.2842 (Vas)/0.2842 (IMQ) (*Il17f*) for PB neutrophils. (**C**) ROS level in the neutrophils from Vas- or IMQ-treated skin and PB (*n* = 4 for each). *t*-test: *p* = 0.0300 (Skin), *p* = 0.0011 (PB). (**D**) RT-PCR of *Il17a* from sorted skin-cells treated with Vas (*n* = 4–6) and IMQ (*n* = 4–6). Mann–Whitney test: *p* = 0.5562 (CD11b^+^ Ly6G^-^), *p* = 0.0381 (Neutrophil), *p* = 0.0043 (γδ T-cell), *p* = 0.0649 (CD4^+^ T-cell). (**E**) Experimental scheme of in vivo neutrophil depletion with anti-Ly6G neutralizing and IgG isotype-matched control antibodies which results are depicted in (**F**–**H**). Antibodies were injected once a day starting at 2 d before topical IMQ-treatment (−2 d) to 3 d (total 6x injections). Analysis was conducted at 4 d. (**F**) FACS analysis of skin neutrophils from IMQ-skin injected with neutralizing antibodies. Representative plot pre-gated on CD45^+^CD11b^+^ followed by the bar chart quantifying the absolute cell number and proportion of skin-neutrophils (IgG: *n* = 4; anti-Ly6G: *n* = 4). Mann–Whitney test: *p* = 0.0286 (cell number), *p* = 0.0286 (cell %). (**G**) RT-PCR results of *Il17a* and *Il17f* in the IMQ-skin injected with IgG (*n* = 4) and anti-Ly6G (*n* = 4). *t*-test: *p* = 0.0276 (*Il17a*), *p* = 0.1109 (*Il17f*). (**H**) Psoriasis clinical examination performed at the dorsal site. The dorsal skin images (upper panel: naked skin with bar indicating 1 cm; lower panel: histology with black line reflecting 100 µm). Epidermal thickness of the dorsal IMQ-skin intraperitoneally administered with IgG: *n* = 3; anti-Ly6G: *n* = 3). *t*-test: *p* = 0.0094. Data are pooled from ≥ 2 independent experiments with each dot shown in the chart represents the measurement from 1 experimental subject and shown as mean ± S.E. *p < 0.05; **p < 0.01, ****p < 0.0001, n.s., not significant. Source data are available online for this figure.

**Figure EV2:**
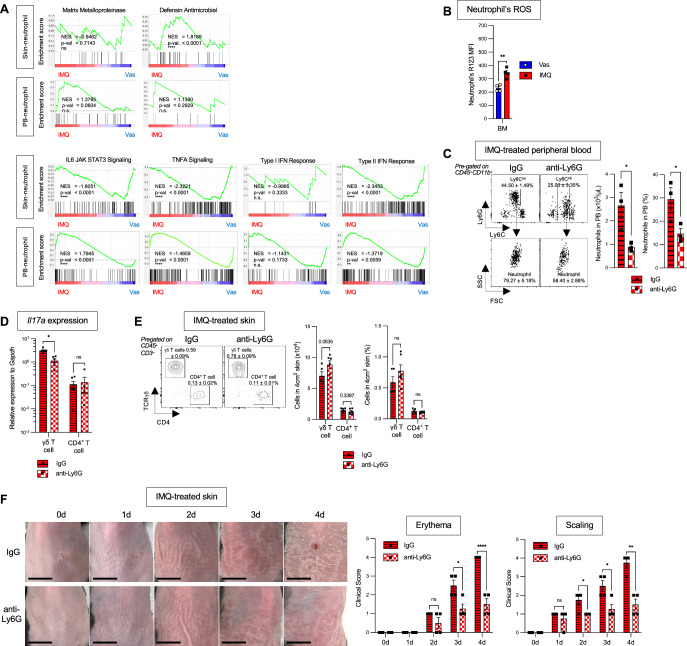
Overactive neutrophils have pathological function in psoriasis. (**A**) GSEA analysis of neutrophils from Vas-treated (*n* = 3–5) and IMQ-treated skin and PB (*n* = 3–5). Signal2Noise/FWER *p*-value: *p* = 0.7143 (Matrix Metalloproteinase), *p* < 0.0001 (Defensin Antimicrobial), *p* < 0.0001 (IL6 JAK STAT3 Signaling), *p* < 0.0001 (TNFA Signaling), *p* = 0.3333 (Type 1 IFN Response), *p* < 0.0001 (Type 2 IFN Response) for skin neutrophil and *p* = 0.0604 (Matrix Metalloproteinase), *p* = 0.02929 (Defensin Antimicrobial), *p* < 0.0001 (IL6 JAK STAT3 Signaling), *p* < 0.0001 (TNFA Signaling), *p* = 0.1733 (Type 1 IFN Response), *p* = 0.0059 (Type 2 IFN Response) for PB neutrophil. (**B**) ROS level in the neutrophils measured from BM of Vas- and IMQ-treated mice (*n* = 4 for each). *t*-test: *p* = 0.0023. (**C**) Representative FACS plot and quantification of peripheral blood neutrophils at 4 d from IgG or anti-Ly6G antibody injected IMQ-induced mice (*n* = 3 for each). Mann–Whitney test: *p* = 0.0286 (Neutrophil number), *p* = 0.0286 (Neutrophil percentage). (**D**) *Il17a* transcript measured in sorted phenotypic γδ T cells and CD4^+^ helper T cells of IMQ-treated mice injected with either IgG control or anti-Ly6G antibody (*n* = 5–6 for each). *t*-test: *p* = 0.0010 (γδ T-cell), *p* = 0.7979 (CD4^+^ T-cell). (**E**) Percentage and absolute cell number of γδ T cells and CD4^+^ helper T cells from IMQ-treated mice administered with either IgG control or anti-G-CSF antibody (*n* = 5–6 for each). *t-*test: *p* = 0.0836 (γδ T-cell), *p* = 0.3387 (CD4^+^ T-cell) for cell number, *p* = 0.2062 (γδ T-cell), *p* = 0.2695 (CD4^+^ T-cell) for percentage. (**F**) Clinical examination of dorsal IMQ-skin at 0-4 d with IgG or anti-Ly6G treatment. Upper: representative skin images, lower: erythema and scaling scoring (IgG: *n* = 4, anti-Ly6G: *n* = 4). *t*-test: *p* = n.d. (0 d), *p* = *n.d*. (1 d), *p* = n.d. (2 d), *p* = 0.0170 (3 d), *p* = 0.0001 (4 d) for erythema, *p* = n.d. (0 d), *p* = 0.3559 (1 d), *p* = 0.0240 (2 d), *p* = 0.0170 (3 d), *p* = 0.0011 (4 d) for scaling. The black line in each picture measures 1 cm scale. Data are pooled from ≥ 2 independent experiments with each dot shown in the bar graph represents data from each individual and shown as mean ± S.E. For RNA-seq, analysis was done to the data pooled from ≥2 replicates. *p < 0.05; **p < 0.01; ***p < 0.001; ****p < 0.0001.

To address the role of cutaneous neutrophils in psoriasis, we performed RNA-seq on neutrophils isolated from skin and peripheral blood of control (Vas-treated) and psoriatic (IMQ-treated) mice at 4 d post induction (Fig. 2A). Principal component analysis showed that IMQ-treated neutrophils were transcriptionally distinct from the closely-clustered Vas-treated neutrophils in both organs, indicating a psoriasis-associated transcriptional modulation. In both skin and peripheral blood, IMQ-treated neutrophils shared common signatures such as reduced antigen processing and presentation (*Fcgr1, Fcgr3, Rab6a, Ctsl*, etc.) and canonical pro-inflammatory cytokines network including TNFA Signaling (*Tnfs9, Tnfaip6, Stat5a, Fos*, etc.). Only the skin-derived neutrophil showed IL-1 (*Il1a, Il1b, Myd88, Irak1*, etc.) and IL-6 signaling (*Il6st, Socs1, Stat3, Pim1*, etc.) downregulation. The analysis also showed little changes in matrix metalloproteinase (*Mmp2, Mmp3, Mmp 7, Mmp9*, etc.) pathway in both tissues, while displaying a skin-specific defensin antimicrobial signals (*Defb7, Defb8, Defb21, Defb47*, etc.) upregulation (Figs. 2B and EV2A). Moreover, psoriatic neutrophils in both tissues upregulated genes involved in NADPH oxidase activity (*Nox1, Nox3, Nox4, Enox2*, etc.), a key circuit to ROS generation as common signatures (Meitzler et al, 2014). Notably, *Il17a* was selectively upregulated only in the cutaneous neutrophils, with significant downregulation of type II interferon response (*Ifng, Jak1, Stat1, Irf1*, etc.) and marginal alteration in type I interferon response *(Ifna1, Ifnb1, Ifnar1, Ifit1*, etc.), consistent with previous reports showing that functional secretion of IL-17A and IFNG are inversely related (Lee et al, 2013; Ajendra et al, 2020).

To validate the RNA-seq data, we first measured intracellular ROS level in the neutrophils from skin and peripheral blood. In steady state, the ROS level was comparable between skin- and peripheral blood-neutrophil (Fig. 2C). In contrast, psoriatic conditions significantly increased ROS level in both neutrophils (nearly four-fold and two-fold increase in skin- and peripheral blood-neutrophils, respectively). Of note, BM-derived neutrophils also showed a slight but significant increase (~50%) in ROS level under psoriasis condition (Fig. EV2B). We next compared *Il17a* expression across multiple immune cell populations in steady state and psoriatic skin (Fig. 2D). Steady state myeloid compartment (CD11b^+^ Ly6G^-^ and neutrophils) expressed low level of *Il17a*, unlike TCRγδ^+^ T cells (γδ T cells) or CD4^+^ helper T cells, both of which further upregulated *Il17a* in psoriasis condition, as shown in the previous reports (Blauvelt, 2008; Cai et al, 2011). Notably, psoriatic skin-derived neutrophils upregulated *Il17a* by ~100-fold, which was not seen in the rest of the myeloid cells (CD11b^+^ Ly6G^-^). These results suggest that upon psoriasis, neutrophils are primed to generate ROS in the BM and enhance ROS production through peripheral blood and skin, while *Il17a* expression is acquired predominantly upon their arrival in the skin.

To test the functional contribution of neutrophil to IL-17 signals, we depleted the neutrophil by daily injection of anti-Ly6G neutralizing antibody during IMQ application over 6 days (Fig. 2E). Because antibody binding masks Ly6G for immunophenotyping, neutrophils were quantified using size- and complexity-based gating (Fig. 2F). In line with the previous study (Wang et al, 2012), this approach could achieve around 80% depletion in skin and about 60% reduction in the circulating neutrophils (Fig. EV2C). Neutrophil depletion reduced *Il17a* expression in psoriatic skin (Fig. 2G). The global reduction was accompanied with reduced *Il17a* expression in γδ T cells, whereas its expression in CD4^+^ T cells remained unchanged (Fig. EV2D), despite no significant changes in their cell number (Fig. EV2E). These findings suggest that neutrophils promote *Il17a* expression in γδ T cells. Ultimately, neutrophil depletion alleviated psoriatic disease severity, reducing both clinical (scaling and erythema) and histological (epidermal thickening) manifestations by approximately 50% (Figs. 2H and EV2F).

Altogether, these data identify neutrophils as active effectors for psoriatic inflammation, characterized with their enhanced ROS production and *Il17a* induction.

### Psoriatic skin inflammation drives emergency granulopoiesis in the bone marrow

**Figure 3:**
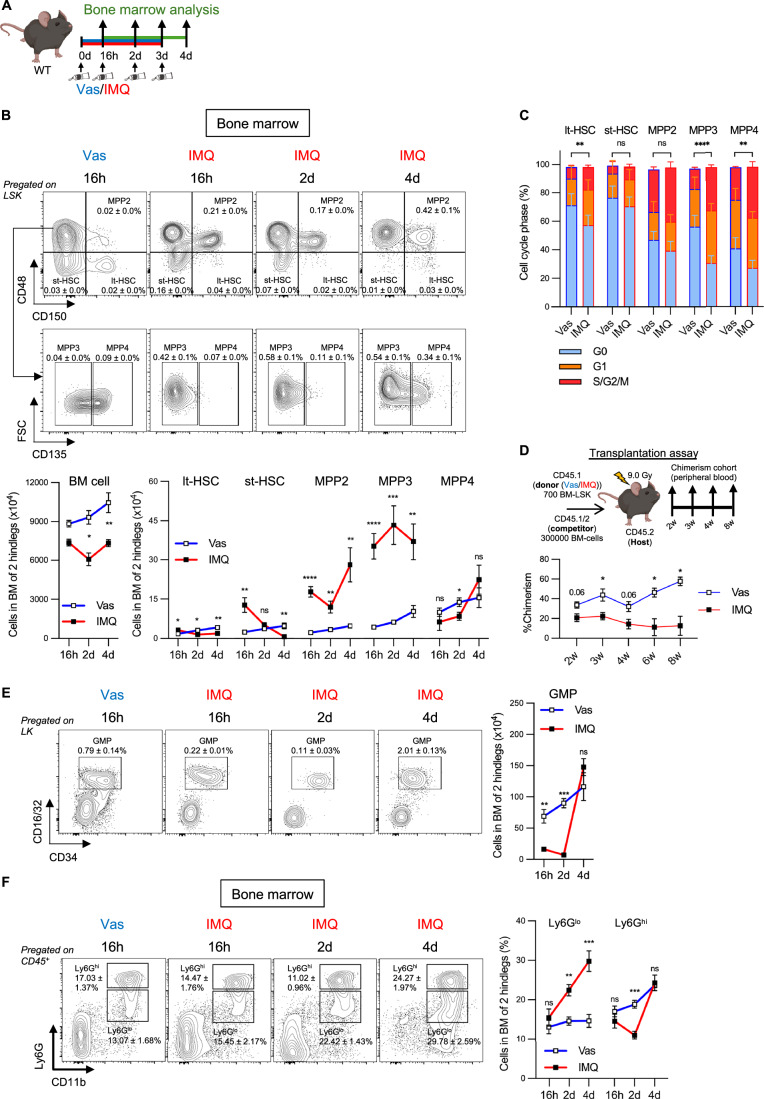
Emergency granulopoiesis activated in the psoriatic bone marrow. (**A**) Experimental scheme of BM analysis. Mice were topically treated with Vaseline (Vas) or Imiquimod (IMQ) at the dorsal skin site once/day at 0–3 d and analysis was done sequentially from different animals at 16 h, 2 d, and 4 d post-treatment. (**B**) FACS analysis of HSPC in the BM including the representative plots pregated on LSK cells in a time-course manner and the corresponding line plots depicting the absolute number of total BM cells, lt-HSC, st-HSC, MPP2, MPP3, MPP4 in the BM after treatment with topical Vas (*n* = 6) or IMQ (*n* = 6). *t*-test: *p* = 0.0172 (16 h), *p* = 0.0104 (2 d), *p* = 0.0182 (4 d) for total BM cells, *p* = 0.0224 (16 h), *p* = 0.0243 (2 d), *p* = 0.0018 (4 d) for lt-HSC, *p* = 0.0044 (16 h), *p* = 0.1638 (2 d), *p* = 0.0040 (4 d) for st-HSC, *p* < 0.0001 (16 h), *p* = 0.0057 (2 d), *p* = 0.0054 (4 d) MPP2, *p* < 0.0001 (16 h), *p* = 0.0006 (2 d), *p* = 0.0045 (4 d) for MPP3, *p* = 0.3384 (16 h), *p* = 0.0418 (2 d), *p* = 0.3308 (4 d) for MPP4. (**C**) Cell cycle analysis of HSPC fractions in Vas- and IMQ-treated BM (*n* = 5 each group). *t*-test for S/G2/M phase: *p* = 0.0034 (lt-HSC), *p* = 0.3330 (st-HSC), *p* = 0.0935 (MPP2), *p* < 0.0001 (MPP3), *p* = 0.0081 (MPP4). (**D**) Competitive transplantation with donor LSK from Vas- (*n* = 3) or IMQ-treated (*n* = 4) BM. *t*-test: *p* = 0.0596 (2w), *p* = 0.0239 (3w), *p* = 0.0554 (4w), *p* = 0.0224 (6w), *p* = 0.0135 (8w). (**E**) FACS analysis of GMP number in the BM treated with Vas (*n* = 3) or IMQ (*n* = 3). Representative plots pregated on LK cells in a time-course manner and the corresponding line plots depicting the GMP number. *t*-test: *p* = 0.0090 (16 h), *p* = 0.0005 (2 d), *p* = 0.2973 (4 d). (**F**) FACS analysis of immature (Ly6G^lo^) and mature (Ly6G^hi^) granulocyte in the BM. Representative plots pregated on CD45^+^ BM cells (left) and time-course kinetics proportion (right) of Ly6G^lo^ and Ly6G^hi^ BM cells after topical Vas (*n* = 6) or IMQ (*n* = 6) treatment. *t*-test: *p* = 0.4055 (16 h), *p* = 0.0013 (2 d), *p* = 0.0006 (4 d) for Ly6G^lo^, *p* = 0.2936 (16 h), *p* = 0.0002 (2 d), *p* = 0.7958 (4 d) for Ly6G^hi^. Data are pooled from ≥ 2 independent experiments with each dot shown in the chart represents the measurement from 1 experimental subject and shown as mean ± S.E. *p < 0.05; **p < 0.01; ***p < 0.001; ****p < 0.0001. Source data are available online for this figure.

**Figure EV3:**
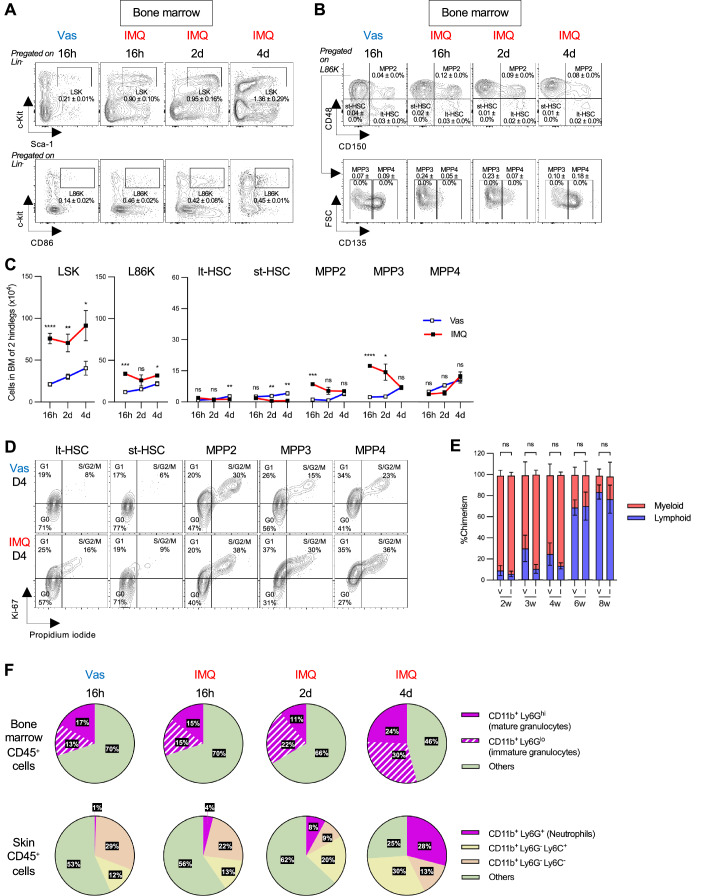
Psoriasis induces HSPC expansion and granulopoiesis. (**A**) FACS plots of LSK (Lin^-^ Sca-1^+^c-Kit^+^) (upper) and L86K (Lin^-^CD86^+^c-Kit^+^) cells (lower) in BM at 16 h, 2 d, and 4 d after Vas or IMQ treatment (*n* = 3–6 for each). (**B**) FACS plot of immunophenotypic lt-HSC, st-HSC, MPP2, MPP3, and MPP4 defined by L86K (EV3A, lower panel) in the BM of mice treated with Vas/IMQ (*n* = 3–6 each). (**C**) Absolute number of LSK (left), L86K (middle) and L86K-defined HSPC (lt-HSC, st-HSC, MPP2-4) (right) in the BM of mice treated with Vas/IMQ (*n* = 3–6 for each). *t*-test: *p* < 0.0001 (16 h), *p* = 0.0041 (2 d), *p* = 0.0279 (4 d) for LSK, *p* = 0.0009 (16 h), *p* = 0.1934 (2 d), *p* = 0.0239 (4 d) for L86K, *p* = 0.0677 (16 h), *p* = 0.8633 (2 d), *p* = 0.0059 (4 d) for lt-HSC, *p* = 0.1845 (16 h), *p* = 0.0050 (2 d), *p* = 0.0017 (4 d) for st-HSC, *p* = 0.0001 (16 h), *p* = 0.0546 (2 d), *p* = 0.3169 (4 d) for MPP2, *p* < 0.0001 (16 h), *p* = 0.0405 (2 d), *p* = 0.8809 (4 d) for MPP3, *p* = 0.2698 (16 h), *p* = 0.0536 (2 d), *p* = 0.4749 (4 d) for MPP4. (**D**) Representative FACS plots of cell cycle analysis of the HSPC fractions in Vas- and IMQ-treated BM (*n* = 5 for each). (**E**) Lympho-myeloid contribution in PB of transplants with donor LSK from Vas-treated (*n* = 3) or IMQ-treated (*n* = 4) BM. *t*-test: *p* = 0.5751 (2w), *p* = 0.1522 (3w), *p* = 0.2866 (4w), *p* = 0.9211 (6w), *p* = 0.7372 (8w) for Myeloid fraction. (**F**) Pie charts depicting relative composition of hematopoietic cells (CD45^+^) in BM (upper) and skin (lower) at 16 h, 2 d, and 4 d after Vas or IMQ treatment (*n* = 3–6 each). Data are pooled from ≥ 2 independent experiments. Each dot shown in the bar graph represents data from each individual and shown as mean ± S.E. *p < 0.05; **p < 0.01; ***p < 0.001; ****p < 0.0001.

Neutrophils are short-lived innate immune cells that rapidly respond to tissue insults and initiate downstream immune responses. Upon antigenic clearance, neutrophil demand rises sharply and is met through emergency granulopoiesis in the BM (Summers et al, 2010; Manz and Boettcher, 2014). Given that psoriatic skin is infiltrated by a large number of neutrophils (Fig. 1A–C), we reasoned that BM hematopoiesis might be activated to meet the demand. To test this hypothesis, we first examined early hematopoietic compartment in BM during psoriasis (Fig. 3A), including hematopoietic stem and progenitor cell (HSPC) fractions defined as Lin^-^c-Kit^+^Sca-1^+^ (LSK) or Lin^-^c-Kit^+^CD86^+^ (L86K) for alternative immunophenotyping under inflammatory conditions (Kanayama et al, 2020). BM cellularity was reduced by 20–30% across the psoriasis induction (Fig. 3B), while LSK-defined HSPCs expanded as early as 16 h post-psoriasis induction (Fig. EV3A,C). LSK CD150^+^CD48^-^ long term HSC (lt-HSC) slightly increased within 16 h and reduced toward 4 d, whereas LSK CD150^-^CD48^-^ short term HSC (st-HSC) greatly increased at 16 h and decreased sharply thereafter (Fig. 3B). The myeloid-biased multipotent progenitor (MPP2/MPP3) but not lymphoid-biased MPP4 (Pietras et al, 2015) rapidly expanded by 5- to 9-fold at 16 h post IMQ treatment and remained elevated during the disease course (Fig. 3B). Similarly, L86K-defined HSPC profiling showed an early increase of myeloid-biased MPP2/MPP3 reaching more than 5-fold increase under psoriasis conditions, with an overall HSC reduction and unchanged MPP4 number (Fig. EV3B,C). The numerical increase of HSPCs upon psoriasis was accompanied by more proliferation as cell cycle analysis shows (Figs. 3C and EV3D). Consistently with the forced proliferation, engrafting and repopulating ability of psoriatic HSPCs were diminished with no sign of lineage skewing after their transplantation into lethally irradiated recipients (Figs. 3D and EV3E), as shown by our previous report (Takizawa et al, 2011). Moreover, we observed a marked reduction of myeloid-committed GMPs by 2 d, followed by a rapid increase to a level slightly above the basal level at 4 d (Fig. 3E). These results suggest that the early hematopoiesis in the BM rapidly responds to distal skin inflammation and is shifted toward myelopoiesis, similar to those are shown upon systemic inflammation (Caiado et al, 2021).

We next analyzed granulopoiesis in BM which de novo generates neutrophils. Psoriasis induction expanded Ly6G^lo^ immature granulocytes by 2–3-fold within 4 d, while reducing Ly6G^hi^ mature granulocytes to ~40% of baseline at 2 d which was returned back to normal by 4 d (Fig. 3F). In the steady state skin, myeloid population accounts for less than half of CD45^+^ cells, with only about 1% neutrophil residing (Fig. EV3F). At 16 h post psoriasis induction neutrophils reached 4%, further expanded to 8% at 2 d, and 28% of the total hematopoietic (CD45^+^) compartment by 4 d. Concurrently, CD11b^+^ granulocytes in the BM progressively expanded comprising immature fraction from 13% to 30% and mature granulocytes from 17 to 24% of total BM cells at 4 d after psoriasis induction. The synchronous expansion of BM and skin granulocytes indicates a central role of BM to generate and directionally efflux neutrophils to skin upon psoriasis.

Taken together, these findings demonstrate that psoriatic skin inflammation activates emergency granulopoiesis in the BM, enforcing the cutaneous neutrophil burden in the inflamed skin.

### Endothelial cells in psoriatic skin produce G-CSF leading to its systemic elevation

**Figure EV4:**
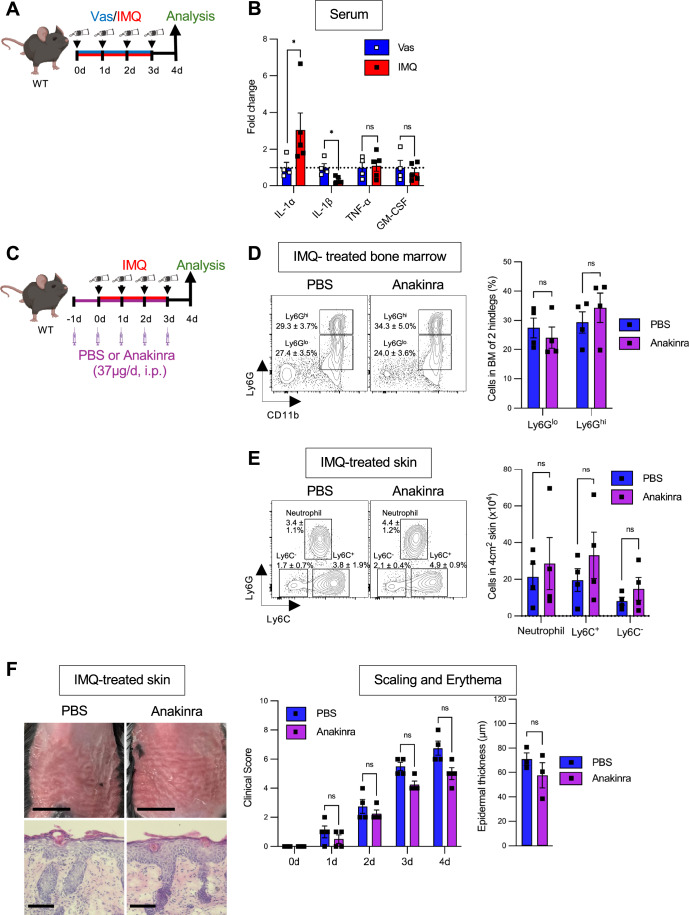
IL-1 signals do not contribute to psoriasis-driven emergency granulopoiesis. (**A**) Experimental scheme for (**B**): Vaseline (Vas) or Imiquimod (IMQ) topically applied to the dorsal skin daily for 4 d and serum analysis was conducted at 4 d. (**B**) Cytokines protein measurement from the sera (Vas: *n* = 4, IMQ: *n* = 5). *t*-test/Mann–Whitney test: *p* = 0.0317 (IL-1α), *p* = 0.0164 (IL-1β), *p* = 0.7905 (TNF-α), *p* = 0.5896 (GM-CSF). (**C**) Experimental scheme of IMQ-induced psoriasis (0–3 d) with daily PBS/Anakinra intraperitoneal injection (from −1 d to 3 d) and analyzed at 4 d for results depicted in (**D**–**H**). (**D**) Representative FACS plots of immature (Ly6G^lo^) and mature (Ly6G^hi^) granulocytes pregated on CD45^+^ BM cells (left). The proportion of Ly6G^lo^ and Ly6G^hi^ cells (*n* = 4 for each) (right). *t*-test: *p* = 0.5179 (Ly6G^lo^), *p* = 0.4510 (Ly6G^hi^). (**E**) Representative FACS plots of myeloid cell fractions in the dorsal skin pregated on CD45^+^CD11b^+^ cells (left). Cell number quantification graph of the IMQ-induced mice treated with PBS/Anakinra (*n* = 4 for each) (right). *t*-test: *p* = 0.6624 (Neutrophil), *p* = 0.3730 (Ly6C^+^), *p* = 0.3453 (Ly6C^-^). (**F**) Skin clinical examination upon psoriasis disease course. Representative naked skin images with scale bar = 1 cm (left upper panel) and skin histology by HE staining with black line indicates 100 µm (left lower panel). Daily clinical scoring (middle) and epidermal thickness measurement of IMQ-induced psoriatic skin following treatment with PBS/Anakinra (*n* = 3–4 for each). *t*-test/Mann–Whitney test: *p* > 0.9999 (0 d), *p* = 0.6571 (1 d), *p* = 0.7143 (2 d), *p* = 0.0857 (3 d), *p* = 0.0857 (4 d) for clinical score, *p* = 0.3123 for epidermal thickness. Data are pooled from ≥ 2 independent experiments with each dot shown in the bar chart represents individual subject and shown as mean ± S.E. *p < 0.05; **p < 0.01; ***p < 0.001; ****p < 0.0001.

Emergency granulopoiesis is driven by myeloid-supporting cytokines (Manz and Boettcher, 2014; Ueda et al, 2009). To identify the mediators of skin-BM crosstalk during psoriasis, we tested various pro-inflammatory cytokines in the serum of psoriatic mice (Fig. EV4A). Among those tested, IL-1α increased by approximately 3-fold whereas IL-1β decreased by half. Tumor necrosis factor-α (TNF-α) and GM-CSF remained unchanged (Fig. EV4B). Since IL-1α is known to induce granulopoiesis (Pietras et al, 2016), we blocked IL-1 signaling by daily injection of an IL-1 receptor (IL-1R) antagonist, Anakinra, over 4 consecutive days (Fig. EV4C) (Kovtonyuk et al, 2022; Wang et al, 2023). The IL-1R blockade did not significantly affect neutrophil expansion in BM and skin (Fig. EV4D,E), nor psoriasis pathology including gross (scaling and erythema) and histological (epidermal thickness) manifestations (Fig. EV4F). These results indicate that IL-1 signaling does not play an important role for psoriasis-associated skin-BM crosstalk.

**Figure 4:**
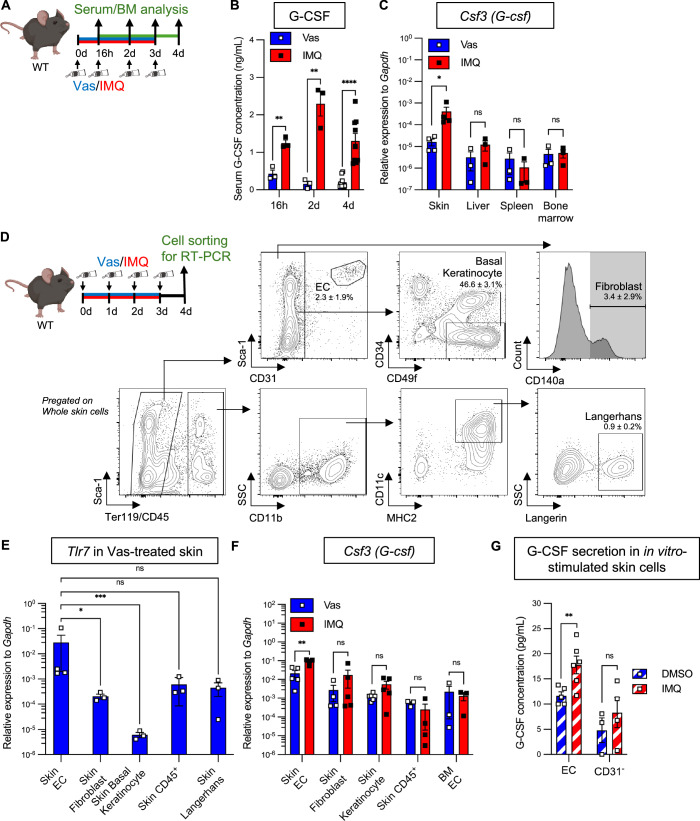
Psoriasis induces G-CSF expression in skin-resident endothelial cells. (**A**) Experimental scheme of serum and BM analysis for results depicted in (**B**, **C**): Mice were treated with Vas or IMQ once/day at the dorsal skin at 0–3 d and analysis were done successively at 16 h, 2 d, and 4 d with different mice from each time-point. (**B**) Time-course kinetics of serum G-CSF concentration after treatment with Vas (*n* = 3–9) or IMQ (*n* = 3–9). *t*-test: *p* = 0.0032 (16 h), *p* = 0.0030 (2 d), *p* < 0.0001 (4 d). (**C**) Relative expression of *Csf3 (G-csf)* mRNA in skin, liver, spleen, and BM at 4 d post-treatment with Vas (*n* = 3–4) or IMQ (*n* = 3–4). Mann–Whitney test: *p* = 0.0032 (Skin), *p* = 0.2000 (Liver), *p* = 0.4000 (Spleen), *p* > 0.9999 (BM). (**D**) Experimental scheme and the representative gating strategy for skin cell subtype by flowcytometric sorting at 4 d after topical Vas/IMQ treatment which results are depicted in (**E**, **F**): a hundred cells were sorted for each subtype (endothelial cells (ECs), basal keratinocyte, fibroblast, Langerhans cells). (**E**,** F**) Relative expression of *Tlr7* (**E**). and *G-csf* (**F**) mRNA (Vas: *n* = 3–6, IMQ: *n* = 3–5) from the cellular subsets mentioned in the respective bar graphs. Kruskal–Wallis, Dunn test for *Tlr7*: *p* = 0.0247 (EC vs fibroblast), *p* = 0.0006 (EC vs basal keratinocyte), *p* = 0.1554 (EC vs CD45^+^), *p* = 0.0899 (EC vs Langerhans) and *t*-test for *G-csf*: *p* = 0.0024 (Skin EC), *p* = 0.3867 (Skin Fibroblast), *p* = 0.0855 (Skin Keratinocyte), *p* = 0.3457 (Skin CD45^+^), *p* = 0.7174 (BM EC). (**G**) Sixty thousand cells were sorted and planted into culture media with or without IMQ (50 µg/mL) for 48 h and G-CSF was measured from the media subsequently (*n* = 3–6). Mann–Whitney test: *p* = 0.0173 (EC), *p* = 0.4000 (CD31^-^). Data are pooled from ≥ 2 independent experiments with each dot shown in the chart represents the measurement from 1 experimental subject and shown as mean ± S.E. *p < 0.05; **p < 0.01; ***p < 0.001; ****p < 0.0001. Source data are available online for this figure.

We next examined G-CSF, a key driver of granulopoiesis (Fig. 4A). G-CSF protein level in the serum rose sharply, about 5-fold at 16 h and up to 15-fold by 2 d to 4 d post psoriasis induction, concurrent with the upstream HSPC activation (Figs. 4B, 3B, and EV3A). To identify which organ produces G-CSF upon psoriasis, we compared *Csf3* (*G-csf*) gene expression in skin, liver, spleen, and BM at 4 d (Boettcher et al, 2014), and found that only skin showed a significant upregulation of *Csf3* in psoriatic conditions (Fig. 4C).

To identify what skin cell subtypes contribute to psoriasis-induced G-CSF secretion, we flow-cytometrically sorted specific skin cell populations including endothelial cells (ECs), fibroblasts, basal keratinocytes, CD45^+^ hematopoietic cells, and Langerhans cells and compared their gene expression (Fig. 4D). *Tlr7* (IMQ receptor) expression was the highest in skin ECs (>100-fold higher than other subsets) in steady-state skin (Fig. 4E). *Csf3* expression was significantly higher in steady-state skin ECs than other cells tested (*p* < 0.05) and significantly upregulated in skin ECs upon psoriasis (Fig. 4F). Of note, the BM-ECs showed no significant change of *Csf3* expression even after psoriasis induction, indicating that G-CSF production induced by psoriasis is skin specific.

To directly test the capacity of skin ECs to secrete G-CSF in response to IMQ treatment, ECs and CD31^-^ cells were sorted from steady-state skin and cultured in the presence or absence of IMQ for 2 days. In the absence of IMQ, G-CSF secretion by cultured skin ECs was more than twice of skin CD31^-^ cells. Upon IMQ stimulation, skin ECs increased G-CSF secretion by approximately 50% over baseline, whereas skin CD31^-^ cells did not show any increase at all (Fig. 4G). These results suggest that skin-resident ECs are a major producer of G-CSF in response to TLR7 stimulation.

Taken together, skin-resident ECs are able to respond to a TLR7 agonist via its receptor expression and readily secrete G-CSF upon stimulation, possibly contributing to its systemic elevation during psoriasis.

### Blockade of emergency granulopoiesis mitigates psoriasis

**Figure 5:**
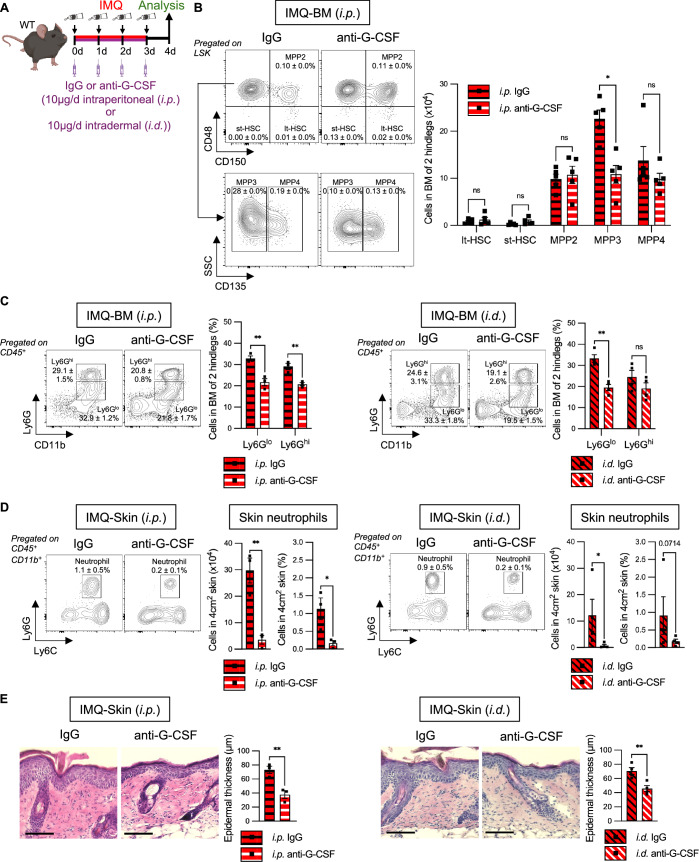
Inhibition of G-CSF-mediated granulopoiesis alleviates psoriasis symptoms. (**A**) Experimental scheme of G-CSF neutralization (**B**–**E**): mice were *i.p*. or *i.d*. pre-injected with anti-G-CSF neutralizing or IgG isotype-matched control antibodies 30 min before daily topical-IMQ application for 4 consecutive days (0–3 d). Analysis was performed at 4 d post-treatment. (**B**) FACS analysis of the BM showing representative FACS plot (left) and bar graphs (right) of absolute cell numbers of phenotypic lt-HSC, st-HSC, MPP2, MPP3, and MPP4 cells pre-gated on BM LKS co-treated with IMQ and with IgG (*n* = 5) or anti-G-CSF (*n* = 5). *t*-test: *p* = 0.5135 (lt-HSC), *p* = 0.1483 (st-HSC), *p* = 0.6865 (MPP2), *p* = 0.0022 (MPP3), *p* = 0.2571 (MPP4). (**C**) FACS analysis of immature (Ly6G^lo^) and mature (Ly6G^hi^) granulocytes pregated on CD45^+^ BM cells. Representative FACS plot (left) and proportion (right) of both immature (Ly6G^lo^) and mature (Ly6G^hi^) granulocyte fractions from IMQ-BM treated with *i.p*. (left) or *i.d*. (right) injection of IgG (*n* = 3–4) or anti-G-CSF (*n* = 3–4). *t*-test: *p* = 0.0062 (Ly6G^lo^), *p* = 0.0073 (Ly6G^hi^) for *i.p*. and *p* = 0.0010 (Ly6G^lo^), *p* = 0.2238 (Ly6G^hi^) for *i.d*. (**D**) FACS analysis of skin-resident neutrophils pre-gated on CD45^+^CD11b^+^ cells from dorsal skin. Representative FACS plot (left), number (middle) and proportion (right) of neutrophils in the skin co-treated with IMQ and *i.p*. (left) or *i.d*. (right) injection of IgG (*n* = 3–4) or anti-G-CSF (*n* = 3–4). *t*-test/Mann–Whitney test: *p* = 0.0031 (Cell number), *p* = 0.0188 (Cell %) for *i.p*., *p* = 0.0286 (Cell number), *p* = 0.0714 (Cell %) for *i.d*. (**E**) Representative naked skin images with scale bar of 1 cm (left upper panel) and skin histology with HE staining (lower panel, black line indicates 100 µm) (left lower panel). Quantification of epidermal thickness in skin co-treated with IMQ and *i.p*. (left) or *i.d*. (right) injection of IgG (*n* = 3–4) or anti-G-CSF (*n* = 3–4) (right). *t*-test: *p* = 0.0096 (*i.p*.), *p* = 0.0089 (*i.d*.). Data are pooled from ≥ 2 independent experiments with each dot shown in the bar chart represents the measurement from 1 experimental subject and shown as mean ± S.E. *p < 0.05; **p < 0.01. Source data are available online for this figure.

**Figure EV5:**
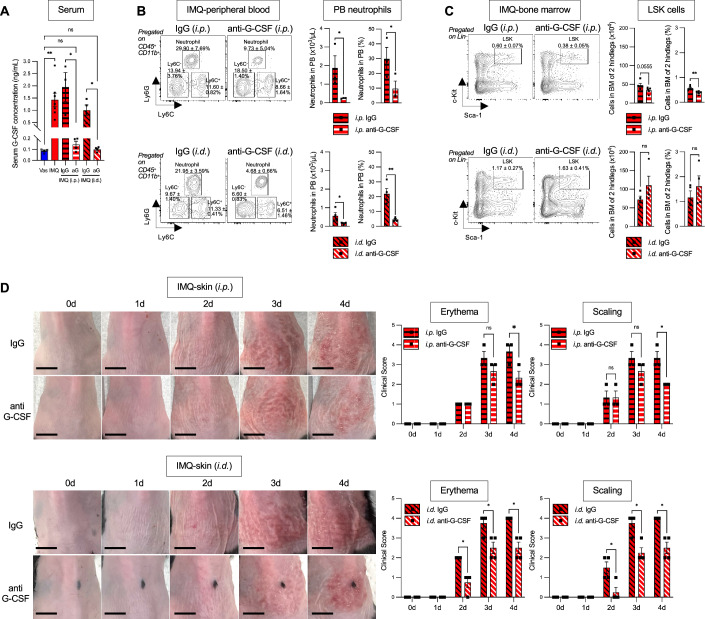
G-CSF neutralization reduces circulating neutrophils, hematopoietic activation in the BM, and psoriasis clinical manifestation. (**A**) G-CSF measured in the serum of mice with the following treatments (from left to right): Vas alone (*n* = 4), IMQ alone (*n* = 7), *i.p*. IgG Ab + IMQ (*n* = 3), *i.p*. anti-G-CSF Ab + IMQ (*n* = 4), *i.d*. IgG Ab + IMQ (*n* = 4), and *i.d*. anti-G-CSF Ab + IMQ (*n* = 4). Kruskal–Wallis test/Dunn’s test: *p* = 0.0010 (Vas vs IMQ), *p* = 0.4054 (Vas vs IMQ + anti-G-CSF *i.p*.), *p* = 0.6774 (Vas vs IMQ + anti-G-CSF *i.d*.), *p* = 0.0312 (IMQ + IgG *i.p*. vs IMQ + anti-G-CSF *i.p*.), *p* = 0.0468 (IMQ + IgG *i.d*. vs IMQ + anti-G-CSF *i.d*.). (**B**) Representative FACS plot along with the proportional and absolute cell number of circulating phenotypic neutrophils bar chart in IMQ-induced psoriasis mice treated with IgG/anti-GCSF (*n* = 3–4/group). *t*-test/Mann–Whitney test: *p* = 0.0050 (IgG *i.p*. vs anti-G-CSF *i.p*.), *p* = 0.0286 (IgG *i.d*. vs anti-G-CSF *i.d*.) for cell number and *p* = 0.0046 (IgG *i.p*. vs anti-G-CSF *i.p*.), *p* = 0.0032 (IgG *i.d*. vs anti-G-CSF *i.d*.) for percentage. (**C**) FACS analysis of the Lin^-^c-Kit^+^Sca-1^+^ (LSK) in the BM of IMQ-induced psoriasis treated with IgG/anti-G-CSF (*n* = 4–5 each). *t*-test/Mann–Whitney test: *p* = 0.0555 (IgG *i.p*. vs anti-G-CSF *i.p*.), *p* = 0.1878 (IgG *i.d*. vs anti-G-CSF *i.d*.) for cell number and *p* = 0.0080 (IgG *i.p*. vs anti-G-CSF *i.p*.), *p* = 0.3839 (IgG *i.d*. vs anti-G-CSF *i.d*.) for percentage. (**D**) Psoriasis clinical manifestation of the IMQ-induced mice which was monitored daily at the naked dorsal skin (upper) and clinically (erythema and scaling) graphed (lower) with *i.p*. (top panel) or *i.d*. (bottom panel) injection of IgG/anti-G-CSF (*n* = 3–4 for each). *t*-test: *p* = n.d. (0 d), *p* = *n.d*. (1 d), *p* = n.d. (2 d), *p* = 0.2302 (3 d), *p* = 0.0474 (4 d) for erythema, *i.p*., *p* = n.d. (0 d), *p* = n.d. (1 d), *p* > 0.9999 (2 d), *p* = 0.2302 (3 d), *p* = 0.0161 (4 d) for scaling, *i.p*., *p* = n.d. (0 d), *p* = *n.d*. (1 d), *p* = 0.0025 (2 d), *p* = 0.0170 (3 d), *p* = 0.0020 (4 d) for erythema, *i.d*., *p* = n.d. (0 d), *p* = n.d. (1 d), *p* = 0.0170 (2 d), *p* = 0.0054 (3 d), *p* = 0.0020 (4 d) for scaling *i.d*. The line bar embedded in each picture signify 1 cm scale. Data are pooled from ≥ 2 independent experiments with each dot shown in the chart represents the measurement from 1 experimental subject and shown as mean ± S.E. *p < 0.05; **p < 0.01; ***p < 0.001; ****p < 0.0001.

To evaluate the significant contribution of skin-derived G-CSF in psoriasis pathology, we systemically (intraperitoneally, *i.p*.) and locally (intradermally, *i.d*.) administered anti-G-CSF neutralizing antibody in vivo (Casanova-Acebes et al, 2018) during psoriasis induction (Fig. 5A). Psoriasis induction increased the serum G-CSF level by 15-fold which was normalized to a basal level effectively by both *i.p*. and *i.d*. injection of anti-G-CSF but not IgG control antibody (Fig. EV5A), confirming that skin-derived G-CSF is a major systemic contributor. Consistently, anti-G-CSF antibody treatment reduced circulating neutrophils in psoriatic mice by approximately 80% at 4 d post induction (Fig. EV5B). BM analysis revealed that systemic G-CSF blockade led to 30% suppression of psoriasis-induced HSPC expansion in BM, whereas no obvious effect was observed in local G-CSF blockage (Fig. EV5C). Specifically, systemic G-CSF inhibition diminished the myeloid-biased MPP3 expansion by about 60%, while the number of HSCs and other MPPs remained comparable (Fig. 5B).

As for emergency granulopoiesis, anti-G-CSF antibody treatment via both intraperitoneal and intradermal route similarly suppressed ca. 30% of psoriasis-induced granulocyte expansion for both Ly6G^lo^ immature and Ly6G^hi^ mature granulocytes (Fig. 5C). Consistent with BM granulopoiesis suppression by the treatments, neutrophil burden in psoriatic skin decreased by >80% (Fig. 5D), accompanied by reduced clinical (erythema, scaling) and histological (epidermal thickening) severity by about 50% (Figs. 5E and EV5D).

Collectively, our data suggest that skin-derived G-CSF is a critical factor in orchestrating pathological skin-BM coordination of cutaneous neutrophilic inflammation exemplified in psoriasis.

### Neutrophil activation and G-CSF expression in endothelial cells of human psoriatic skin

**Figure EV6:**
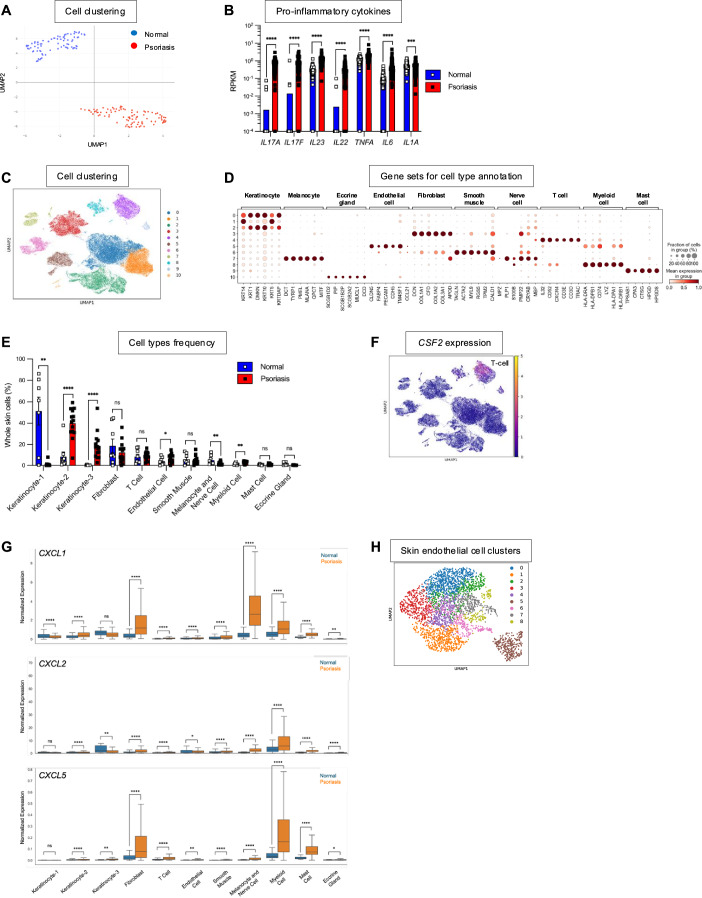
Clustering and relative transcript measurement of human psoriasis skin biopsies. (**A**) UMAP clustering of RNA-seq data from healthy and psoriatic human skin biopsy (normal: *n* = 82, psoriasis: *n* = 92). (**B**) Relative expression of dermatitis-related cytokines (normal: *n* = 82, psoriasis: *n* = 92). Mann–Whitney test: *p* < 0.0001 (*IL17A*), *p* < 0.0001 (*IL17F*), *p* < 0.0001 (*IL23*), *p* < 0.0001 (*IL22*), *p* < 0.0001 (TNF*A*), *p* < 0.0001 (*IL6*), *p* = 0.0007 (*IL1A*). (**C**–**H**) Re-analysis of single-cell RNA-seq dataset GSE173706 (normal: *n* = 8, psoriasis: *n* = 14) consisting of UMAP clustering (**C**), gene list for clusters annotation (**D**), cell frequency counting on each annotated cell types (**E**). *t*-test/Mann–Whitney test: *p* = 0.0023 (Keratinocyte-1), *p* < 0.0001 (Keratinocyte-2), *p* < 0.0001 (Keratinocyte-3), *p* = 0.1568 (Fibroblast), *p* = 0.4034 (T-cell), *p* = 0.0499 (Endothelial cell), *p* = 0.3319 (Smooth muscle), *p* = 0.0083 (Melanocyte and Nerve cell), *p* = 0.0017 (Myeloid cell), *p* = 0.4803 (Mast cell), *p* = 0.1128 (Eccrine gland), *CSF2* expression mapped in the UMAP clustering (**F**), *CXCL1/CXCL2/CXCL5* expression (**G**). *t*-test: *p* < 0.0001 (Keratinocyte-1), *p* < 0.0001 (Keratinocyte-2), *p* = 0.0813 (Keratinocyte-3), *p* < 0.0001 (Fibroblast), *p* < 0.0001 (T-cell), *p* < 0.0001 (Endothelial cell), *p* < 0.0001 (Smooth muscle), *p* < 0.0001 (Melanocyte and Nerve cell), *p* < 0.0001 (Myeloid cell), *p* < 0.0001 (Mast cell), *p* = 0.0045 (Eccrine gland) for *CXCL1*. *p* = 0.0602 (Keratinocyte-1), *p* < 0.0001 (Keratinocyte-2), *p* = 0.0032 (Keratinocyte-3), *p* < 0.0001 (Fibroblast), *p* < 0.0001 (T-cell), *p* = 0.0129 (Endothelial cell), *p* < 0.0001 (Smooth muscle), *p* < 0.0001 (Melanocyte and Nerve cell), *p* < 0.0001 (Myeloid cell), *p* < 0.0001 (Mast cell), *p* < 0.0001 (Eccrine gland) for *CXCL2*. *p* = 0.3313 (Keratinocyte-1), *p* < 0.0001 (Keratinocyte-2), *p* < 0.0001 (Keratinocyte-3), *p* < 0.0001 (Fibroblast), *p* < 0.0001 (T-cell), *p* = 0.0021 (Endothelial cell), *p* < 0.0001 (Smooth muscle), *p* < 0.0001 (Melanocyte and Nerve cell), *p* < 0.0001 (Myeloid cell), *p* < 0.0001 (Mast cell), *p* = 0.0390 (Eccrine gland) for *CXCL5*. Following are the detailed analytical data of each (value of Normal/value of Psoriasis): *CXCL1*: Keratinocyte-1 (min = 0.0054/0.0436, max = 2.8499/1.1675, median = 0.3033/0.2212, mean = 0.3706/0.2717, lower whisker = 0.0054/0.0436, upper whisker = 1.0234/0.6486, 95th percentile = 0.9001/0.5699), Keratinocyte-2 (min = 0.0231/0.0119, max = 1.8105/20.4381, median = 0.2590/0.4055, mean = 0.2933/0.5699, lower whisker = 0.0231/0.0119, upper whisker = 0.6936/1.3328, 95th percentile = 0.6271/1.5084), Keratinocyte-3 (min = 0.0607/0.0281, max = 1.2440/5.5007, median = 0.6806/0.4410, mean = 0.6481/0.5593, lower whisker = 0.0607/0.0281, upper whisker = 1.2440/1.2734, 95th percentile = 1.0515/1.4100), Fibroblast (min = 0.0366/0.0617, max = 5.4288/27.4380, median = 0.3219/1.1753, mean = 0.4593/1.9295, lower whisker = 0.0366/0.0617, upper whisker = 1.0833/5.3657, 95th percentile=1.2122/6.0698), T-Cell (min = 0.0062/0.0065, max = 0.5589/6.4854, median = 0.0340/0.0861, mean = 0.0474/0.1274, lower whisker = 0.0062/0.0065, upper whisker = 0.1099/0.2845, 95th percentile = 0.1260/0.3394), Endothelial Cell (min = 0.0032/0.0052, max = 1.2288/2.6625, median = 0.0688/0.0777, mean = 0.1035/0.1456, lower whisker = 0.0032/0.0052, upper whisker = 0.2650/0.3627, 95th percentile = 0.2890/0.5058), Smooth Muscle (min = 0.0034/0.0055, max = 2.2183/4.5404, median = 0.0847/0.1834, mean = 0.1913/0.3389, lower whisker = 0.0034/0.0055, upper whisker = 0.5502/0.8210, 95th percentile=0.7248/1.1467), Melanocyte and Nerve Cell (min = 0.0194/0.0716, max = 1.8830/16.2340, median = 0.4136/2.6095, mean = 0.4975/3.4355, lower whisker = 0.0194/0.0716, upper whisker = 1.2674/9.2033, 95th percentile = 1.2372/9.1966), Myeloid Cell (min = 0.0104/0.0032, max = 1.8229/19.2280, median = 0.4934/1.0720, mean = 0.5847/1.4393, lower whisker = 0.0104/0.0032, upper whisker = 1.2988/3.9222, 95th percentile = 1.3739/3.7889), Mast Cell (min = 0.0732/0.1191, max = 0.5190/10.0436, median = 0.2052/0.4905, mean = 0.2128/0.5765, lower whisker = 0.0732/0.1191, upper whisker = 0.4121/1.0925, 95th percentile = 0.3820/1.0138), Eccrine Gland (min = 0.0012/0.0017, max = 0.0873/0.3837, median = 0.0158/0.0232, mean = 0.0201/0.0424, lower whisker = 0.0012/0.0017, upper whisker = 0.0588/0.0966, 95th percentile = 0.0487/0.1275) for *CXCL1*; Keratinocyte-1 (min = 0.0054/0.0436, max = 2.8499/1.1675, median = 0.3033/0.2212, mean = 0.3706/0.2717, lower whisker = 0.0054/0.0436, upper whisker = 1.0234/0.6486, 95th percentile = 0.9001/0.5699), Keratinocyte-2 (min = 0.0231/0.0119, max = 1.8105/20.4381, median = 0.2590/0.4055, mean = 0.2933/0.5699, lower whisker = 0.0231/0.0119, upper whisker = 0.6936/1.3328, 95th percentile = 0.6271/1.5084), Keratinocyte-3 (min = 0.0607/0.0281, max = 1.2440/5.5007, median = 0.6806/0.4410, mean = 0.6481/0.5593, lower whisker = 0.0607/0.0281, upper whisker = 1.2440/1.2734, 95th percentile = 1.0515/1.4100), Fibroblast (min = 0.0366/0.0617, max = 5.4288/27.4380, median = 0.3219/1.1753, mean = 0.4593/1.9295, lower whisker = 0.0366/0.0617, upper whisker = 1.0833/5.3657, 95th percentile = 1.2122/6.0698), T-Cell (min=0.0062/0.0065, max = 0.5589/6.4854, median = 0.0340/0.0861, mean = 0.0474/0.1274, lower whisker = 0.0062/0.0065, upper whisker = 0.1099/0.2845, 95th percentile = 0.1260/0.3394), Endothelial Cell (min = 0.0032/0.0052, max = 1.2288/2.6625, median = 0.0688/0.0777, mean = 0.1035/0.1456, lower whisker = 0.0032/0.0052, upper whisker = 0.2650/0.3627, 95th percentile = 0.2890/0.5058), Smooth Muscle (min = 0.0034/0.0055, max = 2.2183/4.5404, median = 0.0847/0.1834, mean = 0.1913/0.3389, lower whisker = 0.0034/0.0055, upper whisker = 0.5502/0.8210, 95th percentile = 0.7248/1.1467), Melanocyte and Nerve Cell (min = 0.0194/0.0716, max = 1.8830/16.2340, median = 0.4136/2.6095, mean = 0.4975/3.4355, lower whiske r = 0.0194/0.0716, upper whisker = 1.2674/9.2033, 95th percentile = 1.2372/9.1966), Myeloid Cell (min = 0.0104/0.0032, max = 1.8229/19.2280, median = 0.4934/1.0720, mean = 0.5847/1.4393, lower whisker = 0.0104/0.0032, upper whisker = 1.2988/3.9222, 95th percentile = 1.3739/3.7889), Mast Cell (min = 0.0732/0.1191, max = 0.5190/10.0436, median = 0.2052/0.4905, mean = 0.2128/0.5765, lower whisker = 0.0732/0.1191, upper whisker = 0.4121/1.0925, 95th percentile = 0.3820/1.0138), Eccrine Gland (min = 0.0012/0.0017, max = 0.0873/0.3837, median = 0.0158/0.0232, mean = 0.0201/0.0424, lower whisker = 0.0012/0.0017, upper whisker = 0.0588/0.0966, 95th percentile = 0.0487/0.1275) for *CXCL2*; Keratinocyte-1 (min = 0.0054/0.0436, max = 2.8499/1.1675, median = 0.3033/0.2212, mean = 0.3706/0.2717, lower whisker = 0.0054/0.0436, upper whisker = 1.0234/0.6486, 95th percentile = 0.9001/0.5699), Keratinocyte-2 (min = 0.0231/0.0119, max = 1.8105/20.4381, median = 0.2590/0.4055, mean = 0.2933/0.5699, lower whisker = 0.0231/0.0119, upper whisker = 0.6936/1.3328, 95th percentile = 0.6271/1.5084), Keratinocyte-3 (min = 0.0607/0.0281, max = 1.2440/5.5007, median=0.6806/0.4410, mean = 0.6481/0.5593, lower whisker = 0.0607/0.0281, upper whisker = 1.2440/1.2734, 95th percentile = 1.0515/1.4100), Fibroblast (min = 0.0366/0.0617, max = 5.4288/27.4380, median = 0.3219/1.1753, mean = 0.4593/1.9295, lower whisker = 0.0366/0.0617, upper whisker = 1.0833/5.3657, 95th percentile = 1.2122/6.0698), T-Cell (min = 0.0062/0.0065, max = 0.5589/6.4854, median = 0.0340/0.0861, mean = 0.0474/0.1274, lower whisker = 0.0062/0.0065, upper whisker = 0.1099/0.2845, 95th percentile = 0.1260/0.3394), Endothelial Cell (min = 0.0032/0.0052, max = 1.2288/2.6625, median = 0.0688/0.0777, mean = 0.1035/0.1456, lower whisker = 0.0032/0.0052, upper whisker = 0.2650/0.3627, 95th percentile = 0.2890/0.5058), Smooth Muscle (min = 0.0034/0.0055, max = 2.2183/4.5404, median = 0.0847/0.1834, mean = 0.1913/0.3389, lower whisker = 0.0034/0.0055, upper whisker = 0.5502/0.8210, 95th percentile = 0.7248/1.1467), Melanocyte and Nerve Cell (min = 0.0194/0.0716, max = 1.8830/16.2340, median = 0.4136/2.6095, mean = 0.4975/3.4355, lower whisker = 0.0194/0.0716, upper whisker = 1.2674/9.2033, 95th percentile = 1.2372/9.1966), Myeloid Cell (min = 0.0104/0.0032, max = 1.8229/19.2280, median = 0.4934/1.0720, mean = 0.5847/1.4393, lower whisker = 0.0104/0.0032, upper whisker = 1.2988/3.9222, 95th percentile = 1.3739/3.7889), Mast Cell (min = 0.0732/0.1191, max = 0.5190/10.0436, median = 0.2052/0.4905, mean = 0.2128/0.5765, lower whisker = 0.0732/0.1191, upper whisker = 0.4121/1.0925, 95th percentile = 0.3820/1.0138), Eccrine Gland (min = 0.0012/0.0017, max = 0.0873/0.3837, median = 0.0158/0.0232, mean = 0.0201/0.0424, lower whisker = 0.0012/0.0017, upper whisker = 0.0588/0.0966, 95th percentile = 0.0487/0.1275) for *CXCL5*, and re-clustering of annotated endothelial cell subset (**H**). Each dot shown in the chart represents the measurement from 1 experimental subject and shown as mean ± S.E. *p < 0.05; **p < 0.01; ***p < 0.001; ****p < 0.0001.

**Figure 6:**
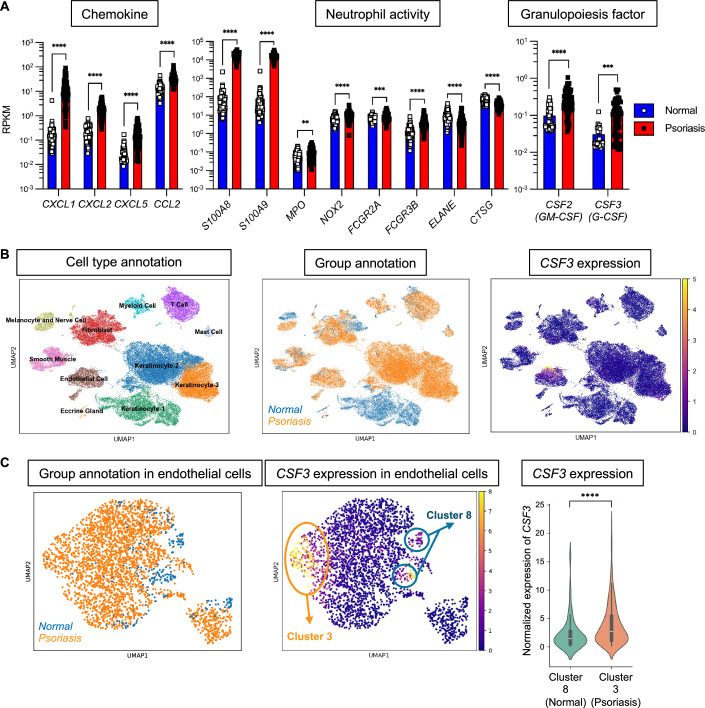
Human psoriatic skin shows neutrophil overactivity and elevated G-CSF expression in endothelial cells. (**A**) Expression of genes related to neutrophil function in the publicly available human psoriasis database (GSE54456). Each dot represents individual skin biopsy samples (normal: *n* = 23–82, psoriatic skin: *n* = 78–92). Expression of mRNA related to chemoattractant (left). *t*-test/Mann–Whitney test: *p* < 0.0001 (*CXCL1*), *p* < 0.0001 (*CXCL2*), *p* < 0.0001 (*CXCL5*), *p* < 0.0001 (*CCL2*) for chemokine, *p* < 0.0001 (*S100A8*), *p* < 0.0001 (*S100A9*), *p* = 0.0043 (*MPO*), *p* < 0.0001 (*NOX2*), *p* = 0.0007 (*FCGR2A*), *p* < 0.0001 (*FCGR3B*), *p* < 0.0001 (*ELANE*), *p* < 0.0001 (*CSTG*) for neutrophil activity (middle), and *p* < 0.0001 (*CSF2*), *p* = 0.0001 (*CSF3*) for granulopoiesis factor (right). *CSF*: colony-stimulating factor, *CTSG*: cathepsin-G, *CXCL*: CXC chemokine ligand, *ELANE*: neutrophil elastase, *FCGR2A: Fcγ*R*-2a, FCGR3B: Fcγ*R*-*3b*, IL*: interleukin, *MPO*: myeloperoxidase, *TNF*: tumor necrosis factor. (**B**) Clustering analysis of single-cell RNA-seq dataset (GSE173706) of the skin biopsies (normal: *n* = 8, psoriasis: *n* = 14) covering the annotation of the cell type clusters (left), annotation of the cluster’s disease condition (normal/psoriasis) (middle), and the differential expression of *CSF3* mRNA among all the annotated clusters (right). (**C**) Endothelial cell subsetting and re-analysis (GSE173706), covering group annotation of the in the endothelial cell subsets’ disease condition (normal, *n* = 8/psoriasis, *n* = 14) (left), differential expression analysis of *CSF3* (middle), and normalized *CSF3* expression of re-analyzed endothelial cells from cluster 8 (normal, *n* = 126 cells, min = 0.0913, max = 17.9352, median = 1.5369, mean = 2.4649, lower whisker = 0.0913, upper whisker = 5.1521, 95th percentile = 8.4958) and cluster 3 (psoriasis, *n* = 333 cells, min = 0.1421, max = 25.8366, median = 2.5728, mean = 3.8017, lower whisker = 0.1421, upper whisker = 11.9801, 95th percentile = 10.8099) (right). Mann–Whitney U test: *p* < 0.0001. Data are shown as mean ± S.E. *p < 0.05; **p < 0.01; ***p < 0.001; ****p < 0.0001. Source data are available online for this figure.

To assess whether our findings in the mouse model is relevant to human psoriasis, we re-analyzed public bulk RNA-seq dataset (GEO: GSE54456) containing nearly 100 skin biopsies of normal and psoriatic patients (Data ref: Li et al, 2014). The dataset showed clear segregation of normal and psoriatic samples, with a significant upregulation of hallmark dermatitis cytokines including around 3-log increase in *IL17A, IL17F, IL23*, and *IL22*, and 2-log increase in *TNFA* and *IL6* in the psoriatic skin biopsies (Fig. EV6A,B) (Griffiths et al, 2021; Armstrong and Read, 2020). The psoriatic skin showed upregulated expression of neutrophil-recruiting chemokines such as *CXCL1, CXCL2,* and *CXCL5* (≥ 10 time for all) (Capucetti et al, 2020), along with 5-time increase in *CCL2* (Fig. 6A). Moreover, a significant elevation of neutrophil activity-related genes was observed, such as activation molecules (*S100A8, S100A9*) by about 100-fold, oxidative activity (*MPO*, *NOX2*) by about 2-fold (Fig. 6A). An increase of phagocytic initiator *FCGR2A/FCGR3B* was observed, whereas serine protease *ELANE* and *CTSG* were found downregulated, suggesting an oxidative non-proteolytic phagocytosis action. Consistent with the findings in murine model, granulopoiesis-driving factors such as *CSF2* (*GM-CSF*) and* CSF3* (*G-CSF*) were elevated about 10-fold in psoriatic skin (Fig. 6A) (Naish et al, 2023). These results suggest an active recruitment and enhanced activity of neutrophil as well as increased level of neutrophil-supporting cytokine in human psoriatic skin.

To explore the underlying cellular mechanisms, we looked into a single-cell RNA-seq dataset (GEO: GSE173706) and annotated 11 distinct clusters including keratinocyte type 1–3, melanocyte, nerve, eccrine gland, endothelial cell, fibroblast, smooth muscle, T cell, myeloid cell, and mast cell (Figs. 6B and EV6C,D) (Data ref: Ma et al, 2023). The keratinocyte type 1 belongs to normal skin and is differentiated from type 2 and 3 found in psoriatic skin, a hallmark change in psoriatic skin previously reported (Figs. 6B and EV6E) (Zhou et al, 2022). Human psoriatic skin showed about double numerical expansion of both EC and myeloid cell compartments. *CSF3* expression was restricted to a subset of ECs, while *CSF2* expression was T cell specific (Figs. 6C and EV6F). Quantification of neutrophil-related chemokine expression, *CXCLs*, revealed that both stromal and immune compartment (fibroblast, melanocyte, and myeloid cells) but not ECs, upregulated those chemokines under psoriasis conditions (Fig. EV6G). We next focus on EC subset and re-clustered them from normal and psoriatic skin samples and found eight distinct clusters, two of which displayed *CSF3* expression with cluster 3 of psoriatic skin expressing it higher than cluster 8 of normal skin (Figs. 6C and EV6H).

Taken together, our findings suggest that human psoriatic skin is loaded with effector neutrophils that secrete psoriasis-mediating factors as well as activated ECs expressing G-CSF, analogous to IMQ-induced murine psoriasis model.

## Discussion

In this study, we demonstrate that (1) upon psoriasis, skin-resident endothelial cells are able to sense TLR7 agonist and secrete G-CSF, (2) which skews hematopoiesis in BM into emergency granulopoiesis, (3) concurrently, some neutrophils egress BM and progressively infiltrate into the inflamed skin through expanded blood vessels upon psoriasis, and (4) promote tissue inflammation via ROS production and/or IL-17A and worsening disease symptoms. (5) Depleting neutrophils or blocking G-CSF-mediated emergency granulopoiesis alleviates disease symptoms. (6) A similar mechanism might exist in human psoriasis.

Endothelial cells, the principal component of the vascular network, not only regulate leukocyte trafficking but also actively interact with immune cells to regulate their function (Amersfoort et al, 2022). ECs were reported to play a key role in neutrophil degranulation in vitro (Elbjeirami et al, 2011), whereas BM-resident ECs drive emergency granulopoiesis in response to infection via G-CSF augmentation in vivo (Boettcher et al, 2014; Basu et al, 2002). Our findings show that in psoriasis, skin-resident ECs act as a regulators for BM granulopoiesis through G-CSF production and cause granulopoiesis activation and cellular mobilization in the BM. This may add an additional mechanistic layer to the cross-organ communication upon epithelial barrier disruption as demonstrated in various disease models (Cataisson et al, 2006; Jackson et al, 2023). It is of note that since both *i.p*. and *i.d*. injection of anti-G-CSF neutralized systemic level of G-CSF (Fig. EV5A), skin is a major source of G-CSF upon psoriasis. Despite that ECs are a major G-CSF producer, other cell types may contribute to it as other cell type tested also show a trend to increase G-CSF expression upon activation (Fig. 4F,G). Given that G-CSF is a critical neutrophil survival factor (van Raam et al, 2008; Liu et al, 1996), the close proximity of neutrophils to ECs within the psoriatic lesion would likely prolong their lifespan, thus further promoting their excessive accumulation in the inflamed skin (Fig. 1E,F). Our findings highlight a detrimental role of skin-resident ECs as a niche for aberrant neutrophil function in driving psoriasis pathology via G-CSF production, a long-distant-traveling effector molecule that is remotely transmitted to the BM for granulocyte generation and mobilization, and at same time, locally act on neutrophils for prolonging their survival in the cutaneous milieu.

Our study shows that upon psoriasis, the G-CSF driven emergency hematopoiesis is characterized by rapid and sustained expansion of HSPCs, in particular to the myeloid-MPPs such as MPP2, and strongly to MPP3, which is granulocyte-bias (Figs. 3B and 5A) (Pietras et al, 2015). Apart of directly differentiating granulocyte trajectory, MPP3 may also function as a paracrine signal source to promote myelopoiesis under inflammatory stress (Kang et al, 2023). We expect that G-CSF blockade diminishes psoriasis-driven granulopoiesis in a pleiotropic way, firstly, by directly dampen G-CSF driven granulopoiesis, secondly, by secretory-MPP3’s absolute number, and potentially also functional attenuation. Furthermore, although psoriasis forces HSPC proliferation which impairs their engraftment upon transplantation, it didn’t alter the overall lineage output, suggesting that psoriasis induces their proliferative exhaustion rather than fate change (Takizawa et al, 2011; Essers et al, 2009). Additionally, the substantial reduction in BM cellularity and mature granulocytes (Fig. 3B,F) is partly due to G-CSF-driven granulocyte mobilization, which may generate a compensatory demand for further differentiation of the progenitors and de novo granulopoiesis (Bugl et al, 2013; Kang et al, 2020). Together, these findings support a model that psoriasis hematopoiesis is substantially driven by G-CSF.

Neutrophils harbor several modes of action covering cytokine secretion, oxidative burst, protease release, adaptive immunity induction, and NETosis which preferential initiation is highly dependent on microenvironmental cues (pathogen signals, neuroimmune mediators, metabolic signals, etc.) (Burn et al, 2021; Zhang et al, 2024). Our analysis shows that neutropoiesis in psoriasis model are primed for ROS production which would be the net results of chemoattractant, pro-inflammatory cytokines, or microbial signals sensing (El-Benna et al, 2016). High ROS production may induce DNA damage that compromises cellular function of surrounding cells and themselves, namely cytokine secretion (Harbort et al, 2015). This notion is in line with our observation that psoriatic neutrophils had a downregulation of canonical cytokine pathway, plausibly linked to their heightened superoxide level. An alternative possibility is that it may simply reflect an amplified ROS-loaded immature neutrophils generally observed in inflammatory granulopoiesis (Wang et al, 2020). The egress of immature neutrophils position them in a transitional functional state, thereby allowing further reprogramming/adaptation in target tissues (Ng et al, 2024). Our psoriatic skin indeed showed that skin-infiltrating neutrophils acquire IL-17A secretion property alongside IFNG downregulation. Given that psoriatic skin milieu is overloaded with pro-inflammatory cytokine, IL-23 (Griffiths et al, 2021), and that IL-23 license neutrophils for IL-17A production (Li et al, 2018; Furuya et al, 2024), we think that IL-23 may contribute to the observed functional shift in psoriatic neutrophils. Of note, downregulated IFNG signature supports this interpretation, as IL-17A and IFNG are inversely correlated across multiple model systems (Lee et al, 2013; Ajendra et al, 2020; Marié et al, 2021).

Among granulopoiesis-supporting factors, G-CSF and GM-CSF have overlapping but distinct effect on hematopoietic lineage instruction: GM-CSF broadly expands diverse myeloid cell types, whereas G-CSF selectively drives robust neutropoiesis (Welte et al, 1990). Anti-GM-CSF clinical trials in psoriasis reduced myeloid cell number but shows limited efficacy (Papp *et al*, 2019; Campione et al, 2021), whereas G-CSF supplementation increased neutrophil count which could trigger psoriasis dermatitis adverse events (Mössner et al, 2004; Sakashita et al, 2023; Jang et al, 2017; Feliu et al, 1997), suggesting a pathogenic role of neutrophil in the disease progression consistent with our findings. Downstream of G-CSF signaling, JAK-STAT inhibitors show clinical promise, achieving sustained reduction up to 75% for as long as 40 weeks (Furtunescu et al, 2024; Misra et al, 2025). Moreover, anti-TLR7 therapy for psoriasis has shown encouraging results and been advanced to clinical trials (Balak et al, 2017; Jeon et al, 2017; Jiang et al, 2013). Our findings add a mechanistic rationale for these observations by demonstrating that TLR7 sensing in skin ECs induces G-CSF secretion, driving BM-emergency granulopoiesis, leading to cutaneous neutrophil accumulation that aggravates psoriasis.

While many studies uncovered local cellular interactions in psoriatic skin, e.g., involving fibroblast, keratinocytes, and neutrophils (Cavagnero et al, 2024; Senra et al, 2016; Hao et al, 2023), emerging evidences suggests that psoriatic skin inflammation pertains to a systemic consequences through cross-organ communication. A study demonstrated that transgenic mice with keratinocyte-specific IL-17A overexpression developed chronic psoriasis and bone loss due to osteoblast-osteocyte dysfunction (Uluçkan et al, 2016). Moderate-to-severe psoriasis is also linked to myocardial infarction, likely driven by a combination of similar cytokines promoting both pathologies (Gelfand et al, 2006; Kivelevitch et al, 2017). Moreover, even acute psoriasis can cause increased susceptibility of developing gut colitis due to intestinal dysbiosis-driven gut macrophage hyperactivity (Pinget et al, 2022). In our study, we identified a pathological cross-organ feedback loop in psoriasis initiated by insulted skin ECs. Although psoriatic skin ECs are known to be functionally altered and promoting local T-cells infiltration (Li et al, 2023), we show that they also drive BM emergency granulopoiesis via G-CSF secretion, revealing a systemic role in skin-BM pathological crosstalk upon psoriasis.

Our findings suggest that the level of circulating G-CSF or the activity of skin-resident ECs could be used as biomarkers for disease severity and therapy response in psoriasis patients. It remains to be determined whether G-CSF driven skin-BM crosstalk is a distinctive feature of psoriasis or represents other skin inflammatory diseases involving acute neutrophil infiltration such as atopic dermatitis or pustular dermatoses (Chen et al, 2019; Naik and Cowen, 2013). Our study proposes the potential of targeting innate immunity as a complementary approach to existing biologics that primarily modulate adaptive immune pathways. Although IL-17 blockers (e.g., Secukinumab, Ixekizumab, Brodalumab) and IL-23 inhibitors (e.g., Guselkumab, Risankizumab, Tildrakizumab) show potential benefit, they provide limited control over neutrophil-driven inflammation (Reich et al, 2017, 2019; Langley et al, 2014; Papp et al, 2017). Given that neutrophils are key effector cells against infection, combining blockage of neutrophil infiltration into the skin with adaptive immune blockers might therefore enhance therapeutic efficacy and potentially prolong remission. Additionally, it would be informative to investigate other myeloid cells’ contribution to psoriatic inflammation, especially eosinophils or mast cells that show their promising roles as potent inflammatory orchestrator in concert with multiple cell types encompassing neutrophils and beyond (Park et al, 2024).

Lastly, we acknowledge several limitations of our study. The IMQ-psoriasis-like mouse model used here primarily represents acute psoriasiform inflammation and doesn’t fully recapitulate chronic human psoriasis. Moreover, psoriasis is a complex and heterogeneous disease comprising multiple clinical variants with distinct immunopathogenesis (Armstrong and Read, 2020; Griffiths et al, 2021). Our findings are constrained to TLR-mediated IL-17-driven cutaneous inflammation, and further studies using additional psoriasis models will be required to capture the broader spectrum of the disease (Bugl et al, 2013; Kang et al, 2020; Sumida et al, 2014).

## Methods

**Table:** Reagents and tools table

Reagent/Resource	Reference or Source	Identifier or Catalog Number
**Experimental models**		
C57BL6/J (*Mus musculus*)	Japan SLC/Jackson Laboratory	RRID: IMSR_JAX:000664
C57BL/6-*Ly6g*^tm2621(cre)Arte^ (*Mus musculus*)	TaconicArtemis	MGI:6330745
B6.Cg-*Gt(ROSA)26Sor*^*tm14(CAG-tdTomato)Hze*^/J (*Mus musculus*)	Jackson Laboratory	RRID: IMSR_JAX:007914
**Recombinant DNA**		
**Antibodies**		
Rat-anti-mouse biotin-anti-CD3e	BioLegend	Cat# 100304
Rat-anti-mouse biotin-anti-CD8a	BioLegend	Cat# 100704
Rat-anti-mouse biotin-anti-CD4	BioLegend	Cat# 100404
Mouse-anti-mouse biotin-anti-NK1.1	BioLegend	Cat# 108704
Rat-anti-mouse biotin-anti-CD127	BioLegend	Cat# 121104
Rat-anti-mouse biotin-anti-TER-119	BioLegend	Cat# 116204
Rat-anti-mouse biotin-anti-Gr-1	BioLegend	Cat# 108404
Rat-anti-mouse/human biotin-anti-CD11b	BioLegend	Cat# 101204
Rat-anti-mouse/human biotin-anti-CD45R/B220	BioLegend	Cat# 103204
Rat-anti-mouse purified-anti-CD16/32	BioLegend	Cat# 101302
Streptavidin BV605	BioLegend	Cat# 405229
Rat-anti-mouse BUV395-anti-CD45	BD Biosciences	Cat# 564279
Rat-anti-mouse BV421-anti-CD45	BioLegend	Cat# 103133
Mouse-anti-mouse FITC-anti-CD45.2	BioLegend	Cat# 109806
Rat-anti-mouse FITC-anti-CD34	Invitrogen/eBioscience	Cat# 11-0341-85
Armenian hamster-anti-mouse Pacific Blue-anti-CD48	BioLegend	Cat# 103418
Rat-anti-mouse APC-anti-CD117 (c-Kit)	BioLegend	Cat# 105812
Rat-anti-mouse PE-anti-CD150	BioLegend	Cat# 115904
Armenian hamster-anti-mouse PE/Cy7-anti-CD11c	BioLegend	Cat# 117318
Rat-anti-mouse PE/Cy7-anti-CD101	Invitrogen/eBioscience	Cat# 25-1011-80
Rat-anti-mouse BV605-anti-I-A/I-E	BioLegend	Cat# 107639
Rat-anti-mouse BV421-anti-CD31	BioLegend	Cat# 102423
Rat-anti-mouse APC-anti-CD140a	BioLegend	Cat# 135908
Rat-anti-human/mouse PE-anti-CD49f	BioLegend	Cat# 313611
Rat-anti-mouse PE/Cy5-anti-CD135	BioLegend	Cat# 135312
Rat-anti-mouse APC/Cy7-anti-Sca-1	BioLegend	Cat# 108126
Rat-anti-mouse BV785-anti-Ly6G	BioLegend	Cat# 127645
Rat-anti-mouse PE/Cy7-anti-Ly6G	BioLegend	Cat# 127618
Rat-anti-mouse BV711-anti-Ly6C	BioLegend	Cat# 128037
Rat-anti-mouse/human APC/Cy7-anti-CD11b	BioLegend	Cat# 101226
Mouse-anti-mouse/human PE-anti-CD207	BioLegend	Cat# 144204
Armenian hamster-anti-mouse PE/Cy7-anti-CD3e	BioLegend	Cat# 100320
Rat-anti-mouse BUV395-anti-CD4	BD Biosciences	Cat# 563790
Hamster-anti-mouse BV421-anti-γδTCR	BD Biosciences	Cat# 562892
InVivoMAb IgG2 isotype control antibody, anti-trinitrophenol	Bio X Cell	Cat# BE0089
InVivoMAb IgG2 anti-mouse Ly6G antibody	Bio X Cell	Cat# BE0075-1
Anti-mouse IgG antibody	R&D Systems	Cat# AF007
Anti-mouse Mouse G-CSF antibody	R&D Systems	Cat# MAB414
Mouse-anti-human/mouse Ki-67 antibody	Biolegend	Cat# 350507
**Oligonucleotides and other sequence-based reagents**		
PCR primers	This study	Table EV1
**Chemicals, Enzymes and other reagents**		
Hair removing cream	Veet	Cat# PM8220626
Vaseline cream (100% White Petrolatum)	Unilever	Cat# 521234500
Beselna Cream 5% (Imiquimod)	Mochida Pharmaceutical Co., Ltd.	Cat# MO651
KINERET (Anakinra)	Biovitrum	Cat# 49163-54388
AmbionTM Nuclease-Free Water	Invitrogen	Cat# AM9937
Hanks’ Balanced Salt Solution 10x	Sigma	Cat# H4641-500ML
RPMI-1640 with L-Glutamine and Phenol Red	Wako	Cat# 189-02025
Ethanol 99.5%	Fujifilm	Cat# 052-03343
LiberaseTM	Roche	Cat# 5401127001
DNAse1	Roche	Cat# 10104159001
DNAse I Amplification grade	Invitrogen	Cat# 10977015
1 mol/L-HEPES Buffer solution	Nacalai Tesque	Cat# 17557-94
Mayer’s Hematoxylin solution	Wako	Cat# 131-09665
Eosin Y (Sodium Tetrabromofluorescein)	Fujifilm	Cat# 058-00062
Xylene	Fujifilm	Cat# 241-00091
Paraformaldehyde	Fujifilm	Cat# 1613-20145
Entellan TM	Sigma	Cat# 1.07961.0100
Bovine Serum Albumin	Merck	Cat# A9418-5G
RNEasy Mini Kit	Qiagen	Cat# 74104
THUNDERBIRD SYBR qPCR/RT set	Toyobo	Cat# QPS201
Primescript RT Master Mix	Takara Bio	Cat# RR036A
Hoechst 33342, trihydrochloride	Life Technologies	Cat# H3570
Propidium Iodide	Sigma	Cat# P4170-10MG
LPS-EB Ultrapure	Invivogen	Cat# tlrl-3pelps
Imiquimod	Sigma	Cat# I5159-200MG
Gelatin from cold fish skin	Merck	Cat# G7041-100G
DMEM High Glucose	Wako	Cat# 044-29765
CultureSure DMSO	Wako	Cat# 031-24051
Non-Essential Amino Acids Solution	Gibco	Cat# 11140050
GlutaMAX™ Supplement	Gibco	Cat# 35050-061
Penicillin-Streptomycin	Wako	Cat# 164-25251
Fetal Bovine Serum	Nichirei	Cat# 174012-500 ML
Mouse G-CSF ELISA Kit	Proteintech	Cat# KE10025
LEGENDplex™ Inflammation Panel (13-plex) with Filter Plate	Biolegend	Cat# 740150
NP40	Thermo Fischer	Cat# 28324
RNAsein plus	Promega	Cat# N2611
Real Time Cell Lysis buffer	Roche	Cat# 06366821001
5x RT Buffer	Takara	Cat# RR037A
ERCC RNA Spike-In Mix	Thermo	Cat# 4456740
20x enzyme mix	Takara	Cat# RR037A
T4 gene 32 protein	NEB	Cat# M0300L
Oligo(dT) 18 primer	Thermo	Cat# SO131
1^st^ NSR primer mouse	SIGMA	
2^nd^ NSR primer mouse	SIGMA	
NEB buffer 2	NEB	Cat# M0212L
Klenow fragment (3’-5’ exo-)	NEB	Cat# M0212L
dNTPs	Takara	Cat# 4030
AMPure XP beads	Beckman Coulter	Cat# A63881
Tagment DNA buffer	Illumina	Cat# FC-131-1096
Index primers	Illumina	Cat# FC-131-2001
High Sensitivity D5000 Screen Tape	Agilent	Cat# 5067-5592
GenNext® NGS Library Quantification kit	Toyobo	Cat# NLQ-101
Fixation/Permeabilization Concentrate	eBioscience	Cat# 00-5123-43
Fixation/Permeabilization Diluent	eBioscience	Cat# 00-5223-56
Permeabilization Buffer (10x)	eBioscience	Cat# 00-8333-56
Dihydrorhodhamine 123	Sigma	D1054-2MG
**Software**		
Imaris	Oxford Instruments	RRID: SCR_007370
Flowjo 10.10.0	BD	RRID: SCR_008520
GraphPad Prism 10.1.2 (324)	Dotmatics	RRID: SCR_002798
ImageJ 1.53t	NIH	RRID: SCR_003070
R Studio build 394	R	RRID: SCR_000432
**Other**		
Leica Cryostat CM1950	Leica	
BD Cell analyzer Canto II	BD	
BD Cell analyzer Symphony	BD	
BD Cell sorter Aria IIIu	BD	
All-in-One Fluorescence Microscope BZ-X800	Keyence	
Human RNA-seq data (Journal of Investigative Dermatology)	PMID: 24441097	https://www.ncbi.nlm.nih.gov/geo/query/acc.cgi?acc=GSE54456
Human RNA-seq data (Nature Communications)	PMID: 37308489	https://www.ncbi.nlm.nih.gov/geo/query/acc.cgi?acc=GSE173706
RNA-seq data of mouse skin neutrophils	PRJDB37718	https://ddbj.nig.ac.jp/search
RNA-seq data of mouse peripheral blood neutrophils	PRJDB40643	https://ddbj.nig.ac.jp/search

### Mice

C57BL/6 (CD45.2) founder mice were purchased from Japan SLC, Inc. and bred/maintained at the Center for Animal Resources and Development at Kumamoto University. Ly6G^Cre^; Rosa26^tdTom^ (CD45.2) (Hasenberg et al, 2015) mice were purchased from The Jackson Laboratory. All the animals were maintained in SPF condition with bedding, environmental enrichment, and were provided ad libitum access to standard chow diet and water. The animal facility was maintained under controlled temperature and humidity with 12 h light/dark cycle. All animal experiments were performed in female mice age 8–12-week otherwise excluded. Although blinding procedure was not applied in this study, to minimize subjective bias, animals were randomized twice, first at weaning, second before conducting experiment to minimize subjective bias, they were rehoused into experimental cages so that each group included mice from at least two litters, without selective assignment. All the mice experiments followed the approved guideline of Animal Care and Use Committee of Kumamoto University (approval number: A2025071R1).

### Treatment

Psoriasis was induced by daily topical application of 62.5 mg Imiquimod (IMQ) cream (Beselna, Mochida Pharmaceutical) to the pre-shaved and hair-removed 2.5 × 2.5 cm dorsal skin for four consecutive days. Anakinra (KINERET, Biovitrium) (37 μg) was intraperitoneally (*i.p*.) administered daily starting at 1 day before psoriasis induction (−1 d) till 1 day before mice analysis (3 d). Neutrophils were depleted by daily *i.p*. administration of 50 μg anti-Ly6G (1A8, BioXCell) between −2 d and 3 d. The same dose of IgG-isotype (2A3, BioXCell) was given to control mice following the same regimen. Anti-G-CSF (MAB414, R&D Systems) or IgG-isotype control antibody (AF007, R&D Systems) (10–20 μg) was intraperitoneally (*i.p*.) or intradermally (*i.d*.) injected daily from 0 d to 3 d 30 min prior to IMQ topical association. For intradermal injection, 4 symmetrically distanced dorsal skin was injected with 2.5 µg in 50 µL antibody per site per day.

### Clinical and histological analysis

Psoriasis clinical score (erythema, scaling) was measured according to the previous studies (van der Fits et al, 2009; Li et al, 2021). In short, the skin was observed and clinically scored in a scale representing none (0), mild (1), moderate (2), significant (3), and severe (4). Histological analysis of dorsal skin was performed by embedding dorsal skin in O.C.T compound (Sakura Finetek) and cryosectioned with 10 µm thickness. Sections were dried in room temperature for 1 h and subsequently fixed with 4% PFA (Fujifilm) for 10 min. Fixed sections were then stained with Hematoxylin (Wako) and Eosin (Fujifilm) for 20 min and 5 min, respectively. After staining, the sections were dehydrated stepwisely in Ethanol (Wako) soaking (70% 15 s, 90% 90 s, and 100% 5 min) and Xylene (Fujifilm) for 15 min. Dehydrated sections were mounted with Entellan (Sigma), and scanned with light microscope (EVOS M5000, ThermoFisher Scientific), followed by analysis on Image J 1.53t (NIH).

### Cytokines measurement

The peripheral blood was harvested retro-orbitally using untreated glass capillary (Hirschmann) and sat at RT for 30 min until clotted. Subsequently, the serum was optimally separated by centrifuging (2000 × *g*; 10 min; 4 °C), collected, and snap frozen in liquid nitrogen before storage at −80 °C till analysis. G-CSF and other cytokines in the serum were measured using Mouse G-CSF ELISA kit (Proteintech) and flowcytometry-based multi-cytokine measurement Legendplex^TM^ Mouse Inflammation Panel (Biolegend), respectively, according to the manufacture’s instruction.

### Flowcytometry analysis

To prevent contamination of circulating cells into tissue, intracardiac PBS perfusion was conducted before tissue collection. Immediately after the procedure, 4 cm^2^ skin was harvested and subsequently cleared from the remnant hair and dead skin (scale) gently with scalpel. The resultant skin was minced in a digestion buffer containing 62.5 µg/mL Liberase^TM^ (Roche), 50 µg/mL DNAse (Roche), 10 mM HEPES (Nacalai Tesque) in RPMI-1640 medium (Wako), followed by incubation with gentle rocking at 37 *°*C for 2 h. An equal volume of pre-warmed 0.1% BSA/HBSS (Merck/Sigma) was added to the mixture and mechanical tissue homogenization was performed using Gentle MACS^TM^ (program C_01) to prepare a homogeneous single-cell suspension. Subsequently, the skin homogenate was filtered stepwise with 100 μm, 70 µm, and 40 µm strainer (BD Biosciences), and centrifuged at 400 × *g* for 5 min 4 *°*C to harvest skin cells in a pellet and instantly resuspend in 2% FBS 0.2 mM EDTA in PBS (FACS buffer). To reduce non-specific immunostaining, Fcg blocking was performed by incubating the cell suspension with CD16/32 (93) for 15 min in RT prior to antibody-staining with fluorophore-conjugated antibodies (30 min on ice) against the following markers (clone name) (dilution): CD45.2 (104) (1:50), CD19 (6D5) (1:100), CD11b (M1/70) (1:100), Ly6C (HK1.4) (1:500), Ly6G (1A8) (1:200), CD31 (390) (1:100), Ter119 (TER-119) (1:100), CD45 (30-F11) (1:50), CD49f (GoH3) (1:100), CD207 (4C7) (1:100), CD101 (Moushi101) (1:200), CD11c (N418) (1:100), Sca-1 (D7) (1:100), and CD34 (RAM34) (1:50).

For BM cells, cells were collected by grinding two hindleg bones (tibiae and femur) using a mortar and pestle in FACS buffer before treatment with ammonium-chloride-potassium buffer (0.15 mM NH_4_Cl, 1.0 mM KHCO_3_, 0.1 mM Na_2_EDTA) to remove red blood cells. The BM single-cell suspension was preincubated with CD16/32 (93) FcγR blocking antibody in RT for 15 min, prior to staining with the fluorophore conjugated antibodies/reagents against (clone name) (dilution): CD45 (30-F11) (1:50), CD11b (M1/70) (1:100), Ly6G (1A8) (1:200), IL7ra (A7R34) (1:50), c-Kit (2B8) (1:100), Sca-1 (D7) (1:100), CD16/32 (93) (1:100), CD34 (RAM34) (1:50), CD48 (HM48-1) (1:100), CD150 (TC15-12F.2) (1:100), CD135 (A2F10) (1:100), and streptavidin-conjugated antibody (1:200) for 30-60 min on ice. For HSPC analysis, additional staining with biotinylated-antibodies against Ter119 (TER-119) (1:500), CD3 (145-2C11) (1:500), CD4 (GK1.5) (1:500), CD8 (53-6.7) (1:500), NK1.1 (PK136) (1:500), B220 (RA3-6B2) (1:500), CD11b (M1/70) (1:500), Gr-1 (RB6-8C5) (1:500) was performed before fluorophore-antibodies association.

For all FACS analysis, dead cells were stained with Hoechst33342 (1:5000) or Propidium Iodide (1:5000) shortly before analysis, and analysis was done within 4 h after. Samples were analyzed on FACS Symphony A3 or FACS Aria IIIu (BD Biosciences). All the data were further analyzed using Flowjo10.10.0.

### Quantitative RT-PCR

For tissues, 50 mg of the snap frozen sample was pulverized using an ultra-cold conditioned (liquid nitrogen soaked) metal mortar and pestle (Tokken, inc.). RNA was isolated from the tissue homogenate with RNeasy Mini Kit (Qiagen) and reverse transcribed into cDNAs with reverse transcriptase containing reagents mix (Takara). The resultant cDNA was subjected to RT-PCR with the respective primers (Table EV1), and the relative expression of gene of interest was quantified relative to housekeeping genes (*Gapdh*).

For cells, 100 cells were FACS-sorted into the designated 96-well plate containing cell lysis buffer and subjected to cDNA synthesis according to the detailed protocol described in the previous study adapted for 100 cells processing, followed by RT-PCR (Sezaki et al, 2022).

### Cellular ROS measurement

Single-cell suspensions were prepared from skin, PB, and BM, and resuspended in chilled 2% FBS containing PBS. After cell surface markers were stained, the cells were incubated with 10 µg/mL Dihydrorhodamine 123 (Sigma) on ice for 30 min and washed with cold PBS for flow cytometric analysis.

### Cell cycle analysis

The whole BM cells were stained for their surface markers to define phenotypic HSC and MPP, and washed with 1x Permeabilization Buffer (eBioscience). Cells were then permeabilized with Fixation/Permeabilization Concentrate (eBioscience) for 30 min at R.T., washed, and incubated with anti-Ki67 antibody in Permeabilization Buffer for another 30 min on ice until being washed and resuspended in chilled Permeabilization Buffer containing Propidium Iodide before FACS analysis.

### Transplantation assay

Seven hundred Lin^-^Sca-1^+^c-Kit^+^ (LSK) cells isolated from CD45.1^+^ donor BM were transplanted together with CD45.1/2^+^ 3 × 10^5^ competitor whole BM cells into lethally (9.0 Gy)-irradiated CD45.2^+^ recipient mice. After transplantation, peripheral blood was collected serially at 2, 3, 4, 6, and 8 weeks post-transplantation for assessing donor chimerism and lineage distribution.

### In vitro culture of skin-derived endothelial cell

Dorsal skin from healthy mice (strain, sex, and age are mentioned above) were harvested and digested into single-cell suspension in prior to fluorophore conjugated antibody staining for immunophenotypic endothelial cell (CD45^-^ Ter119^-^ CD31^+^ Sca-1^+^). Sixty thousand endothelial cells were sorted into a flat-bottom 96-well plate pre-coated with 0.2% gelatin. The sorted cells were recovered in 50% FBS, 100 unit/mL Penicillin G, 100 µg/mL Streptomycin (Wako), 2 mM Glutamax (Gibco), and 1x NEAA (Gibco) in DMEM (all Thermo Fisher Scientific) at 37* °*C, 5% O_2_ for 36 h. Subsequently, the media is substituted with stimulating media containing 20% FBS, 100 unit/mL Penicillin G, 100 µg/mL Streptomycin (Wako), 2 mM Glutamax (Gibco), and 1x NEAA (Gibco) with 5% DMSO (Wako) as a control or 50 µg/mL IMQ (Sigma) in DMEM at 37 *°*C, 5% O_2_ for another 48 h. Finally, media was harvested for G-CSF measurement by ELISA (Proteintech).

### Three-dimensional (3D) intra-vital imaging

Mice were anesthetized with Zoletil (30 mg/kg, Virbac Korea) and Xylazine (10 mg/kg, Elanco). Hair of the dorsal skin was gently removed using a depilatory cream (Drammock) and cleaned with distilled water. To visualize blood vessel, 25 μg anti-CD31 antibody (553708, BD Pharmingen) conjugated with SeTau 647 (K9-4149, SETA Biomedicals) was intravenously injected at 30 min before the intravital imaging with commercial intravital confocal and two-photon microscopy system (IVM-CMS3, IVIM Technology). Using a femto-second pulsed laser (920 nm, Alcor, Spark Lasers) and a high NA 25X water-immersion objective lenses (CFI75 Apo LWD 25XC W, MRD77225, Nikon), two-photon Z-stack images with 3-μm interval were captured from the dorsal skin of the anesthetized mouse. Second harmonic generation (SHG) signal from dermal collagen, red fluorescence (Ly6G-tdTomato), and far-red fluorescence (pseudo-colored in green, CD31-SeTau 647) were collected through emission filters with bandpass wavelengths of 459–464 nm, 574–626 nm, and 642–709 nm, respectively. Of note, the elongated structures detection resembling hair bulge were autofluorescent signals which were removed from further analysis. The blood vessels (CD31^+^) were transformed into Surface, and neutrophils (Ly6G^+^) were transformed into Spot. The distribution of Spot at >0–10, >10–20, >20–30, >30–40, >40–50, and >50 µm distance to the Surface was quantified. At the same time, random Spot were generated using a Python-based module tifffile ver. 2025.3.30 to create 3D tiff files containing randomly distributed spheric voxels with x/y/z of 2.5 µm which was transformed into Random Spot and overlaid into the original 3D imaging file. The amount of Random Spot per stacked files is equal to the number of phenotypic neutrophils per 3D image and follow the same distance measurement to surface as was done with neutrophil Spots. Image processing and analysis was done in Imaris 8 (Oxford Instruments).

### RNA sequencing

One thousand neutrophils (CD45^+ ^CD11b^+ ^Ly6C^mid ^Ly6G^+^) were FACS-sorted from dorsal skin as well as peripheral blood, and processed to RAMDA-seq(Hayashi et al, 2018). In short, the cells are disintegrated using lysis-buffer (Roche) and the whole RNAs were reverse-transcribed into first-strand cDNAs (Takara Bio Inc.). Using Klenow Fragments (3’-5’ exo; New England Biolabs), the double-strand cDNAs were created which were magnetically purified with AMPure XP beads (Beckman Coulter) and further processed using Nextera XT DNA sample Prep kit to generate a library (Illumina). The quality and quantity of the library was measured with Agilent 4150 TapeStation (Agilent Technologies, Santa Clara, CA) and GenNext® NGS library quantification kit (Toyobo). Upon confirmation of adequate quality and quantity of the library, it was sequenced using NovaSeq X system (Illumina). Quality check and trimming of single-end sequences were completed with the trim_galore (version 0.4.3) package. Quality and length parameters were quality 30 and length 30. Filtered sequences were processed to remove mitochondrial mRNA with the SortMeRNA (version 2.1.1) and were aligned to mouse reference sequences (GRCm39) from (http://www.genecodesgenes.org/mouse/release.html) with ultrafast RNAseq aligner STAR (version 2.7.10b; cite Dobin et al, 2013). All the aligned bam files were used with mouse GFF annotation files (GRCm39) as input into the featureCounts program from the Subreads program (version 2.1.1) to count the raw reads for each gene and sample, and to create a gene count matrixDES. To calculate differentially expressed genes, the DESeq2 (version 1.30.1) package was used in R. The gene ontology (GO) analysis was performed in GSEA 4.4.0 software following default set parameters with customization including m5.all.v2025.Mm.symbols.gmt (https://www.gsea-msigdb.org/gsea/msigdb/mouse/genesets.jsp?collection=M5), Gene sets database reference, 1000 Number of permutations, gene_sets Permutation type, and Signal2Noise metric for ranking genes (Castanza et al, 2023; Subramanian et al, 2005). Gene expression levels were quantified using Z-scores, with positive values indicating upregulation and negative values indicating downregulation.

### Re-analysis of human psoriasis RNA-seq data

The gene-expression dataset GSE54456 (Li et al, 2014) is a skin bulk-RNA-seq data which has been normalized by reads per kilobase of exon per million mapped reads (RPKM) publicly available in Gene Expression Omnibus (GEO) repository. The data contains RNA expression level from 82 normal and 92 psoriatic human skin biopsies. The raw data from GEO was imported and incorporated to its metadata (sample group, methods, etc.) using GEOquery v.2.76.0 package. Subsequently, UMAP clustering was done using umap v.0.2.10.0 and plotly 4.11.0 packages. Those data processing was done in R-studio build 394. The expression value of the gene of interest was extracted to create customized bar charts in Prism version 10.

The GSE173706 single-cell RNA-seq dataset was obtained from the GEO public repository (Ma et al, 2023). Each sample was filtered to remove cells with >5% mitochondrial content and potential doublets (2% of total cells). Filtered samples were then integrated, and expression counts were log-transformed to 10^4^ per cell. Clustering analysis was done in scvi-tools v.1.2.0 and was used to identify cellular clusters which were annotated based on the original study (Ma et al, 2023). Gene expression level mapping and cell type frequencies were calculated using scanpy v.1.10.3.

### Statistical analysis and visualization

Statistical test was performed with adequate replicate of *n* ≥ 3 per group and no single data nor outliers were excluded from the analysis. The Shapiro-Wilk test was performed to assess the data distribution, identifying it as normal (*p* ≥ 0.05) or skewed (*p* < 0.05). For dataset containing 2 groups, either an unpaired T-test (normal distribution) or a Mann–Whitney (skewed distribution) statistical test was performed. For dataset containing more than 2 groups, one-way ANOVA (normal distribution) or Kruskal–Wallis (skewed distribution) with Dunnet or Dunn test for its multiple group comparison was done. Prism version 10 was used to generate graphs and to perform statistical analysis in this study with the star count annotation reflecting significance level as: *p < 0.05; **p < 0.01; ***p < 0.001; ****p < 0.0001.

### Graphics

Graphics were created in Biorender.com or Adobe Illustrator.

## Supplementary information

Table EV1

Peer Review File

Source data Fig. 1

Source data Fig. 2

Source data Fig. 3

Source data Fig. 4

Source data Fig. 5

Source data Fig. 6

Expanded View Figures

## Data Availability

The raw RNA-seq files have been deposited to DDBJ BioProject database (https://ddbj.nig.ac.jp/search) with accession number PRJDB37718 for skin-neutrophils and PRJDB40643 for PB-neutrophils. The source data of this paper are collected in the following database record: biostudies:S-SCDT-10_1038-S44321-026-00456-y.
