# Supplementary figures and images for: Skin-derived G-CSF activates pathological granulopoiesis upon psoriasis
Source: EMBO Mol Med. 2026 Jun 16;18(7):2777–807. doi: 10.1038/s44321-026-00456-y (PMC13365241; doi:10.1038/s44321-026-00456-y)

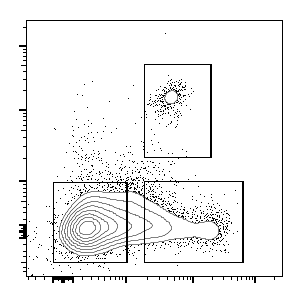

Supplement: Supplementary file 3 — Source data Fig. 1 [file 44321_2026_456_MOESM3_ESM.zip › 1C/Fig1C_Vas_left.tiff]

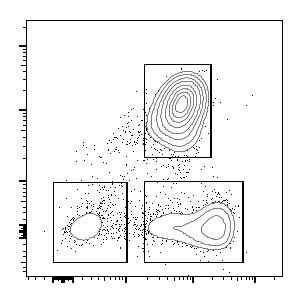

Supplement: Supplementary file 3 — Source data Fig. 1 [file 44321_2026_456_MOESM3_ESM.zip › 1C/Fig1C_IMQ_right.tiff]

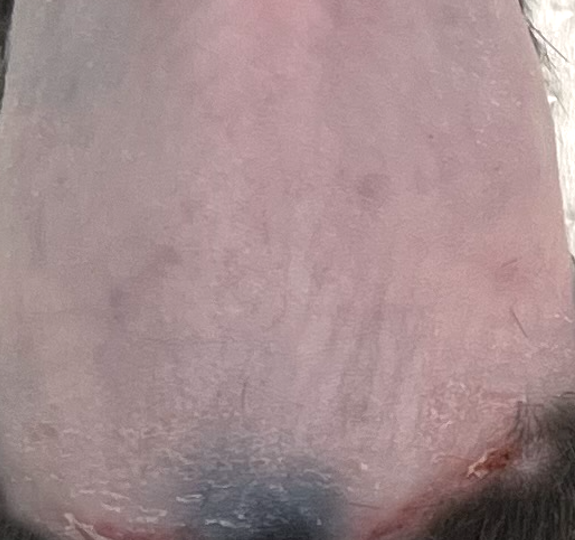

Supplement: Supplementary file 3 — Source data Fig. 1 [file 44321_2026_456_MOESM3_ESM.zip › 1B/FIg1B_Vas_upper.png]

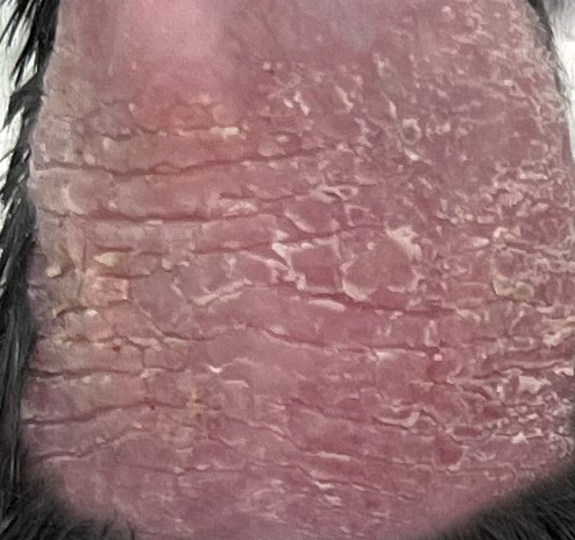

Supplement: Supplementary file 3 — Source data Fig. 1 [file 44321_2026_456_MOESM3_ESM.zip › 1B/FIg1B_IMQ_upper.png]

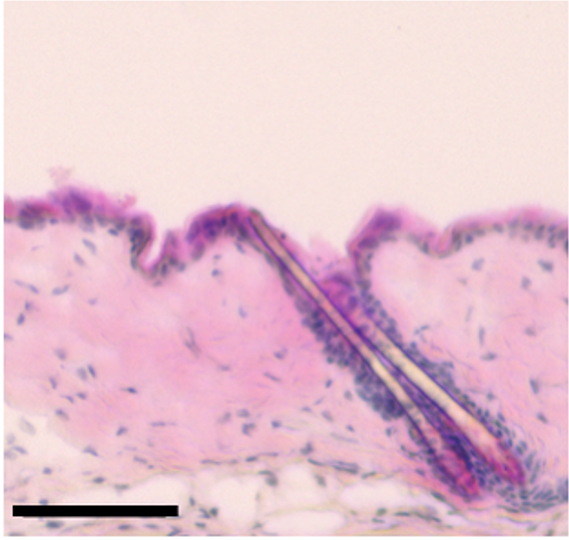

Supplement: Supplementary file 3 — Source data Fig. 1 [file 44321_2026_456_MOESM3_ESM.zip › 1B/FIg1B_Vas_lower.png]

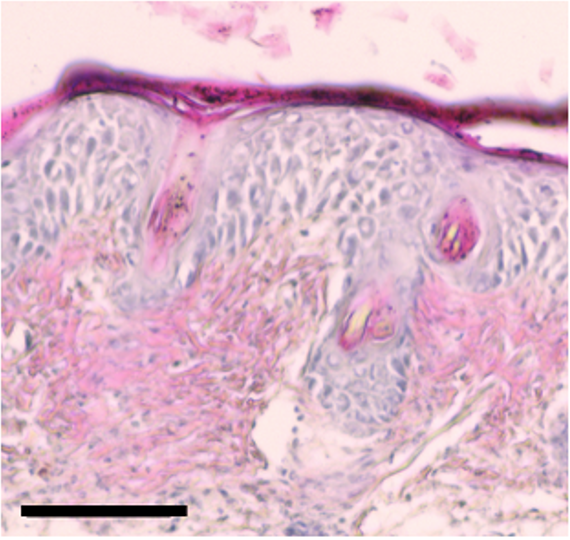

Supplement: Supplementary file 3 — Source data Fig. 1 [file 44321_2026_456_MOESM3_ESM.zip › 1B/FIg1B_IMQ_lower.png]

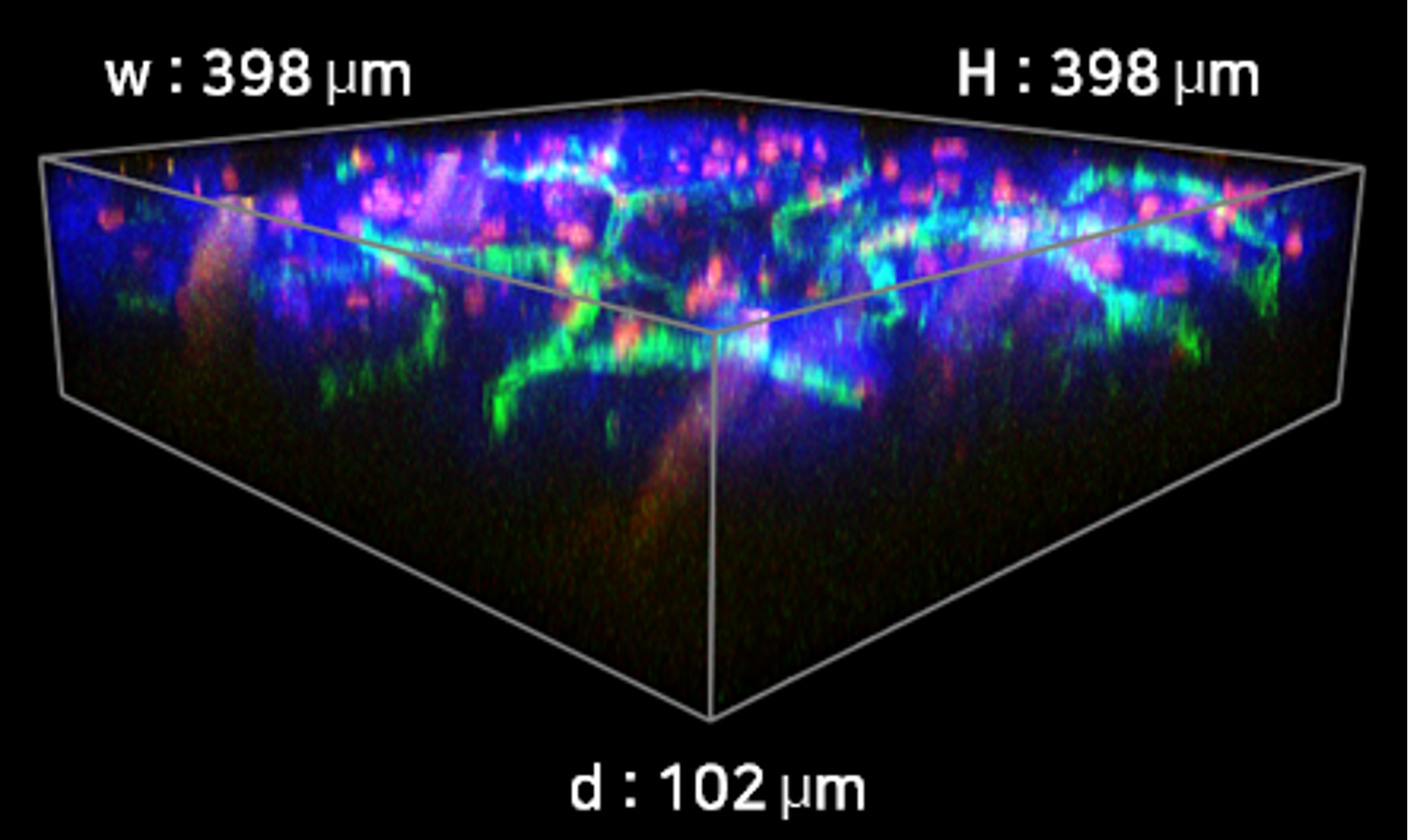

Supplement: Supplementary file 3 — Source data Fig. 1 [file 44321_2026_456_MOESM3_ESM.zip › 1E/Fig1E_IMQ_1d.png]

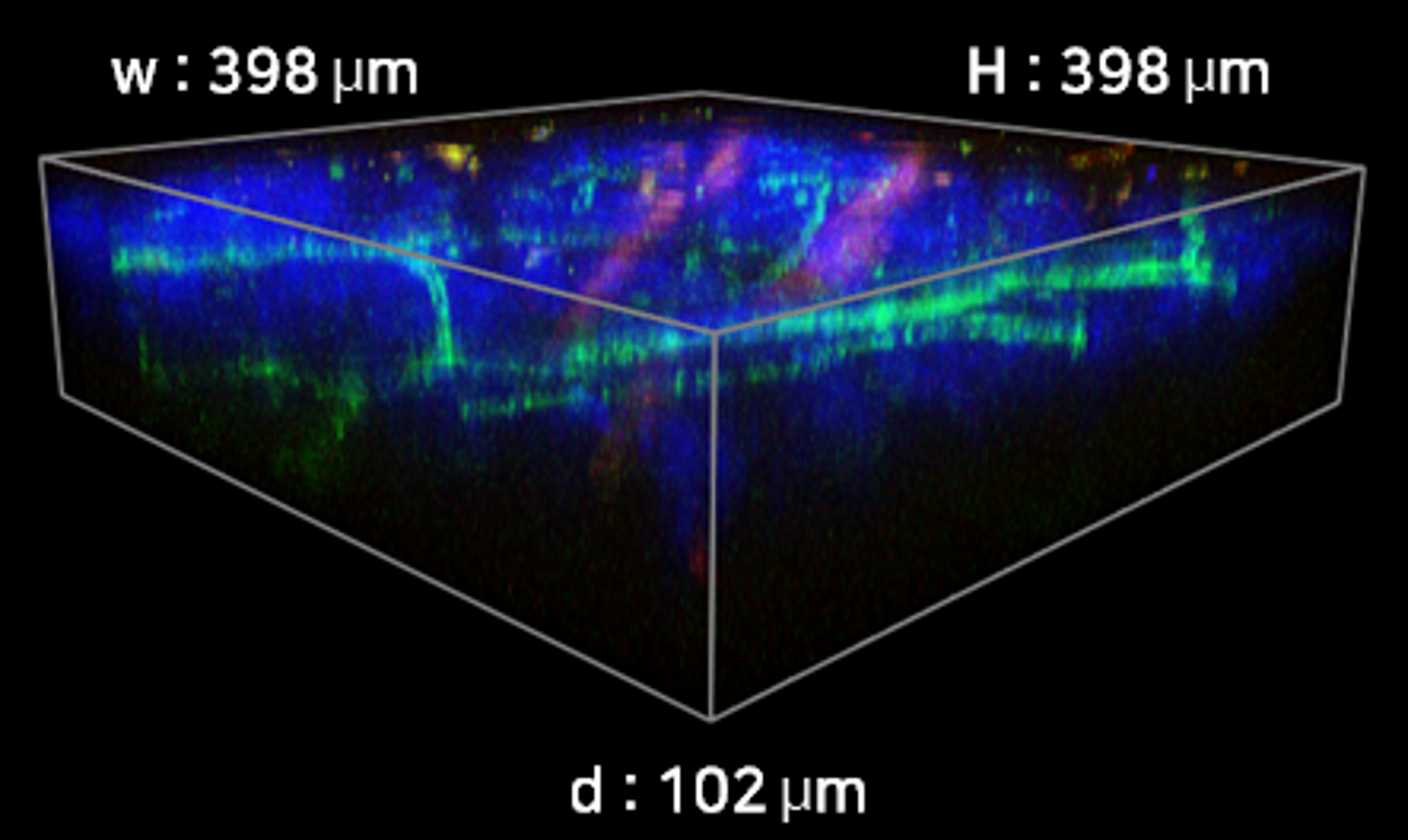

Supplement: Supplementary file 3 — Source data Fig. 1 [file 44321_2026_456_MOESM3_ESM.zip › 1E/Fig1E_Vas_2d.png]

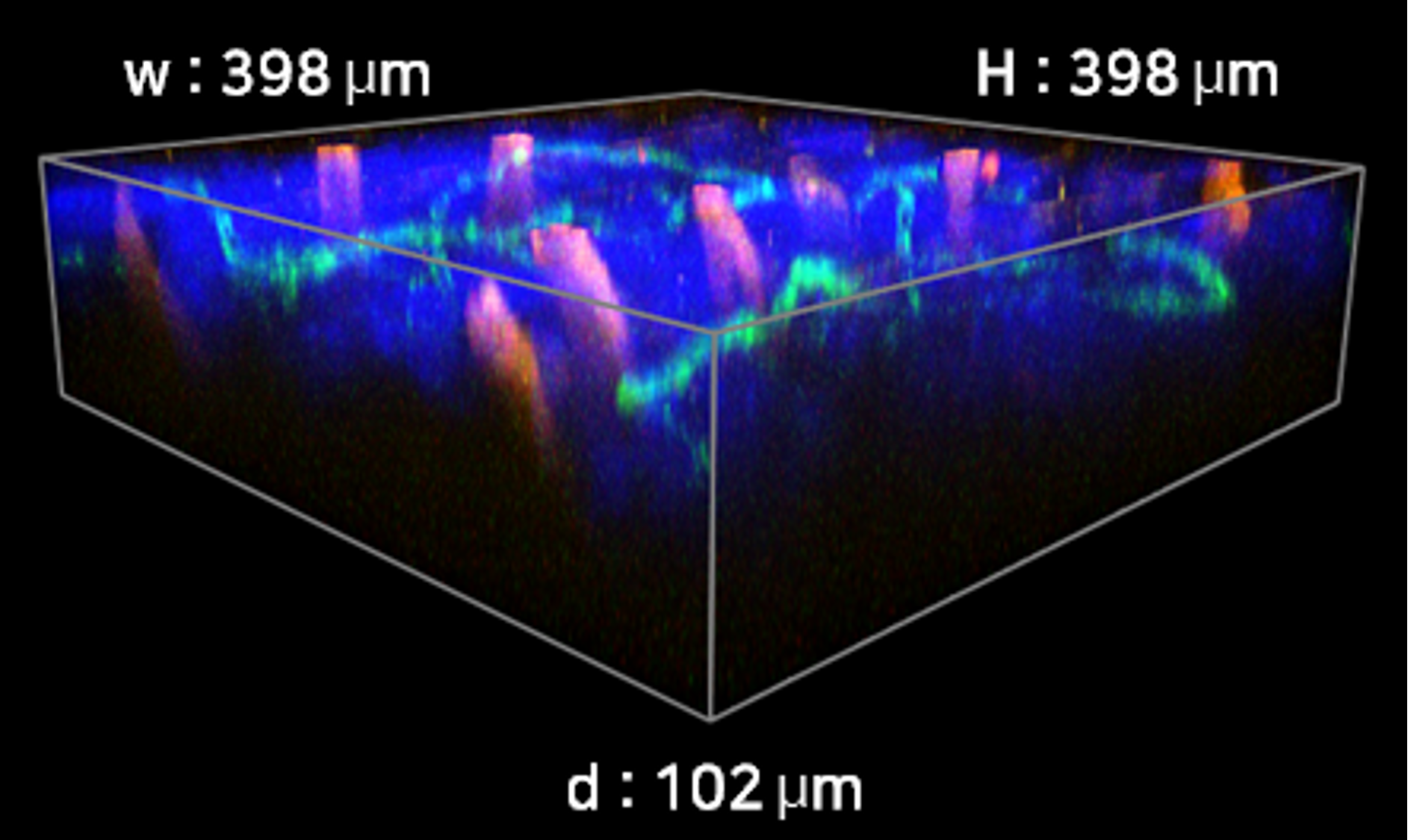

Supplement: Supplementary file 3 — Source data Fig. 1 [file 44321_2026_456_MOESM3_ESM.zip › 1E/Fig1E_IMQ_0d.png]

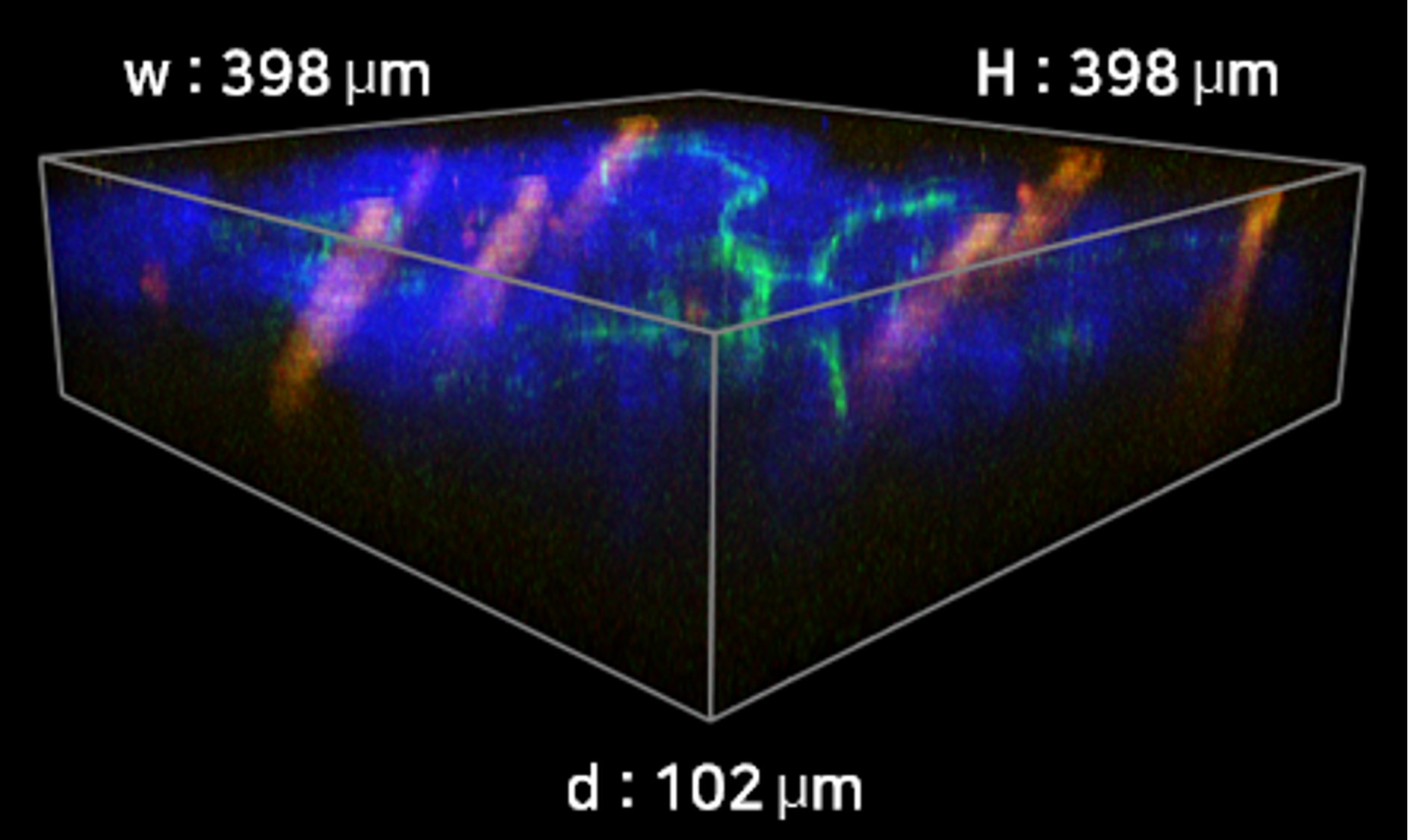

Supplement: Supplementary file 3 — Source data Fig. 1 [file 44321_2026_456_MOESM3_ESM.zip › 1E/Fig1E_Vas_0d.png]

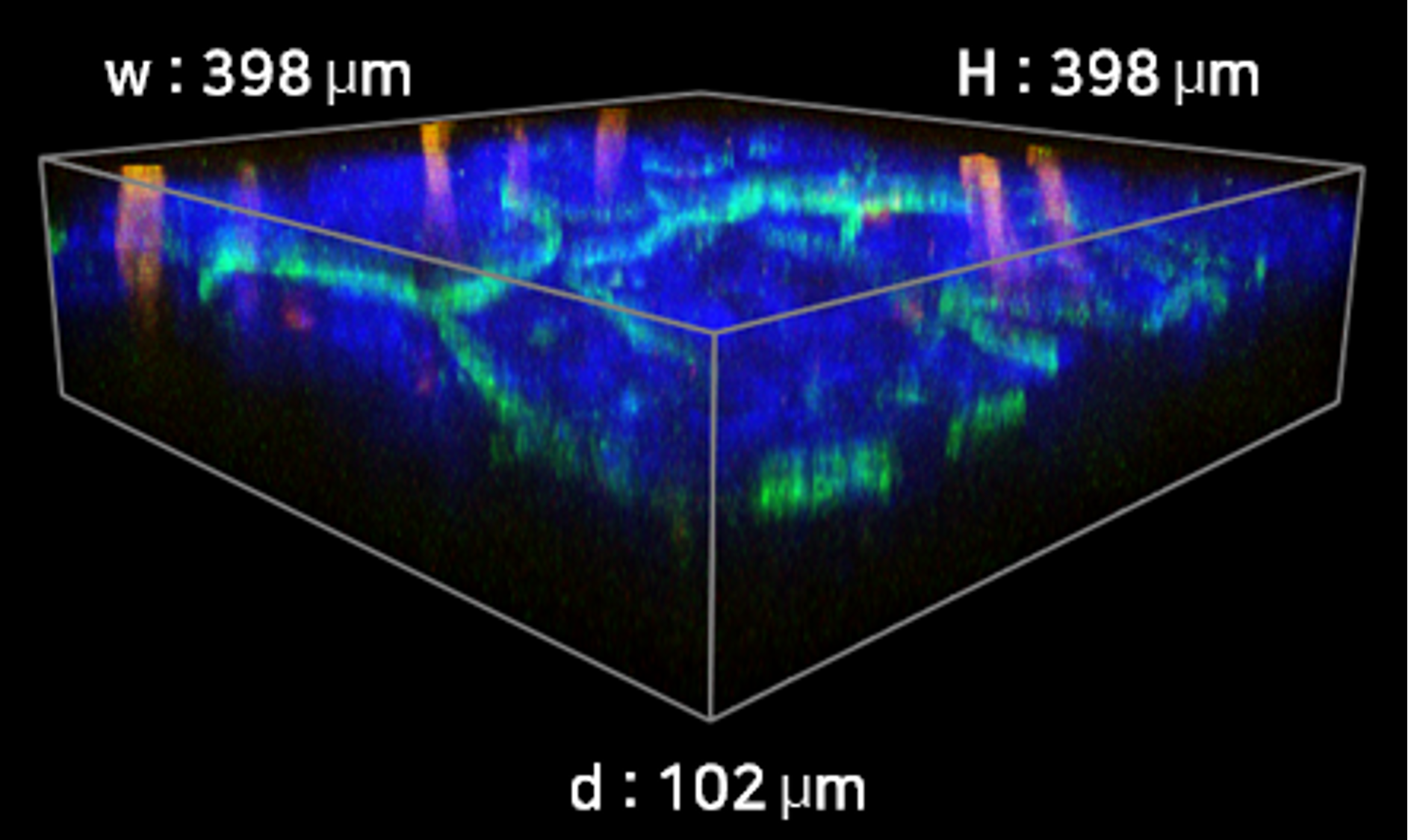

Supplement: Supplementary file 3 — Source data Fig. 1 [file 44321_2026_456_MOESM3_ESM.zip › 1E/Fig1E_Vas_1d.png]

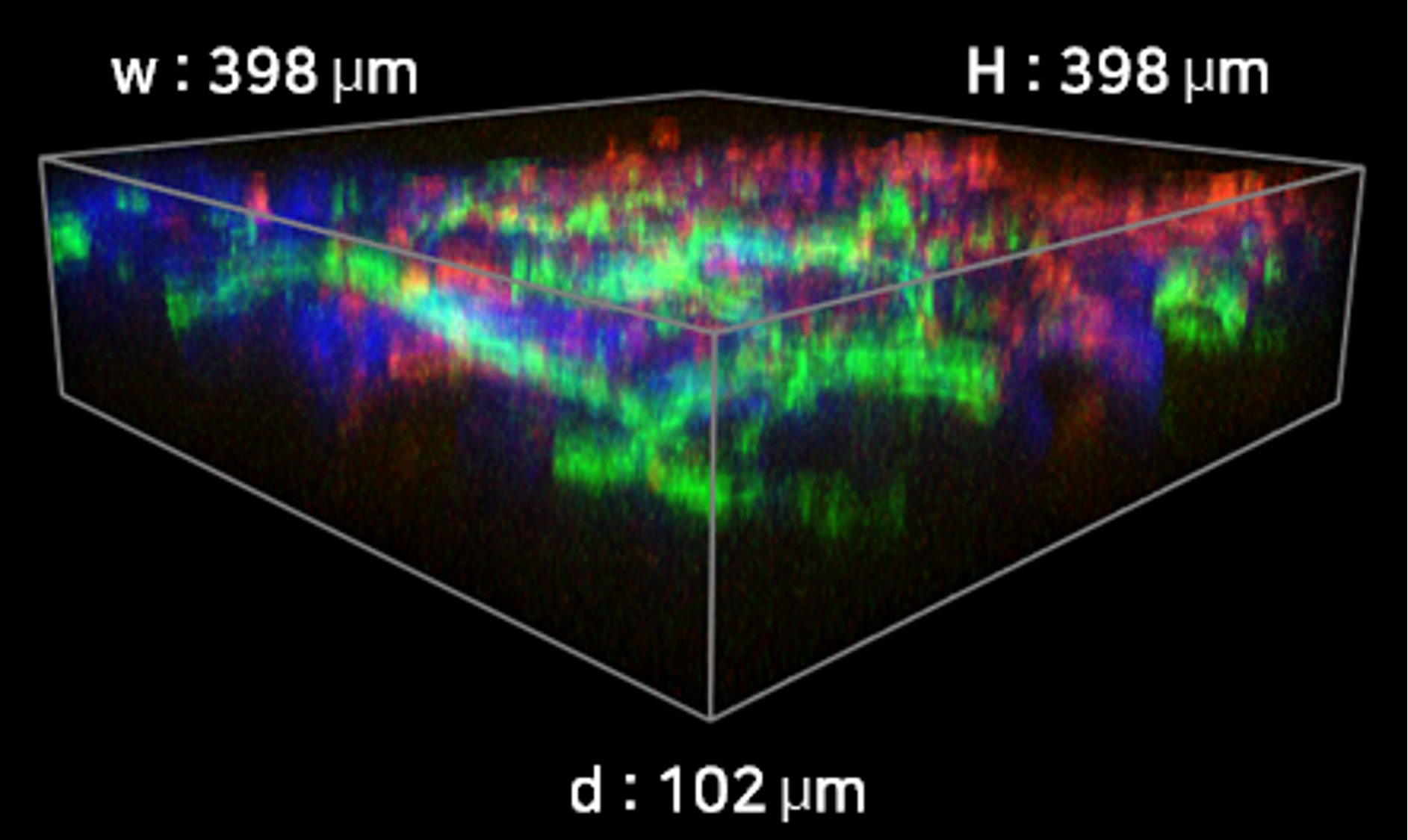

Supplement: Supplementary file 3 — Source data Fig. 1 [file 44321_2026_456_MOESM3_ESM.zip › 1E/Fig1E_IMQ_2d.png]

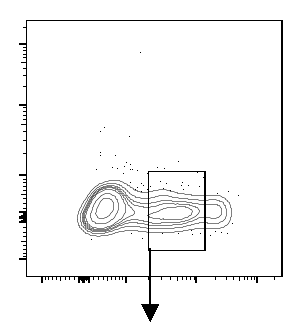

Supplement: Supplementary file 4 — Source data Fig. 2 [file 44321_2026_456_MOESM4_ESM.zip › 2F/Fig2F_aLy6G_upper_right.tiff]

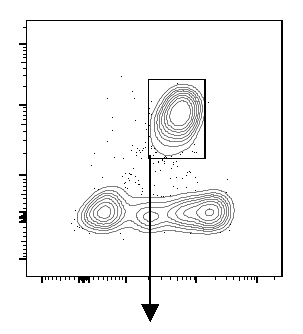

Supplement: Supplementary file 4 — Source data Fig. 2 [file 44321_2026_456_MOESM4_ESM.zip › 2F/Fig2F_IgG_upper_left.tiff]

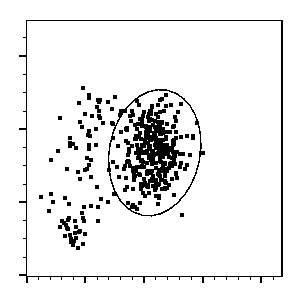

Supplement: Supplementary file 4 — Source data Fig. 2 [file 44321_2026_456_MOESM4_ESM.zip › 2F/Fig2F_IgG_lower_left.tiff]

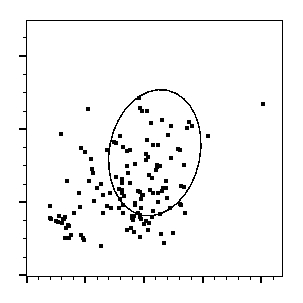

Supplement: Supplementary file 4 — Source data Fig. 2 [file 44321_2026_456_MOESM4_ESM.zip › 2F/Fig2F_aLy6G_lower_right.tiff]

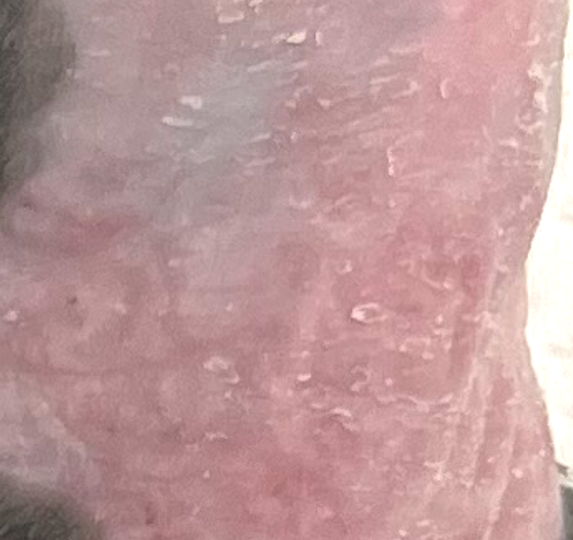

Supplement: Supplementary file 4 — Source data Fig. 2 [file 44321_2026_456_MOESM4_ESM.zip › 2H/Fig2H_antiLy6G_upper.png]

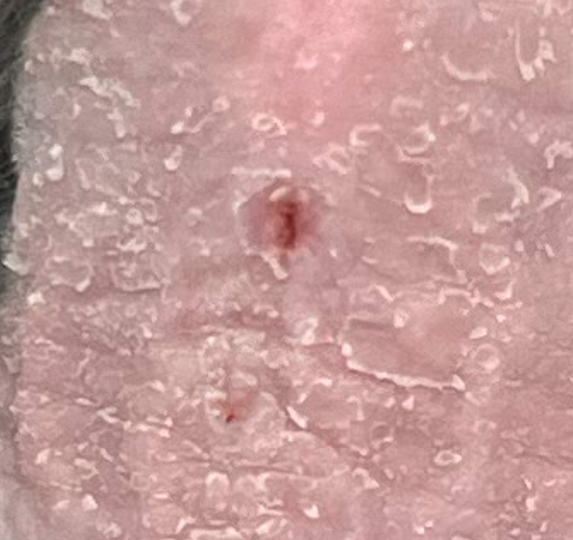

Supplement: Supplementary file 4 — Source data Fig. 2 [file 44321_2026_456_MOESM4_ESM.zip › 2H/Fig2H_IgG_upper.png]

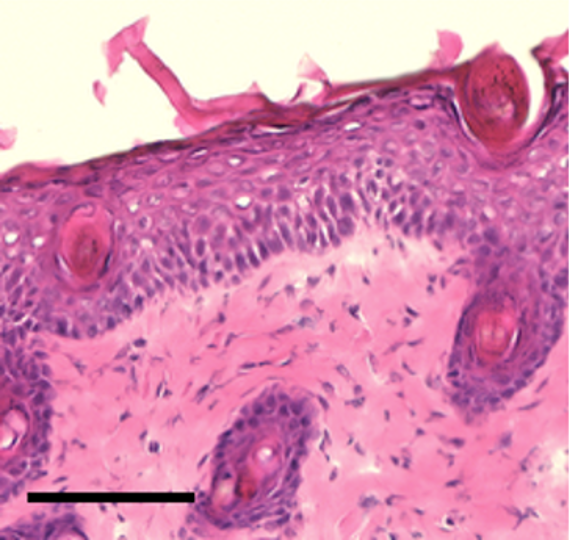

Supplement: Supplementary file 4 — Source data Fig. 2 [file 44321_2026_456_MOESM4_ESM.zip › 2H/Fig2H_IgG_lower.png]

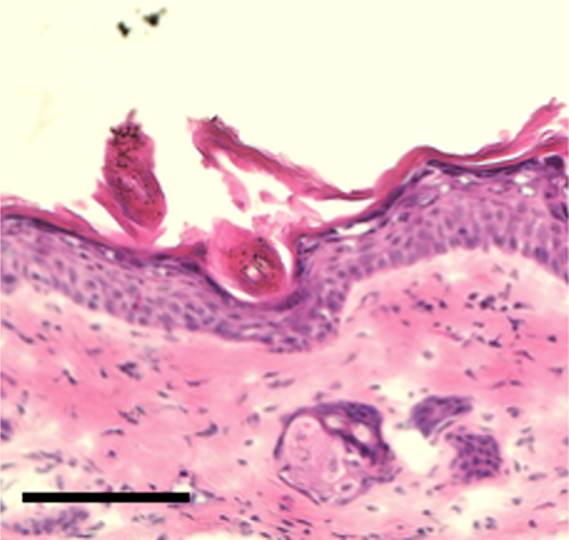

Supplement: Supplementary file 4 — Source data Fig. 2 [file 44321_2026_456_MOESM4_ESM.zip › 2H/Fig2H_antiLy6G_lower.png]

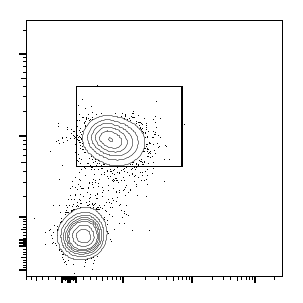

Supplement: Supplementary file 5 — Source data Fig. 3 [file 44321_2026_456_MOESM5_ESM.zip › 3E/Fig3E_GMP_IMQ4d.tiff]

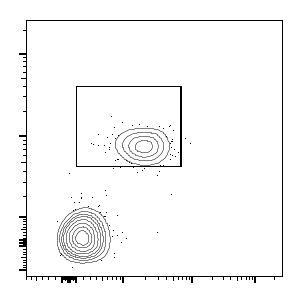

Supplement: Supplementary file 5 — Source data Fig. 3 [file 44321_2026_456_MOESM5_ESM.zip › 3E/Fig3E_GMP_IMQ2d.tiff]

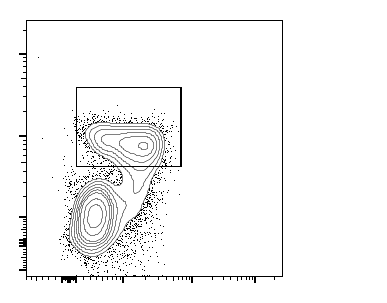

Supplement: Supplementary file 5 — Source data Fig. 3 [file 44321_2026_456_MOESM5_ESM.zip › 3E/Fig3E_GMP_Vas16h.tiff]

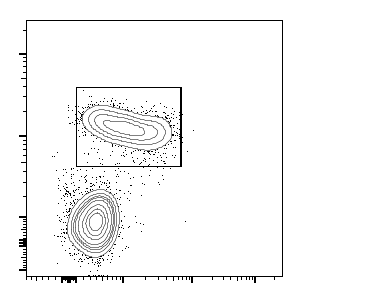

Supplement: Supplementary file 5 — Source data Fig. 3 [file 44321_2026_456_MOESM5_ESM.zip › 3E/Fig3E_GMP_IMQ16h.tiff]

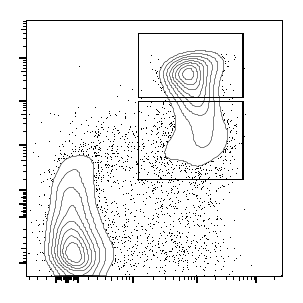

Supplement: Supplementary file 5 — Source data Fig. 3 [file 44321_2026_456_MOESM5_ESM.zip › 3F/FIg3F_IMQ2d.tiff]

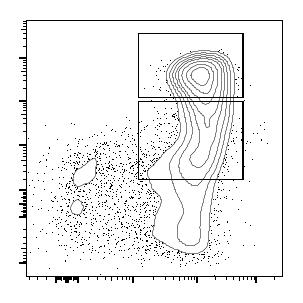

Supplement: Supplementary file 5 — Source data Fig. 3 [file 44321_2026_456_MOESM5_ESM.zip › 3F/FIg3F_IMQ4d.tiff]

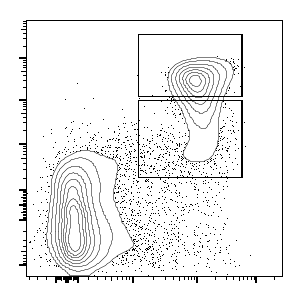

Supplement: Supplementary file 5 — Source data Fig. 3 [file 44321_2026_456_MOESM5_ESM.zip › 3F/FIg3F_IMQ16h.tiff]

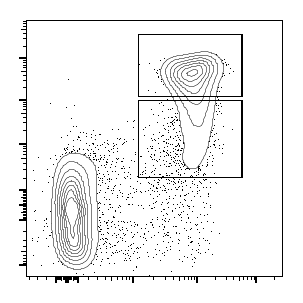

Supplement: Supplementary file 5 — Source data Fig. 3 [file 44321_2026_456_MOESM5_ESM.zip › 3F/FIg3F_Vas16h.tiff]

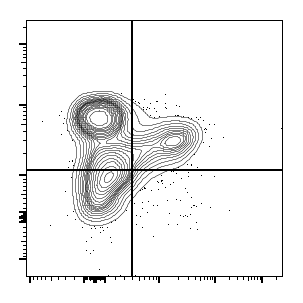

Supplement: Supplementary file 5 — Source data Fig. 3 [file 44321_2026_456_MOESM5_ESM.zip › 3B/Fig3B_IMQ16h_upper.tiff]

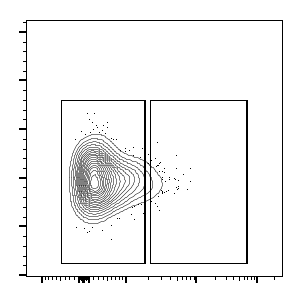

Supplement: Supplementary file 5 — Source data Fig. 3 [file 44321_2026_456_MOESM5_ESM.zip › 3B/Fig3B_IMQ2d_lower.tiff]

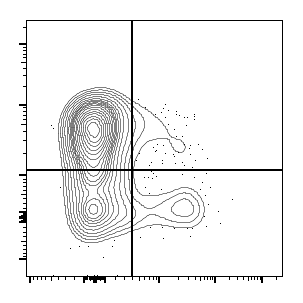

Supplement: Supplementary file 5 — Source data Fig. 3 [file 44321_2026_456_MOESM5_ESM.zip › 3B/Fig3B_Vas16h_upper.tiff]

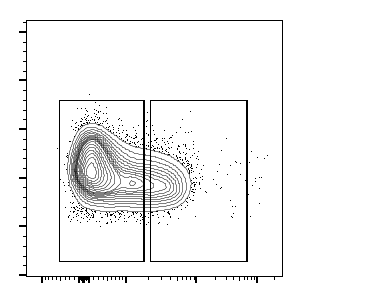

Supplement: Supplementary file 5 — Source data Fig. 3 [file 44321_2026_456_MOESM5_ESM.zip › 3B/Fig3B_IMQ4d_lower.tiff]

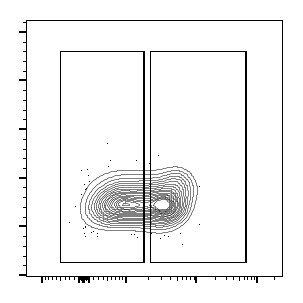

Supplement: Supplementary file 5 — Source data Fig. 3 [file 44321_2026_456_MOESM5_ESM.zip › 3B/Fig3B_Vas16h_lower.tiff]

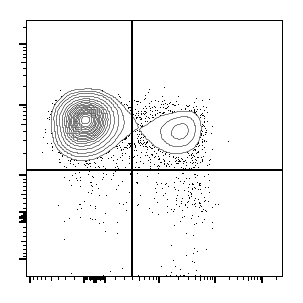

Supplement: Supplementary file 5 — Source data Fig. 3 [file 44321_2026_456_MOESM5_ESM.zip › 3B/Fig3B_IMQ4d_upper.tiff]

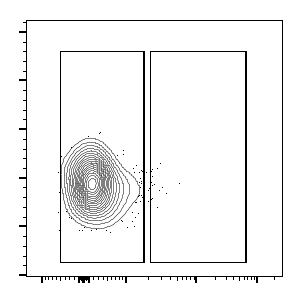

Supplement: Supplementary file 5 — Source data Fig. 3 [file 44321_2026_456_MOESM5_ESM.zip › 3B/Fig3B_IMQ16h_lower.tiff]

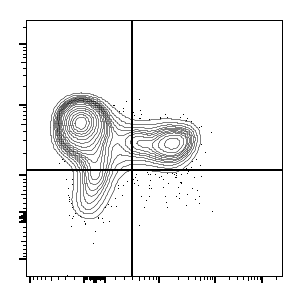

Supplement: Supplementary file 5 — Source data Fig. 3 [file 44321_2026_456_MOESM5_ESM.zip › 3B/Fig3B_IMQ2d_upper.tiff]

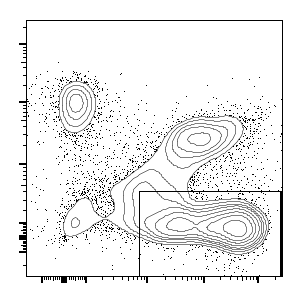

Supplement: Supplementary file 6 — Source data Fig. 4 [file 44321_2026_456_MOESM6_ESM.zip › 4D/Fig4D_BasalKeratinocyte_upper_mid.tiff]

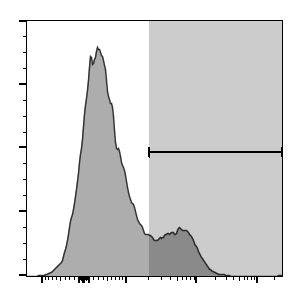

Supplement: Supplementary file 6 — Source data Fig. 4 [file 44321_2026_456_MOESM6_ESM.zip › 4D/Fig4D_Fibroblast_upper_right.tiff]

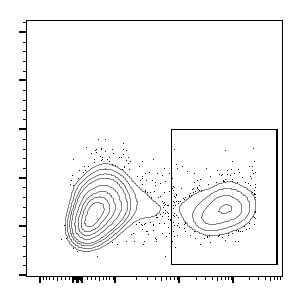

Supplement: Supplementary file 6 — Source data Fig. 4 [file 44321_2026_456_MOESM6_ESM.zip › 4D/Fig4D_Langerin_lower_right.tiff]

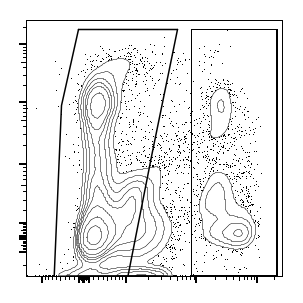

Supplement: Supplementary file 6 — Source data Fig. 4 [file 44321_2026_456_MOESM6_ESM.zip › 4D/Fig4D_Ter119CD45_lower_left.tiff]

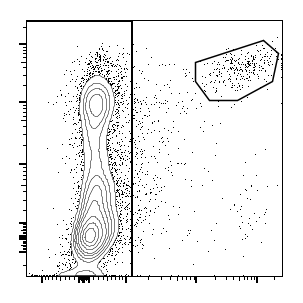

Supplement: Supplementary file 6 — Source data Fig. 4 [file 44321_2026_456_MOESM6_ESM.zip › 4D/Fig4D_EC_upper_left.tiff]

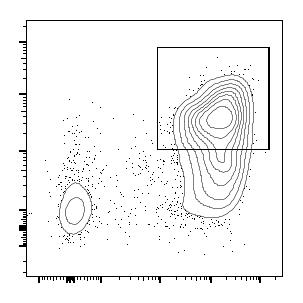

Supplement: Supplementary file 6 — Source data Fig. 4 [file 44321_2026_456_MOESM6_ESM.zip › 4D/Fig4D_MHC2_lower_midright.tiff]

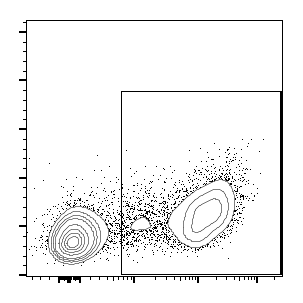

Supplement: Supplementary file 6 — Source data Fig. 4 [file 44321_2026_456_MOESM6_ESM.zip › 4D/Fig4D_CD11b_lower_midleft.tiff]

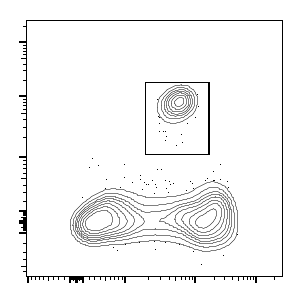

Supplement: Supplementary file 7 — Source data Fig. 5 [file 44321_2026_456_MOESM7_ESM.zip › 5D/Fig5D_anti-G-CSFi.d._right_panel.tiff]

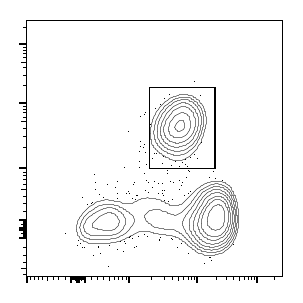

Supplement: Supplementary file 7 — Source data Fig. 5 [file 44321_2026_456_MOESM7_ESM.zip › 5D/Fig5D_IgGi.p._left_panel.tiff]

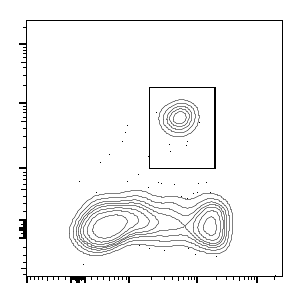

Supplement: Supplementary file 7 — Source data Fig. 5 [file 44321_2026_456_MOESM7_ESM.zip › 5D/Fig5D_anti-G-CSFi.p._left_panel.tiff]

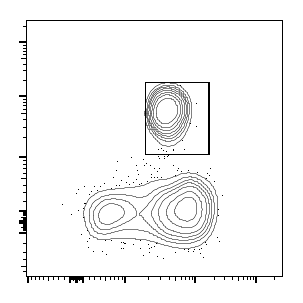

Supplement: Supplementary file 7 — Source data Fig. 5 [file 44321_2026_456_MOESM7_ESM.zip › 5D/Fig5D_IgGi.d._right_panel.tiff]

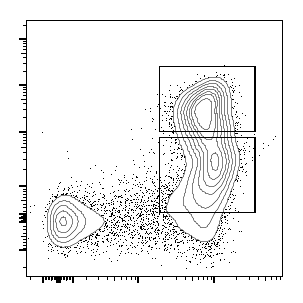

Supplement: Supplementary file 7 — Source data Fig. 5 [file 44321_2026_456_MOESM7_ESM.zip › 5C/Fig5C_IgGi.d._right_panel.tiff]

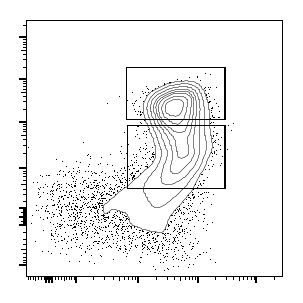

Supplement: Supplementary file 7 — Source data Fig. 5 [file 44321_2026_456_MOESM7_ESM.zip › 5C/Fig5C_IgGi.p._left_panel.tiff]

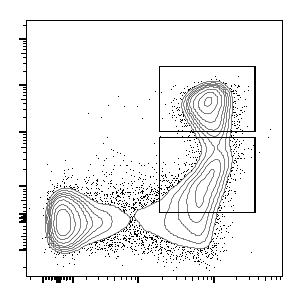

Supplement: Supplementary file 7 — Source data Fig. 5 [file 44321_2026_456_MOESM7_ESM.zip › 5C/Fig5C_anti-G-CSFi.d._right_panel.tiff]

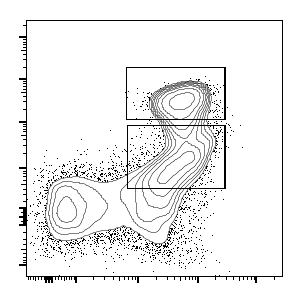

Supplement: Supplementary file 7 — Source data Fig. 5 [file 44321_2026_456_MOESM7_ESM.zip › 5C/Fig5C_anti-G-CSFi.p._left_panel.tiff]

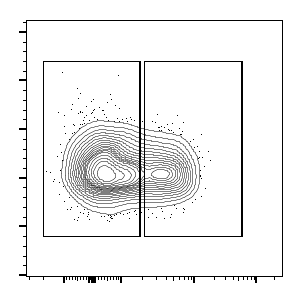

Supplement: Supplementary file 7 — Source data Fig. 5 [file 44321_2026_456_MOESM7_ESM.zip › 5B/Fig5B_anti-G-CSF_lower_right.tiff]

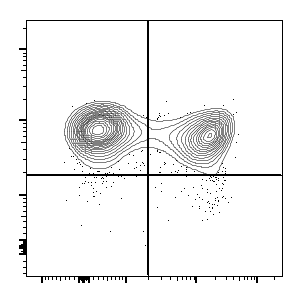

Supplement: Supplementary file 7 — Source data Fig. 5 [file 44321_2026_456_MOESM7_ESM.zip › 5B/Fig5B_anti-G-CSF_upper_right.tiff]

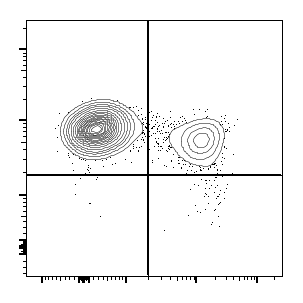

Supplement: Supplementary file 7 — Source data Fig. 5 [file 44321_2026_456_MOESM7_ESM.zip › 5B/Fig5B_IgG_upper_left.tiff]

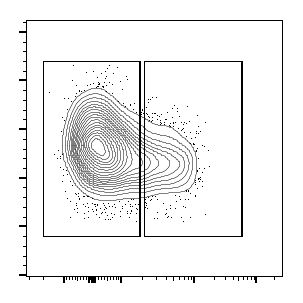

Supplement: Supplementary file 7 — Source data Fig. 5 [file 44321_2026_456_MOESM7_ESM.zip › 5B/Fig5B_IgG_lower_left.tiff]

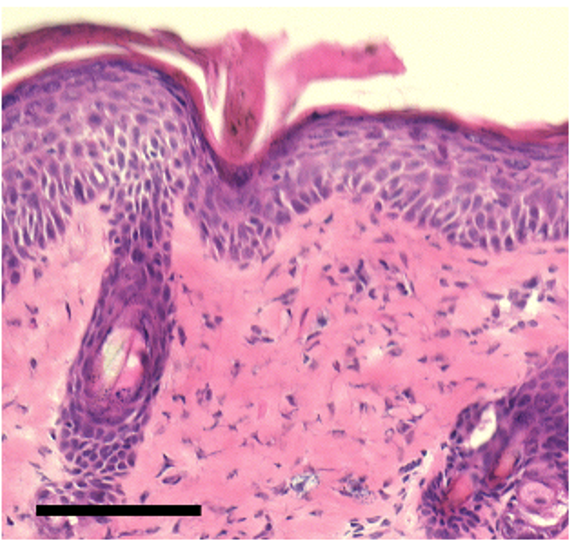

Supplement: Supplementary file 7 — Source data Fig. 5 [file 44321_2026_456_MOESM7_ESM.zip › 5E/Fig5E_Intraperitoneal_IgG.png]

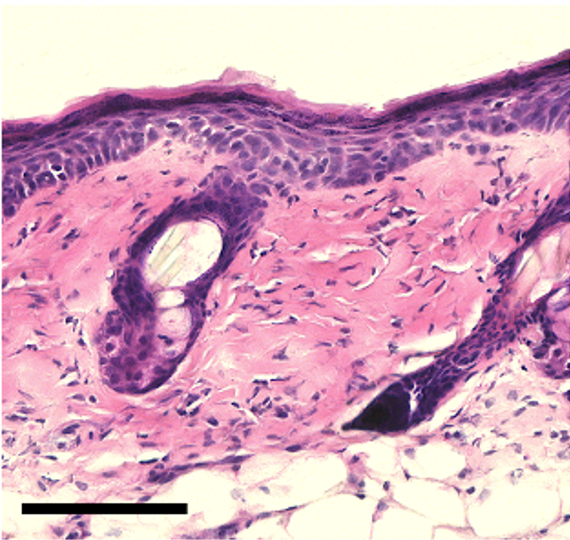

Supplement: Supplementary file 7 — Source data Fig. 5 [file 44321_2026_456_MOESM7_ESM.zip › 5E/Fig5E_Intraperitoneal_antiGCSF.png]

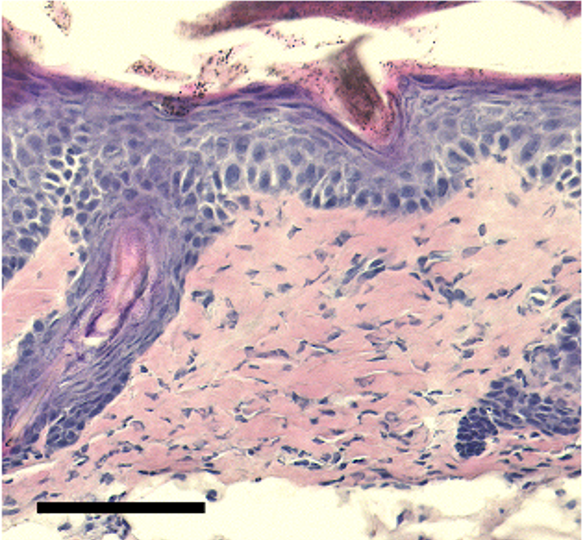

Supplement: Supplementary file 7 — Source data Fig. 5 [file 44321_2026_456_MOESM7_ESM.zip › 5E/Fig5E_Intradermal_IgG.png]

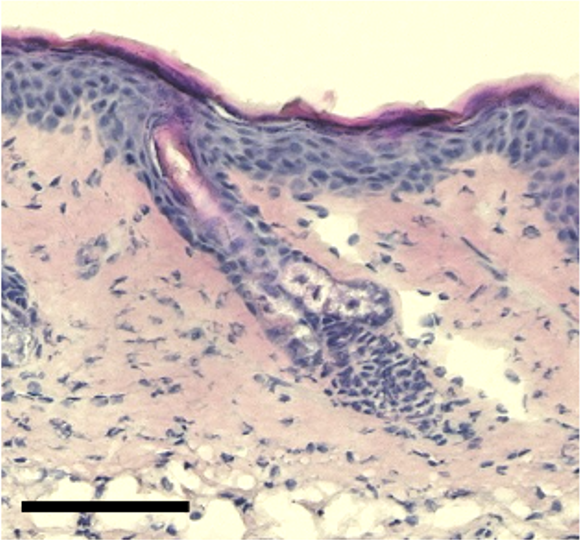

Supplement: Supplementary file 7 — Source data Fig. 5 [file 44321_2026_456_MOESM7_ESM.zip › 5E/Fig5E_Intradermal_antiGCSF.png]
