# Supplementary material for: Skin-derived G-CSF activates pathological granulopoiesis upon psoriasis
Source: EMBO Mol Med. 2026 Jun 16;18(7):2777–807. doi: 10.1038/s44321-026-00456-y (PMC13365241; doi:10.1038/s44321-026-00456-y)
Supplement: Supplementary file 9 — Expanded View Figures [file 44321_2026_456_MOESM9_ESM.pdf]

Expanded View Figures

Figure EV1. Characterization of clinical manifestations, myeloid cells, and the related factors in an imiquimod (IMQ)-induced skin psoriasis model.

(**A**) Upper: representative images of dorsal skin treated topically with daily Vas/IMQ from 0–3 d (4 times). Lower: Clinical score such as erythema and scaling from the dorsal skin treated with Vas/IMQ ($n = 3$ each). 2way ANOVA/Sidak comparison: $p =$ n.d. (0 d), $p < 0.0001$ (1 d), $p = 0.0005$ (2 d), $p < 0.0001$ (3 d), $p = 0.0001$ (4 d) for erythema, and $p =$ n.d. (0 d), $p < 0.0001$ (1 d), $p < 0.0002$ (2 d), $p < 0.0001$ (3 d), $p < 0.0001$ (4 d) for scaling. The scale bars in each figure represent 1 cm long. (**B**) mRNA expression of dermatitis-related cytokines in dorsal skin treated with Vas ($n = 3$–5) or IMQ ($n = 3$–5). *t*-test/Mann–Whitney test: $p = 0.0306$ (*Il17a*), $p = 0.0178$ (*Il17f*), $p > 0.9999$ (*Il23*), $p =$ n.d. (*Il22*), $p = 0.0526$ (*Tnfa*), $p = 0.0079$ (*Il6*). (**C**) Representative FACS gating strategy plots of myeloid cell fractions from dorsal skin treated with Vas or IMQ. (**D**) mRNA expression of chemokines in the dorsal skin treated with Vas or IMQ ($n = 3$–5 for each). *t*-test/Mann–Whitney test: $p = 0.0079$ (*Cxcl1*), $p = 0.0159$ (*Cxcl2*), $p = 0.0500$ (*Cxcl5*), $p = 0.9758$ (*Ccl2*). (**E**) Number and percentage of myeloid cell fractions in the non-lesional abdominal skin (Vas: $n = 4$, IMQ: $n = 4$); statistical analysis *t*-test: $p = 0.6224$ (Neutrophil), $p = 0.0891$ (Ly6C^{+}), $p = 0.1634$ (Ly6C^{-}) for cell number, $p = 0.1255$ (Neutrophil), $p = 0.2411$ (Ly6C^{+}), $p = 0.8402$ (Ly6C^{-}) for percentage. (**F**) Representative 3-dimensional (3D) intravital microscopic images of dorsal skin at 0 d, 1 d, and 2 d after Vas or IMQ treatment. Left images show raw data with Ly6G^{+} cells (red), CD31^{+} cells (green), and SHG (second harmonic generation) (blue). Right images show spot and surface transformed data with spot (Ly6G^{+}) and surface (CD31^{+}). (**G**) Representative images of the transformed 3D-intravital microscopy of neutrophil (left) and random spot (right) proximity distribution to blood vessel in the 2 d IMQ-skin, scale bar = 50 µm. (**H**) Bar graphs showing the comparative distribution of neutrophils and random spots to the blood vessel in the IMQ-skin at 0 d (upper) and 1 d of IMQ treatment (lower) ($n = 3$ for each). Data are pooled from ≥ 2 independent experiments with each dot in the graphs representing one experimental subject. *t*-test: $p = 0.2027$ (> 0–10), $p = 0.2619$ (> 10–20), $p = 0.9444$ (> 20–30), $p = 0.3750$ (> 30–40), $p = 0.5581$ (> 40–50), $p < 0.0001$ (> 50) for 0d-IMQ, $p = 0.0338$ (> 0–10), $p = 0.0049$ (> 10–20), $p = 0.1529$ (> 20–30), $p = 0.0290$ (> 30–40), $p = 0.0572$ (> 40–50), $p = 0.0050$ (> 50) for 1d-IMQ. For 3D-intravital microscopy, experiment was done in 1 subject per group with each dot in the graphs representing individual acquired tissue plane. Data are shown as mean ± S.E. *$p < 0.05$; **$p < 0.01$; ***$p < 0.001$; ****$p < 0.0001$.

© The Author(s)

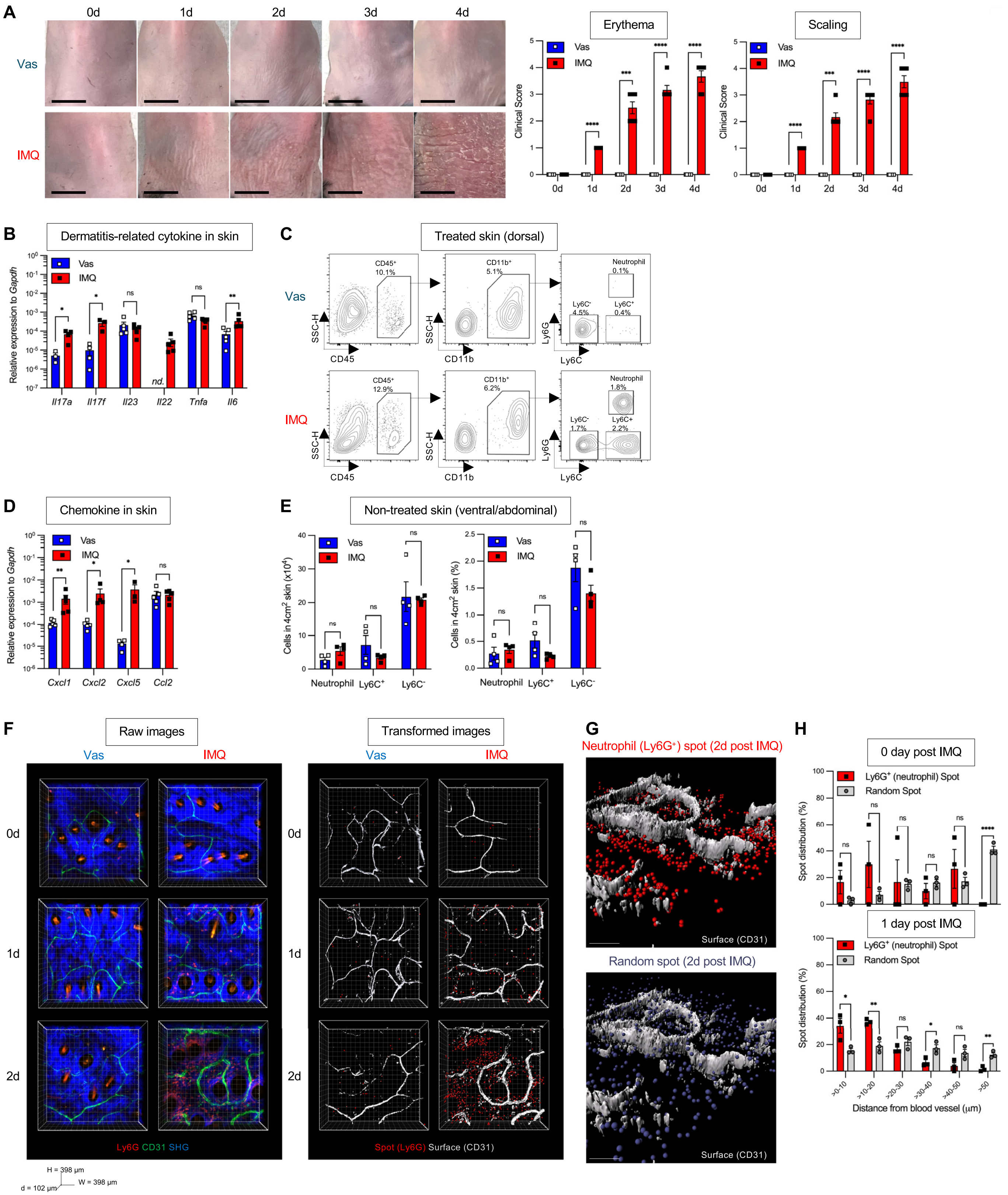
A
0d
1d
2d
3d
4d
Vas
IMQ
Erythema
Scaling
Clinical Score
B
Dermatitis-related cytokine in skin
Relative expression to Gapdh
Il17a
Il17f
Il23
Il22
Tnfa
Il6
nd.
C
Treated skin (dorsal)
CD45+ 10.1%
CD11b+ 5.1%
Neutrophil 0.1%
CD45+ 12.9%
CD11b+ 6.2%
Neutrophil 1.8%
SSC-H
CD45
CD11b
Ly6G
Ly6C
D
Chemokine in skin
Cxcl1
Cxcl2
Cxcl5
Ccl2
E
Non-treated skin (ventral/abdominal)
Neutrophil
F
Raw images
Transformed images
Ly6G CD31 SHG
Spot (Ly6G) Surface (CD31)
H = 398 μm
W = 398 μm
d = 102 μm
G
Neutrophil (Ly6G+) spot (2d post IMQ)
Random spot (2d post IMQ)
Surface (CD31)
H
0 day post IMQ
1 day post IMQ
Ly6G+ (neutrophil) Spot
Random Spot
Spot distribution (%)
Distance from blood vessel (μm)

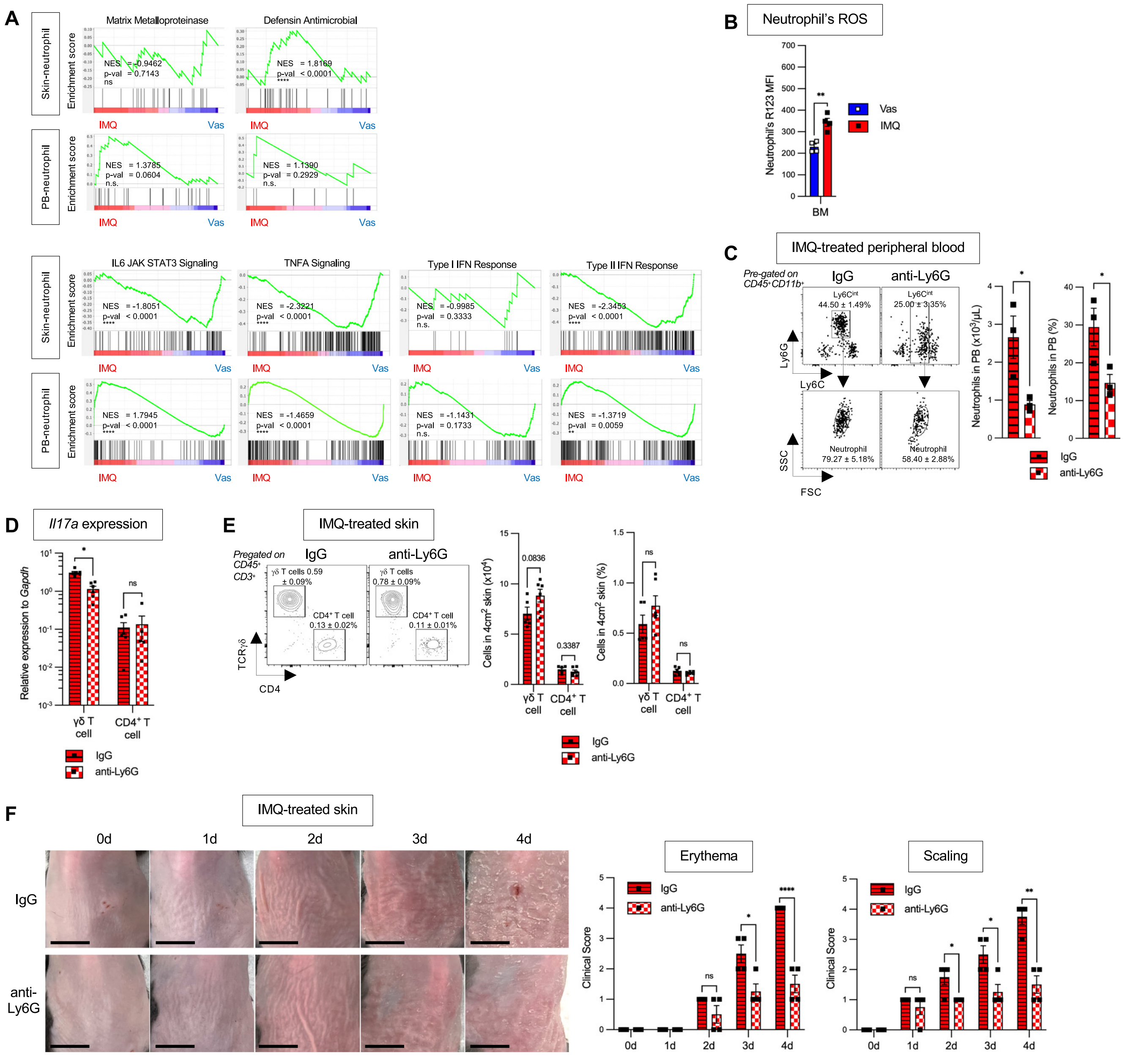

Figure EV2. Overactive neutrophils have pathological function in psoriasis.

(**A**) GSEA analysis of neutrophils from Vas-treated ($n = 3$–5) and IMQ-treated skin and PB ($n = 3$–5). Signal2Noise/FWER p-value: $p = 0.7143$ (Matrix Metalloproteinase), $p < 0.0001$ (Defensin Antimicrobial), $p < 0.0001$ (IL6 JAK STAT3 Signaling), $p < 0.0001$ (TNFA Signaling), $p = 0.3333$ (Type 1 IFN Response), $p < 0.0001$ (Type 2 IFN Response) for skin neutrophil and $p = 0.0604$ (Matrix Metalloproteinase), $p = 0.02929$ (Defensin Antimicrobial), $p < 0.0001$ (IL6 JAK STAT3 Signaling), $p < 0.0001$ (TNFA Signaling), $p = 0.1733$ (Type 1 IFN Response), $p = 0.0059$ (Type 2 IFN Response) for PB neutrophil. (**B**) ROS level in the neutrophils measured from BM of Vas- and IMQ-treated mice ($n = 4$ for each). t-test: $p = 0.0023$. (**C**) Representative FACS plot and quantification of peripheral blood neutrophils at 4 d from IgG or anti-Ly6G antibody injected IMQ-induced mice ($n = 3$ for each). Mann–Whitney test: $p = 0.0286$ (Neutrophil number), $p = 0.0286$ (Neutrophil percentage). (**D**) *Il17a* transcript measured in sorted phenotypic γδ T cells and CD4$^+$ helper T cells of IMQ-treated mice injected with either IgG control or anti-Ly6G antibody ($n = 5$–6 for each). t-test: $p = 0.0010$ (γδ T-cell), $p = 0.7979$ (CD4$^+$ T-cell). (**E**) Percentage and absolute cell number of γδ T cells and CD4$^+$ helper T cells from IMQ-treated mice administered with either IgG control or anti-G-CSF antibody ($n = 5$–6 for each). t-test: $p = 0.0836$ (γδ T-cell), $p = 0.3387$ (CD4$^+$ T-cell) for cell number, $p = 0.2062$ (γδ T-cell), $p = 0.2695$ (CD4$^+$ T-cell) for percentage. (**F**) Clinical examination of dorsal IMQ-skin at 0-4 d with IgG or anti-Ly6G treatment. Upper: representative skin images, lower: erythema and scaling scoring (IgG: $n = 4$, anti-Ly6G: $n = 4$). t-test: $p =$ n.d. (0 d), $p =$ *n.d.* (1 d), $p =$ n.d. (2 d), $p = 0.0170$ (3 d), $p = 0.0001$ (4 d) for erythema, $p =$ n.d. (0 d), $p = 0.3559$ (1 d), $p = 0.0240$ (2 d), $p = 0.0170$ (3 d), $p = 0.0011$ (4 d) for scaling. The black line in each picture measures 1 cm scale. Data are pooled from ≥ 2 independent experiments with each dot shown in the bar graph represents data from each individual and shown as mean ± S.E. For RNA-seq, analysis was done to the data pooled from ≥2 replicates. *$p < 0.05$; **$p < 0.01$; ***$p < 0.001$; ****$p < 0.0001$.

 © The Author(s)

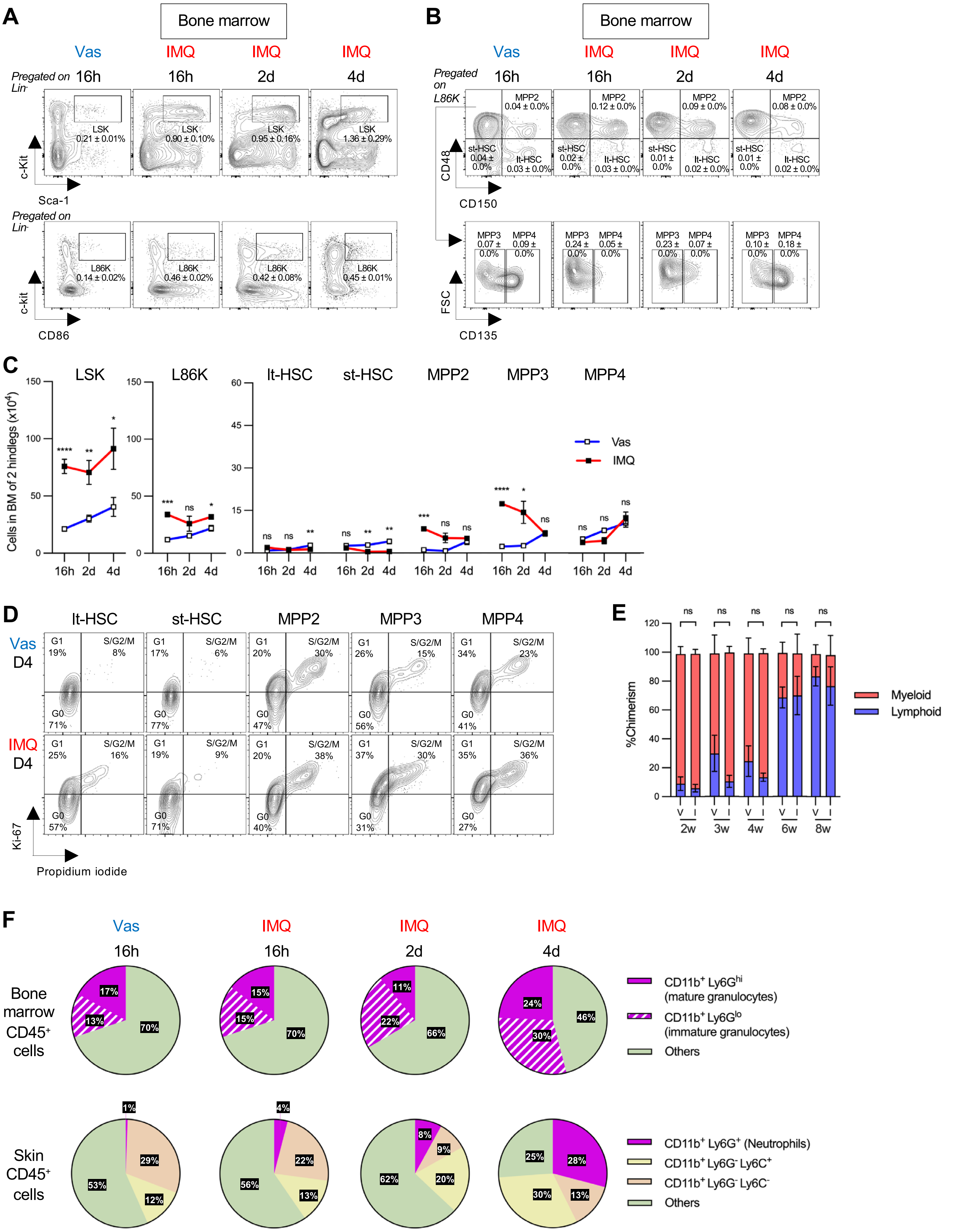
A
Bone marrow
Vas
IMQ
IMQ
IMQ
Pregated on Lin-
16h
16h
2d
4d
LSK 0.21 ± 0.01%
LSK 0.90 ± 0.10%
LSK 0.95 ± 0.16%
LSK 1.36 ± 0.29%
c-Kit
Sca-1
L86K 0.14 ± 0.02%
L86K 0.46 ± 0.02%
L86K 0.42 ± 0.08%
L86K 0.45 ± 0.01%
c-kit
CD86
B
Bone marrow
Pregated on L86K
MPP2
st-HSC
lt-HSC
CD48
CD150
MPP3
MPP4
FSC
CD135
C
LSK
L86K
lt-HSC
st-HSC
MPP2
MPP3
MPP4
Cells in BM of 2 hindlegs (x10⁴)
Vas
IMQ
D
lt-HSC
st-HSC
MPP2
MPP3
MPP4
Vas D4
IMQ D4
G1
S/G2/M
G0
Ki-67
Propidium iodide
E
%Chimerism
Myeloid
Lymphoid
2w
3w
4w
6w
8w
F
Bone marrow CD45⁺ cells
CD11b⁺ Ly6Ghi (mature granulocytes)
CD11b⁺ Ly6Glo (immature granulocytes)
Others
Skin CD45⁺ cells
CD11b⁺ Ly6G⁺ (Neutrophils)
CD11b⁺ Ly6G⁻ Ly6C⁺
CD11b⁺ Ly6G⁻ Ly6C⁻
Others

Figure EV3. Psoriasis induces HSPC expansion and granulopoiesis.

(**A**) FACS plots of LSK (Lin$^-$ Sca-1$^+$c-Kit$^+$) (upper) and L86K (Lin$^-$CD86$^+$c-Kit$^+$) cells (lower) in BM at 16 h, 2 d, and 4 d after Vas or IMQ treatment ($n = 3$–6 for each). (**B**) FACS plot of immunophenotypic lt-HSC, st-HSC, MPP2, MPP3, and MPP4 defined by L86K (EV3A, lower panel) in the BM of mice treated with Vas/IMQ ($n = 3$–6 each). (**C**) Absolute number of LSK (left), L86K (middle) and L86K-defined HSPC (lt-HSC, st-HSC, MPP2-4) (right) in the BM of mice treated with Vas/IMQ ($n = 3$–6 for each). *t*-test: $p < 0.0001$ (16 h), $p = 0.0041$ (2 d), $p = 0.0279$ (4 d) for LSK, $p = 0.0009$ (16 h), $p = 0.1934$ (2 d), $p = 0.0239$ (4 d) for L86K, $p = 0.0677$ (16 h), $p = 0.8633$ (2 d), $p = 0.0059$ (4 d) for lt-HSC, $p = 0.1845$ (16 h), $p = 0.0050$ (2 d), $p = 0.0017$ (4 d) for st-HSC, $p = 0.0001$ (16 h), $p = 0.0546$ (2 d), $p = 0.3169$ (4 d) for MPP2, $p < 0.0001$ (16 h), $p = 0.0405$ (2 d), $p = 0.8809$ (4 d) for MPP3, $p = 0.2698$ (16 h), $p = 0.0536$ (2 d), $p = 0.4749$ (4 d) for MPP4. (**D**) Representative FACS plots of cell cycle analysis of the HSPC fractions in Vas- and IMQ-treated BM ($n = 5$ for each). (**E**) Lympho-myeloid contribution in PB of transplants with donor LSK from Vas-treated ($n = 3$) or IMQ-treated ($n = 4$) BM. *t*-test: $p = 0.5751$ (2w), $p = 0.1522$ (3w), $p = 0.2866$ (4w), $p = 0.9211$ (6w), $p = 0.7372$ (8w) for Myeloid fraction. (**F**) Pie charts depicting relative composition of hematopoietic cells (CD45$^+$) in BM (upper) and skin (lower) at 16 h, 2 d, and 4 d after Vas or IMQ treatment ($n = 3$–6 each). Data are pooled from ≥ 2 independent experiments. Each dot shown in the bar graph represents data from each individual and shown as mean ± S.E. *$p < 0.05$; **$p < 0.01$; ***$p < 0.001$; ****$p < 0.0001$.

 © The Author(s)

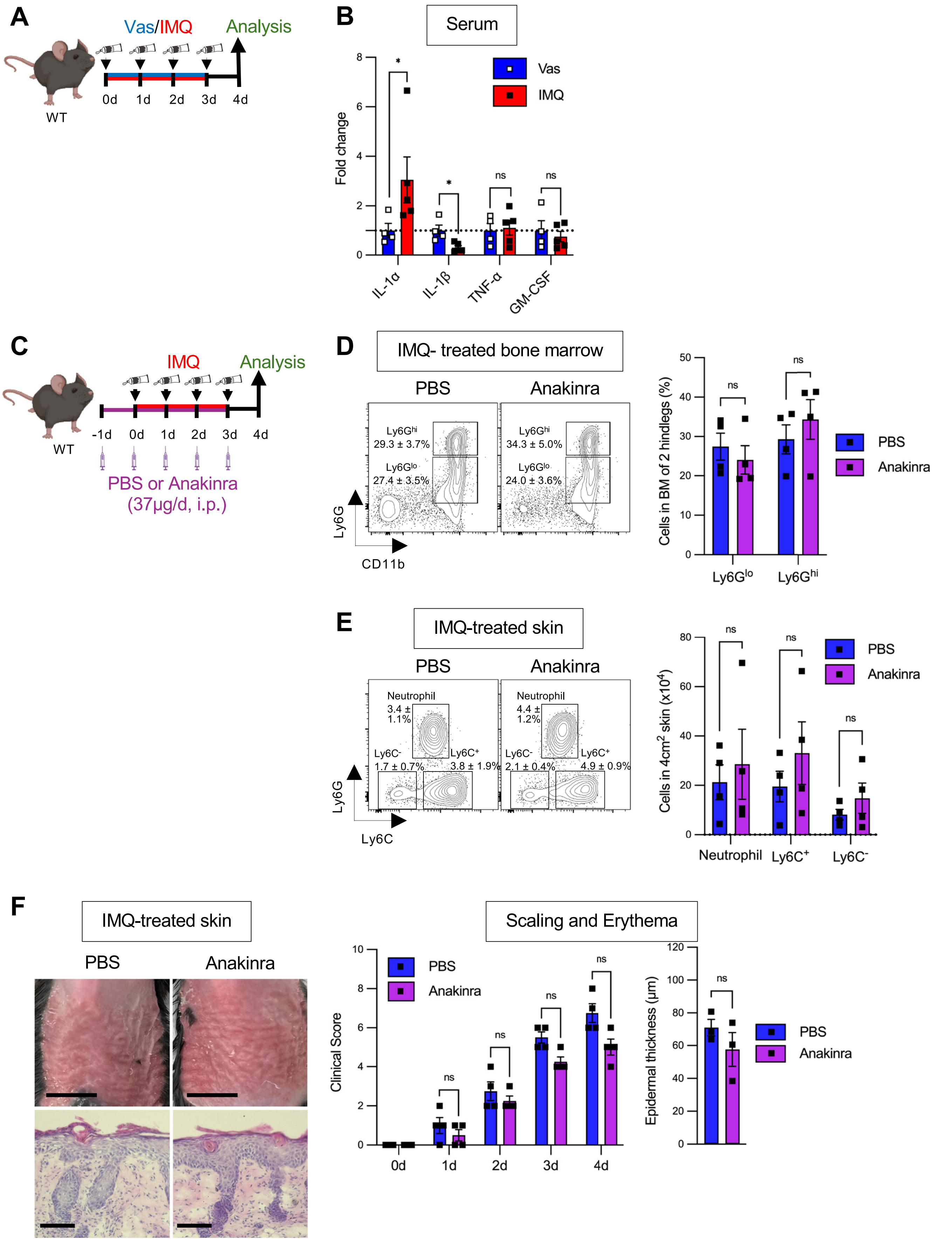
A
Vas/IMQ
Analysis
WT
0d 1d 2d 3d 4d
B
Serum
Vas
IMQ
Fold change
IL-1α IL-1β TNF-α GM-CSF
C
IMQ
Analysis
WT
-1d 0d 1d 2d 3d 4d
PBS or Anakinra
(37μg/d, i.p.)
D
IMQ- treated bone marrow
PBS
Anakinra
Ly6Ghi 29.3 ± 3.7%
Ly6Glo 27.4 ± 3.5%
Ly6Ghi 34.3 ± 5.0%
Ly6Glo 24.0 ± 3.6%
Ly6G
CD11b
Cells in BM of 2 hindlegs (%)
Ly6Glo Ly6Ghi
E
IMQ-treated skin
PBS
Anakinra
Neutrophil 3.4 ± 1.1%
Ly6C- 1.7 ± 0.7%
Ly6C+ 3.8 ± 1.9%
Neutrophil 4.4 ± 1.2%
Ly6C- 2.1 ± 0.4%
Ly6C+ 4.9 ± 0.9%
Ly6G
Ly6C
Cells in 4cm2 skin (x104)
Neutrophil Ly6C+ Ly6C-
F
IMQ-treated skin
PBS
Anakinra
Scaling and Erythema
Clinical Score
0d 1d 2d 3d 4d
Epidermal thickness (μm)
ns

Figure EV4. IL-1 signals do not contribute to psoriasis-driven emergency granulopoiesis.

(**A**) Experimental scheme for (**B**): Vaseline (Vas) or Imiquimod (IMQ) topically applied to the dorsal skin daily for 4 d and serum analysis was conducted at 4 d. (**B**) Cytokines protein measurement from the sera (Vas: $n = 4$, IMQ: $n = 5$). *t*-test/Mann–Whitney test: $p = 0.0317$ (IL-1α), $p = 0.0164$ (IL-1β), $p = 0.7905$ (TNF-α), $p = 0.5896$ (GM-CSF). (**C**) Experimental scheme of IMQ-induced psoriasis (0–3 d) with daily PBS/Anakinra intraperitoneal injection (from −1 d to 3 d) and analyzed at 4 d for results depicted in (**D–H**). (**D**) Representative FACS plots of immature ($Ly6G^{lo}$) and mature ($Ly6G^{hi}$) granulocytes pregated on $CD45^+$ BM cells (left). The proportion of $Ly6G^{lo}$ and $Ly6G^{hi}$ cells ($n = 4$ for each) (right). *t*-test: $p = 0.5179$ ($Ly6G^{lo}$), $p = 0.4510$ ($Ly6G^{hi}$). (**E**) Representative FACS plots of myeloid cell fractions in the dorsal skin pregated on $CD45^+CD11b^+$ cells (left). Cell number quantification graph of the IMQ-induced mice treated with PBS/Anakinra ($n = 4$ for each) (right). *t*-test: $p = 0.6624$ (Neutrophil), $p = 0.3730$ ($Ly6C^+$), $p = 0.3453$ ($Ly6C^-$). (**F**) Skin clinical examination upon psoriasis disease course. Representative naked skin images with scale bar = 1 cm (left upper panel) and skin histology by HE staining with black line indicates 100 µm (left lower panel). Daily clinical scoring (middle) and epidermal thickness measurement of IMQ-induced psoriatic skin following treatment with PBS/Anakinra ($n = 3$–4 for each). *t*-test/Mann–Whitney test: $p > 0.9999$ (0 d), $p = 0.6571$ (1 d), $p = 0.7143$ (2 d), $p = 0.0857$ (3 d), $p = 0.0857$ (4 d) for clinical score, $p = 0.3123$ for epidermal thickness. Data are pooled from ≥ 2 independent experiments with each dot shown in the bar chart represents individual subject and shown as mean ± S.E. *$p < 0.05$; **$p < 0.01$; ***$p < 0.001$; ****$p < 0.0001$.

© The Author(s)

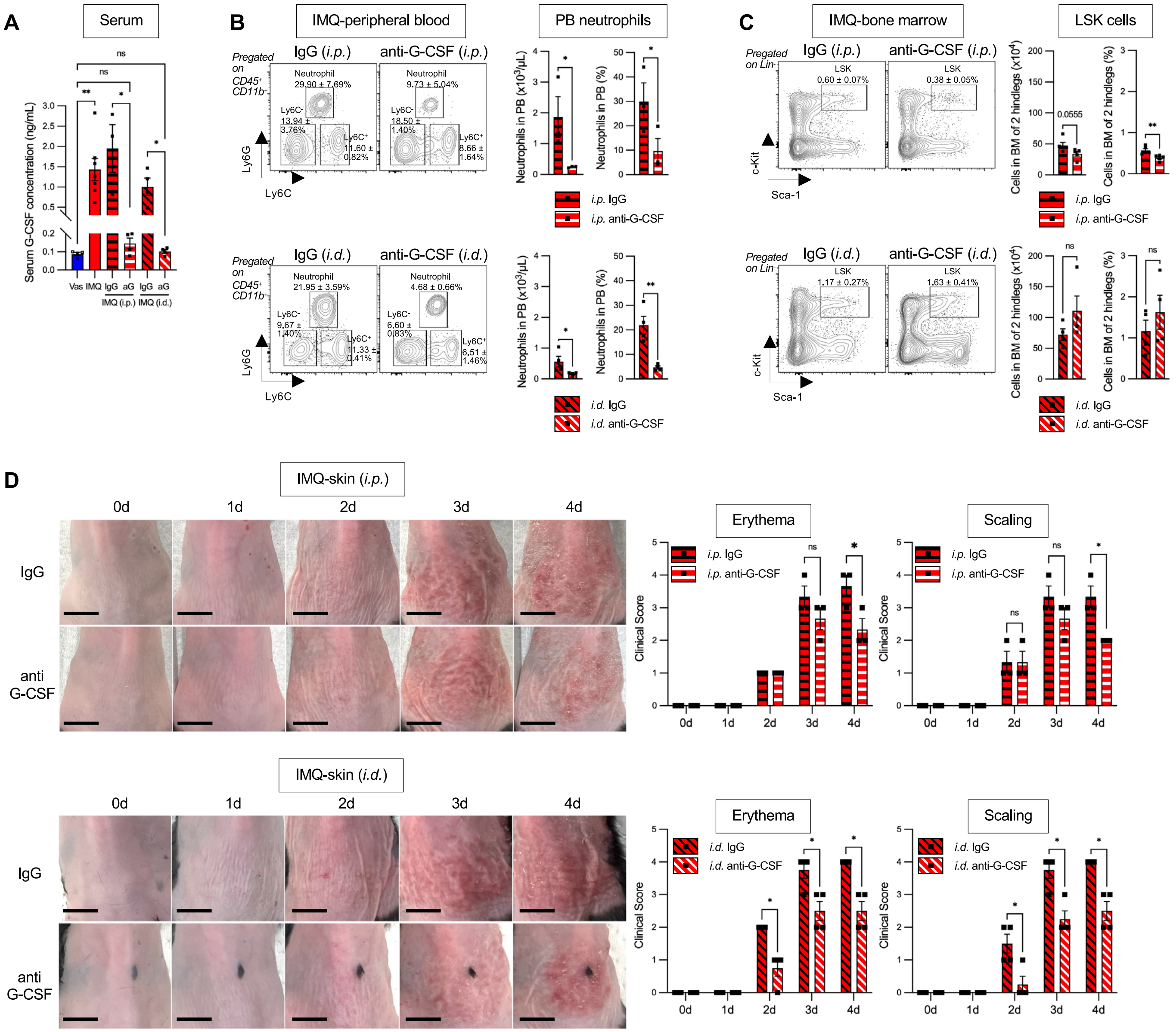

Figure EV5. G-CSF neutralization reduces circulating neutrophils, hematopoietic activation in the BM, and psoriasis clinical manifestation.

(**A**) G-CSF measured in the serum of mice with the following treatments (from left to right): Vas alone ($n = 4$), IMQ alone ($n = 7$), *i.p.* IgG Ab + IMQ ($n = 3$), *i.p.* anti-G-CSF Ab + IMQ ($n = 4$), *i.d.* IgG Ab + IMQ ($n = 4$), and *i.d.* anti-G-CSF Ab + IMQ ($n = 4$). Kruskal–Wallis test/Dunn's test: $p = 0.0010$ (Vas vs IMQ), $p = 0.4054$ (Vas vs IMQ + anti-G-CSF *i.p.*), $p = 0.6774$ (Vas vs IMQ + anti-G-CSF *i.d.*), $p = 0.0312$ (IMQ + IgG *i.p.* vs IMQ + anti-G-CSF *i.p.*), $p = 0.0468$ (IMQ + IgG *i.d.* vs IMQ + anti-G-CSF *i.d.*). (**B**) Representative FACS plot along with the proportional and absolute cell number of circulating phenotypic neutrophils bar chart in IMQ-induced psoriasis mice treated with IgG/anti-GCSF ($n = 3$–4/group). *t*-test/Mann–Whitney test: $p = 0.0050$ (IgG *i.p.* vs anti-G-CSF *i.p.*), $p = 0.0286$ (IgG *i.d.* vs anti-G-CSF *i.d.*) for cell number and $p = 0.0046$ (IgG *i.p.* vs anti-G-CSF *i.p.*), $p = 0.0032$ (IgG *i.d.* vs anti-G-CSF *i.d.*) for percentage. (**C**) FACS analysis of the $Lin^-c\text{-}Kit^+Sca\text{-}1^+$ (LSK) in the BM of IMQ-induced psoriasis treated with IgG/anti-G-CSF ($n = 4$–5 each). *t*-test/Mann–Whitney test: $p = 0.0555$ (IgG *i.p.* vs anti-G-CSF *i.p.*), $p = 0.1878$ (IgG *i.d.* vs anti-G-CSF *i.d.*) for cell number and $p = 0.0080$ (IgG *i.p.* vs anti-G-CSF *i.p.*), $p = 0.3839$ (IgG *i.d.* vs anti-G-CSF *i.d.*) for percentage. (**D**) Psoriasis clinical manifestation of the IMQ-induced mice which was monitored daily at the naked dorsal skin (upper) and clinically (erythema and scaling) graphed (lower) with *i.p.* (top panel) or *i.d.* (bottom panel) injection of IgG/anti-G-CSF ($n = 3$–4 for each). *t*-test: $p =$ n.d. (0 d), $p =$ n.d. (1 d), $p =$ n.d. (2 d), $p = 0.2302$ (3 d), $p = 0.0474$ (4 d) for erythema, *i.p.*, $p =$ n.d. (0 d), $p =$ n.d. (1 d), $p > 0.9999$ (2 d), $p = 0.2302$ (3 d), $p = 0.0161$ (4 d) for scaling, *i.p.*, $p =$ n.d. (0 d), $p =$ n.d. (1 d), $p = 0.0025$ (2 d), $p = 0.0170$ (3 d), $p = 0.0020$ (4 d) for erythema, *i.d.*, $p =$ n.d. (0 d), $p =$ n.d. (1 d), $p = 0.0170$ (2 d), $p = 0.0054$ (3 d), $p = 0.0020$ (4 d) for scaling *i.d.* The line bar embedded in each picture signify 1 cm scale. Data are pooled from ≥ 2 independent experiments with each dot shown in the chart represents the measurement from 1 experimental subject and shown as mean ± S.E. $^*p < 0.05$; $^{**}p < 0.01$; $^{***}p < 0.001$; $^{****}p < 0.0001$.

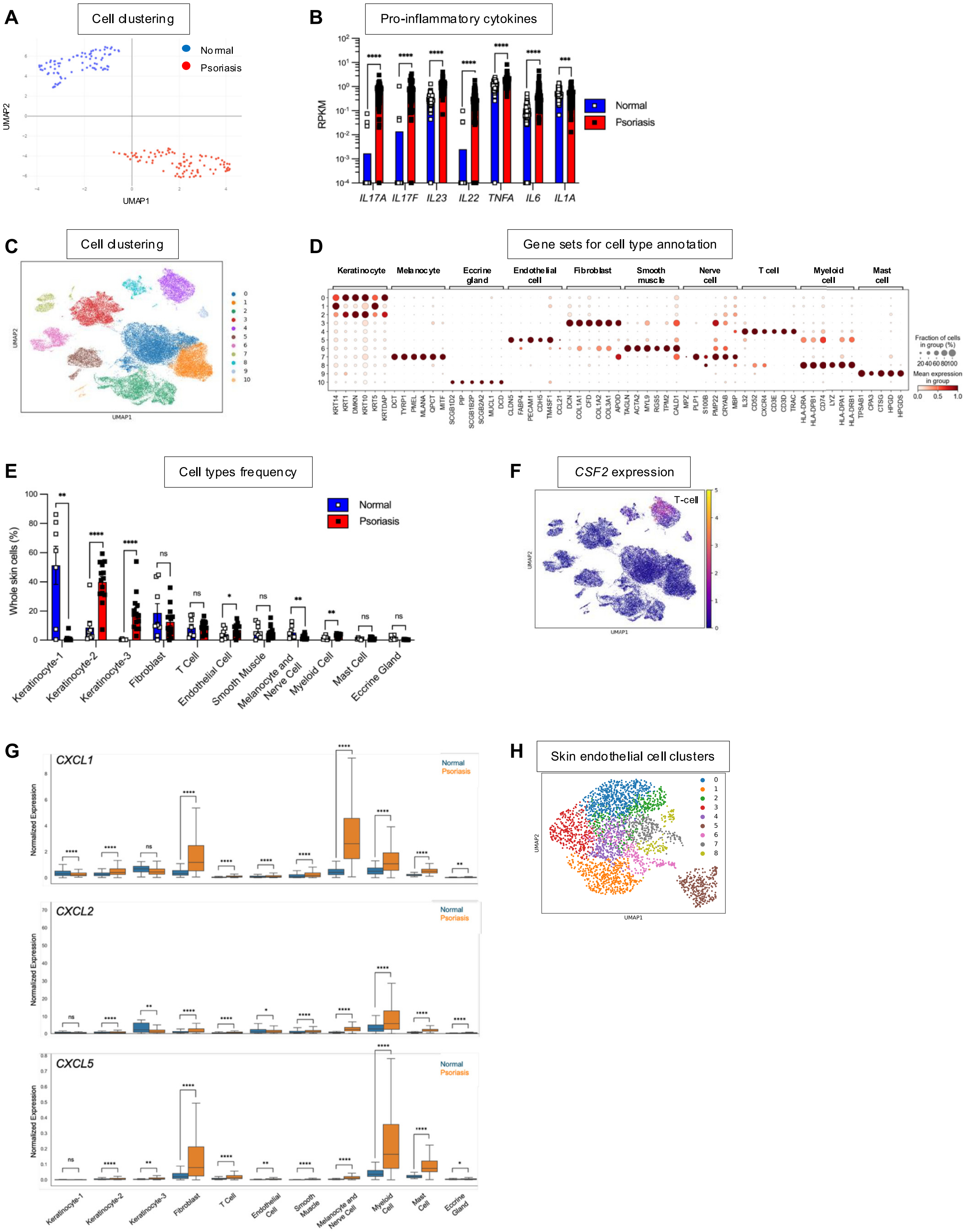
A
Cell clustering
Normal
Psoriasis
UMAP2
UMAP1
B
Pro-inflammatory cytokines
RPKM
Normal
Psoriasis
IL17A IL17F IL23 IL22 TNFA IL6 IL1A
C
Cell clustering
UMAP2
UMAP1
D
Gene sets for cell type annotation
Keratinocyte
Melanocyte
Eccrine gland
Endothelial cell
Fibroblast
Smooth muscle
Nerve cell
T cell
Myeloid cell
Mast cell
Fraction of cells in group (%)
Mean expression in group
E
Cell types frequency
Whole skin cells (%)
Normal
Psoriasis
Keratinocyte-1
Keratinocyte-2
Keratinocyte-3
Fibroblast
T Cell
Endothelial Cell
Smooth Muscle
Melanocyte and Nerve Cell
Myeloid Cell
Mast Cell
Eccrine Gland
F
CSF2 expression
T-cell
UMAP2
UMAP1
G
CXCL1
CXCL2
CXCL5
Normalized Expression
Normal
Psoriasis
H
Skin endothelial cell clusters
UMAP2
UMAP1

 © The Author(s)

Figure EV6. Clustering and relative transcript measurement of human psoriasis skin biopsies.

(**A**) UMAP clustering of RNA-seq data from healthy and psoriatic human skin biopsy (normal: $n = 82$, psoriasis: $n = 92$). (**B**) Relative expression of dermatitis-related cytokines (normal: $n = 82$, psoriasis: $n = 92$). Mann–Whitney test: $p < 0.0001$ (*IL17A*), $p < 0.0001$ (*IL17F*), $p < 0.0001$ (*IL23*), $p < 0.0001$ (*IL22*), $p < 0.0001$ (TNF*A*), $p < 0.0001$ (*IL6*), $p = 0.0007$ (*IL1A*). (**C–H**) Re-analysis of single-cell RNA-seq dataset GSE173706 (normal: $n = 8$, psoriasis: $n = 14$) consisting of UMAP clustering (**C**), gene list for clusters annotation (**D**), cell frequency counting on each annotated cell types (**E**). *t*-test/Mann–Whitney test: $p = 0.0023$ (Keratinocyte-1), $p < 0.0001$ (Keratinocyte-2), $p < 0.0001$ (Keratinocyte-3), $p = 0.1568$ (Fibroblast), $p = 0.4034$ (T-cell), $p = 0.0499$ (Endothelial cell), $p = 0.3319$ (Smooth muscle), $p = 0.0083$ (Melanocyte and Nerve cell), $p = 0.0017$ (Myeloid cell), $p = 0.4803$ (Mast cell), $p = 0.1128$ (Eccrine gland), *CSF2* expression mapped in the UMAP clustering (**F**), *CXCL1/CXCL2/CXCL5* expression (**G**). *t*-test: $p < 0.0001$ (Keratinocyte-1), $p < 0.0001$ (Keratinocyte-2), $p = 0.0813$ (Keratinocyte-3), $p < 0.0001$ (Fibroblast), $p < 0.0001$ (T-cell), $p < 0.0001$ (Endothelial cell), $p < 0.0001$ (Smooth muscle), $p < 0.0001$ (Melanocyte and Nerve cell), $p < 0.0001$ (Myeloid cell), $p < 0.0001$ (Mast cell), $p = 0.0045$ (Eccrine gland) for *CXCL1*. $p = 0.0602$ (Keratinocyte-1), $p < 0.0001$ (Keratinocyte-2), $p = 0.0032$ (Keratinocyte-3), $p < 0.0001$ (Fibroblast), $p < 0.0001$ (T-cell), $p = 0.0129$ (Endothelial cell), $p < 0.0001$ (Smooth muscle), $p < 0.0001$ (Melanocyte and Nerve cell), $p < 0.0001$ (Myeloid cell), $p < 0.0001$ (Mast cell), $p < 0.0001$ (Eccrine gland) for *CXCL2*. $p = 0.3313$ (Keratinocyte-1), $p < 0.0001$ (Keratinocyte-2), $p < 0.0001$ (Keratinocyte-3), $p < 0.0001$ (Fibroblast), $p < 0.0001$ (T-cell), $p = 0.0021$ (Endothelial cell), $p < 0.0001$ (Smooth muscle), $p < 0.0001$ (Melanocyte and Nerve cell), $p < 0.0001$ (Myeloid cell), $p < 0.0001$ (Mast cell), $p = 0.0390$ (Eccrine gland) for *CXCL5*. Following are the detailed analytical data of each (value of Normal/value of Psoriasis): *CXCL1*: Keratinocyte-1 (min = 0.0054/0.0436, max = 2.8499/1.1675, median = 0.3033/0.2212, mean = 0.3706/0.2717, lower whisker = 0.0054/0.0436, upper whisker = 1.0234/0.6486, 95th percentile = 0.9001/0.5699), Keratinocyte-2 (min = 0.0231/0.0119, max = 1.8105/20.4381, median = 0.2590/0.4055, mean = 0.2933/0.5699, lower whisker = 0.0231/0.0119, upper whisker = 0.6936/1.3328, 95th percentile = 0.6271/1.5084), Keratinocyte-3 (min = 0.0607/0.0281, max = 1.2440/5.5007, median = 0.6806/0.4410, mean = 0.6481/0.5593, lower whisker = 0.0607/0.0281, upper whisker = 1.2440/1.2734, 95th percentile = 1.0515/1.4100), Fibroblast (min = 0.0366/0.0617, max = 5.4288/27.4380, median = 0.3219/1.1753, mean = 0.4593/1.9295, lower whisker = 0.0366/0.0617, upper whisker = 1.0833/5.3657, 95th percentile=1.2122/6.0698), T-Cell (min = 0.0062/0.0065, max = 0.5589/6.4854, median = 0.0340/0.0861, mean = 0.0474/0.1274, lower whisker = 0.0062/0.0065, upper whisker = 0.1099/0.2845, 95th percentile = 0.1260/0.3394), Endothelial Cell (min = 0.0032/0.0052, max = 1.2288/2.6625, median = 0.0688/0.0777, mean = 0.1035/0.1456, lower whisker = 0.0032/0.0052, upper whisker = 0.2650/0.3627, 95th percentile = 0.2890/0.5058), Smooth Muscle (min = 0.0034/0.0055, max = 2.2183/4.5404, median = 0.0847/0.1834, mean = 0.1913/0.3389, lower whisker = 0.0034/0.0055, upper whisker = 0.5502/0.8210, 95th percentile=0.7248/1.1467), Melanocyte and Nerve Cell (min = 0.0194/0.0716, max = 1.8830/16.2340, median = 0.4136/2.6095, mean = 0.4975/3.4355, lower whisker = 0.0194/0.0716, upper whisker = 1.2674/9.2033, 95th percentile = 1.2372/9.1966), Myeloid Cell (min = 0.0104/0.0032, max = 1.8229/19.2280, median = 0.4934/1.0720, mean = 0.5847/1.4393, lower whisker = 0.0104/0.0032, upper whisker = 1.2988/3.9222, 95th percentile = 1.3739/3.7889), Mast Cell (min = 0.0732/0.1191, max = 0.5190/10.0436, median = 0.2052/0.4905, mean = 0.2128/0.5765, lower whisker = 0.0732/0.1191, upper whisker = 0.4121/1.0925, 95th percentile = 0.3820/1.0138), Eccrine Gland (min = 0.0012/0.0017, max = 0.0873/0.3837, median = 0.0158/0.0232, mean = 0.0201/0.0424, lower whisker = 0.0012/0.0017, upper whisker = 0.0588/0.0966, 95th percentile = 0.0487/0.1275) for *CXCL1*; Keratinocyte-1 (min = 0.0054/0.0436, max = 2.8499/1.1675, median = 0.3033/0.2212, mean = 0.3706/0.2717, lower whisker = 0.0054/0.0436, upper whisker = 1.0234/0.6486, 95th percentile = 0.9001/0.5699), Keratinocyte-2 (min = 0.0231/0.0119, max = 1.8105/20.4381, median = 0.2590/0.4055, mean = 0.2933/0.5699, lower whisker = 0.0231/0.0119, upper whisker = 0.6936/1.3328, 95th percentile = 0.6271/1.5084), Keratinocyte-3 (min = 0.0607/0.0281, max = 1.2440/5.5007, median = 0.6806/0.4410, mean = 0.6481/0.5593, lower whisker = 0.0607/0.0281, upper whisker = 1.2440/1.2734, 95th percentile = 1.0515/1.4100), Fibroblast (min = 0.0366/0.0617, max = 5.4288/27.4380, median = 0.3219/1.1753, mean = 0.4593/1.9295, lower whisker = 0.0366/0.0617, upper whisker = 1.0833/5.3657, 95th percentile = 1.2122/6.0698), T-Cell (min=0.0062/0.0065, max = 0.5589/6.4854, median = 0.0340/0.0861, mean = 0.0474/0.1274, lower whisker = 0.0062/0.0065, upper whisker = 0.1099/0.2845, 95th percentile = 0.1260/0.3394), Endothelial Cell (min = 0.0032/0.0052, max = 1.2288/2.6625, median = 0.0688/0.0777, mean = 0.1035/0.1456, lower whisker = 0.0032/0.0052, upper whisker = 0.2650/0.3627, 95th percentile = 0.2890/0.5058), Smooth Muscle (min = 0.0034/0.0055, max = 2.2183/4.5404, median = 0.0847/0.1834, mean = 0.1913/0.3389, lower whisker = 0.0034/0.0055, upper whisker = 0.5502/0.8210, 95th percentile = 0.7248/1.1467), Melanocyte and Nerve Cell (min = 0.0194/0.0716, max = 1.8830/16.2340, median = 0.4136/2.6095, mean = 0.4975/3.4355, lower whiske r = 0.0194/0.0716, upper whisker = 1.2674/9.2033, 95th percentile = 1.2372/9.1966), Myeloid Cell (min = 0.0104/0.0032, max = 1.8229/19.2280, median = 0.4934/1.0720, mean = 0.5847/1.4393, lower whisker = 0.0104/0.0032, upper whisker = 1.2988/3.9222, 95th percentile = 1.3739/3.7889), Mast Cell (min = 0.0732/0.1191, max = 0.5190/10.0436, median = 0.2052/0.4905, mean = 0.2128/0.5765, lower whisker = 0.0732/0.1191, upper whisker = 0.4121/1.0925, 95th percentile = 0.3820/1.0138), Eccrine Gland (min = 0.0012/0.0017, max = 0.0873/0.3837, median = 0.0158/0.0232, mean = 0.0201/0.0424, lower whisker = 0.0012/0.0017, upper whisker = 0.0588/0.0966, 95th percentile = 0.0487/0.1275) for *CXCL2*; Keratinocyte-1 (min = 0.0054/0.0436, max = 2.8499/1.1675, median = 0.3033/0.2212, mean = 0.3706/0.2717, lower whisker = 0.0054/0.0436, upper whisker = 1.0234/0.6486, 95th percentile = 0.9001/0.5699), Keratinocyte-2 (min = 0.0231/0.0119, max = 1.8105/20.4381, median = 0.2590/0.4055, mean = 0.2933/0.5699, lower whisker = 0.0231/0.0119, upper whisker = 0.6936/1.3328, 95th percentile = 0.6271/1.5084), Keratinocyte-3 (min = 0.0607/0.0281, max = 1.2440/5.5007, median=0.6806/0.4410, mean = 0.6481/0.5593, lower whisker = 0.0607/0.0281, upper whisker = 1.2440/1.2734, 95th percentile = 1.0515/1.4100), Fibroblast (min = 0.0366/0.0617, max = 5.4288/27.4380, median = 0.3219/1.1753, mean = 0.4593/1.9295, lower whisker = 0.0366/0.0617, upper whisker = 1.0833/5.3657, 95th percentile = 1.2122/6.0698), T-Cell (min = 0.0062/0.0065, max = 0.5589/6.4854, median = 0.0340/0.0861, mean = 0.0474/0.1274, lower whisker = 0.0062/0.0065, upper whisker = 0.1099/0.2845, 95th percentile = 0.1260/0.3394), Endothelial Cell (min = 0.0032/0.0052, max = 1.2288/2.6625, median = 0.0688/0.0777, mean = 0.1035/0.1456, lower whisker = 0.0032/0.0052, upper whisker = 0.2650/0.3627, 95th percentile = 0.2890/0.5058), Smooth Muscle (min = 0.0034/0.0055, max = 2.2183/4.5404, median = 0.0847/0.1834, mean = 0.1913/0.3389, lower whisker = 0.0034/0.0055, upper whisker = 0.5502/0.8210, 95th percentile = 0.7248/1.1467), Melanocyte and Nerve Cell (min = 0.0194/0.0716, max = 1.8830/16.2340, median = 0.4136/2.6095, mean = 0.4975/3.4355, lower whisker = 0.0194/0.0716, upper whisker = 1.2674/9.2033, 95th percentile = 1.2372/9.1966), Myeloid Cell (min = 0.0104/0.0032, max = 1.8229/19.2280, median = 0.4934/1.0720, mean = 0.5847/1.4393, lower whisker = 0.0104/0.0032, upper whisker = 1.2988/3.9222, 95th percentile = 1.3739/3.7889), Mast Cell (min = 0.0732/0.1191, max = 0.5190/10.0436, median = 0.2052/0.4905, mean = 0.2128/0.5765, lower whisker = 0.0732/0.1191, upper whisker = 0.4121/1.0925, 95th percentile = 0.3820/1.0138), Eccrine Gland (min = 0.0012/0.0017, max = 0.0873/0.3837, median = 0.0158/0.0232, mean = 0.0201/0.0424, lower whisker = 0.0012/0.0017, upper whisker = 0.0588/0.0966, 95th percentile = 0.0487/0.1275) for *CXCL5*, and re-clustering of annotated endothelial cell subset (**H**). Each dot shown in the chart represents the measurement from 1 experimental subject and shown as mean ± S.E. $^{*}p < 0.05$; $^{**}p < 0.01$; $^{***}p < 0.001$; $^{****}p < 0.0001$.

© The Author(s)
