# Supplementary material for: Skin-derived G-CSF activates pathological granulopoiesis upon psoriasis
Source: EMBO Mol Med. 2026 Jun 16;18(7):2777–807. doi: 10.1038/s44321-026-00456-y (PMC13365241; doi:10.1038/s44321-026-00456-y)
Supplement: Supplementary file 2 — Peer Review File [file 44321_2026_456_MOESM2_ESM.pdf]

EMBO Molecular Medicine

Skin-derived G-CSF activates pathological granulopoiesis upon psoriasis

Tomson Kosasih, Tatsuya Morishima, Sohyeon Lee, Jungyeon Yoon, Kanako Wakahashi, Pilhan Kim, Aiko Sada, and Hitoshi Takizawa

Corresponding author: Hitoshi Takizawa (htakizawa@kumamoto-u.ac.jp)

Review Timeline:

Transferred from Review Commons:	24th Dec 25
Editorial Decision:	7th Jan 26
Revision Received:	1st Apr 26
Editorial Decision:	4th May 26
Revision Received:	14th May 26
Accepted:	20th May 26

Editor: Lise Roth

Transaction Report:

This manuscript was transferred to EMBO Molecular Medicine following peer review at Review Commons.

(Note: With the exception of the correction of typographical or spelling errors that could be a source of ambiguity, letters and reports are not edited. Depending on transfer agreements, referee reports obtained elsewhere may or may not be included in this compilation. Referee reports are anonymous unless the Referee chooses to sign their reports.)

Review #1

1. Evidence, reproducibility and clarity:

Evidence, reproducibility and clarity (Required)

Summary:

A role of neutrophils in psoriasis pathogenesis has been highlighted by several past studies; however, how the neutrophils are recruited to the affected skin has not been fully understood. The work by Kosasih et al. tackles a relevant question and has investigated the effect of psoriatic skin inflammation on BM myelopoiesis. Using an IMQ-induced acute psoriasis mouse model, the authors derive 3 major conclusions: (1) skin ECs secrete G-CSF into circulation in response to psoriatic stress, (2) skin EC-derived G-CSF stimulates emergency granulopoiesis, and (3) skin EC-derived G-CSF induces infiltration and accumulation of pathogenic neutrophils in the affected skin. The authors provide many pieces of interesting data, but most of them remain correlative and insufficient to support the conclusions. Many of the experiments were performed in a small number of samples or mice (mostly with n=3), leaving the story still preliminary.

Major comments:

1. First, the authors have not convincingly shown that skin cells, or more specifically skin ECs, are a major source of circulating G-CSF in the psoriasis model as stated in the title and abstract. The data in Figure 4 show selective upregulation of Csf3 gene in skin ECs and their ability to secrete G-CSF upon IMQ treatment in vitro. However, the provided data do not address to what degree the skin EC-derived G-CSF contributes to the elevated level of circulating G-CSF. Additional experiments to selectively deplete G-CSF in skin ECs, or at least in skin cells of the affected site, are warranted to support the authors' claim. Does intradermal injection of G-CSF neutralizing antibody into the psoriatic skin reduce circulating levels of G-CSF?
2. Another concern is insufficient demonstration of G-CSF-mediated emergency granulopoiesis in the psoriasis model. All data in Figure 5 were obtained from experiments with only n=3, and adding more replicates, in particular to those in Figure 5B, which show quite some variation in MPP numbers, is recommended. The relatively small reduction of BM granulocyte numbers (Figure 5C) compared to greater depletion of circulating granulocytes (Figure S5A) raises the possibility that it is the mobilization effect rather than granulopoiesis-stimulating effect that skin-derived G-CSF exerts to promote supply of circulating neutrophils that eventually infiltrate into the affected skin. This could also explain the negligible effect of IL-1blockade (Figure S4), which selectively shut off myelopoiesis-stimulating effect of IL-1 (Pietras et al. Nat Cell Biol 2016, PMID: 27111842). Are the HSPCs in the psoriasis model more cycling? Do they show myeloid-skewed differentiation when cultured ex vivo or upon transplantation?
3. The authors' claim that skin-derived G-CSF "induces" neutrophil infiltration warrants further clarification. Alternative explanation is that the upregulated neutrophil-attracting chemokines (Figure S1D) could induce infiltration, whereas G-CSF increase the number of neutrophils to circulate in the vessels near the psoriatic skin. This notion seems supported elsewhere (Moos et al. J Invest Dermatol. 2019, PMID: 30684554). Can the infiltration be inhibited by systemically

injecting neutralizing antibody of their receptor, CXCR2?
4. It remains unclear how skin-derived G-CSF accumulates pathogenic neutrophils. The authors state "pathogenic granulopoiesis," but are the circulating neutrophils in the psoriatic mice already "pathogenic" or do they acquire pathogenic phenotype after cutaneous infiltration? Additional RNA-seq to compare circulating and infiltrated neutrophils would answer this question.
5. In addition, how the accumulated pathogenic neutrophils exacerbate the psoriatic changes remains obscure. Although the authors have attempted to correlate Il17a gene expression in infiltrated neutrophils with psoriatic skin changes, the data do not address to what degree it contributes to cutaneous IL-17A protein levels. The data that cutaneous neutrophil depletion leads to subtle decrease in skin IL-17A expression (Figure 2H) rather supports alternative possibilities. For instance, as indicated elsewhere, IL-17A cutaneous tone could be enhanced by neutrophil-mediated augmentation of Th17 or gamma/delta T cell function (Lambert et al. J Invest Dermatol. 2019, PMID: 30528823). Does neutrophil depletion or G-CSF neutralization alter cell numbers or function of cutaneous Th17 and gamma/delta T cells?
6. Finally, as the above conclusions rely solely on the IMQ-induced acute psoriasis model, it would be informative if they could be derived from another psoriasis model. IMQ is known to induce unintended systemic inflammation due to grooming-associated ingestion (Gangwar et al. J Invest Dermatol. 2022, PMID: 34953514), and "pathological crosstalk between skin and BM in psoriatic inflammation" could be strengthened by an intradermal injection model.

Minor comments:

7. Figure 1E shows multiple elongated Ly6G+ structures in d0-2 control and d0 IMQ skins that do not appear to be neutrophils.
8. In Figure 2C, the bottom GSEA seems to be showing type II IFN response, not type I IFN, according to the text.
9. For the BM analysis in Figures 3, 5, S3, and S5, it would be informative if BM cellularity and numbers of committed myeloid progenitors (e.g., GMPs) are shown.
10. In Figures 6B, in which cluster of human skin cells IL-17A expression would be enriched?

2. Significance:

Significance (Required)

Although quite a few studies have reported various examples of emergency myelopoiesis (Swann et al. Nat Rev Immunol. 2024, PMID: 38467802), there is limited evidence on its occurrence and involvement in locally restricted disease, such as periodontitis (Li et al. Cell 2022, PMID: 35483374; 35483374). As an HSC biologist, I see this study is conceptually interesting as it could extend the above concept to psoriasis, a non-infectious, local inflammatory disease in the skin, and describes a potential causal link between skin-derived G-CSF and emergency myelopoiesis. That said, as detailed in the first section, the conclusions, especially that related to emergency myelopoiesis driven by skin-derived G-CSF, need to be more convincingly supported before taking its value. The findings offer additional understanding of how psoriasis is developed in concert with aberrant hematopoiesis and will be relevant to those working in the field of dermatology, immunology, and hematology.

3. How much time do you estimate the authors will need to complete the suggested revisions:

Estimated time to Complete Revisions (Required)

(Decision Recommendation)

Between 3 and 6 months

4. *Review Commons* values the work of reviewers and encourages them to get credit for their work. Select 'Yes' below to register your reviewing activity at [Web of Science Reviewer Recognition Service](#) (formerly Publons); note that the content of your review will not be visible on Web of Science.

Web of Science Reviewer Recognition

Yes

Review #2

1. Evidence, reproducibility and clarity:

Evidence, reproducibility and clarity (Required)

General assessment

This is a well-written and carefully executed study that identifies skin-derived G-CSF as a key driver of pathological emergency granulopoiesis in an imiquimod-induced mouse model of psoriasis. The authors convincingly show that endothelial cells are the dominant source of G-CSF in inflamed skin, and that this cytokine mediates systemic hematopoietic skewing and neutrophil accumulation, ultimately aggravating psoriatic pathology. The e-analysis of human transcriptomic datasets strengthens the translational relevance of the findings. Overall, the conclusions are well supported by the data.
However, several mechanistically relevant aspects remain underexplored, particularly regarding the functional state of psoriatic neutrophils and the human data integration. Addressing these points would substantially enhance the impact of the study.

Major points

1. Interpretation of neutrophil transcriptomic changes (Figure 2)

The RNA-seq analysis reveals substantial downregulation of several canonical pro-inflammatory pathways in neutrophils from psoriatic skin, including IL-6, IL-1, and type II interferon signaling. The authors should discuss the functional relevance of this unexpected transcriptional repression. For example, does this indicate a shift toward specialized effector functions rather than classical cytokine responsiveness? More importantly, the most striking transcriptional change is the upregulation of NADPH oxidase-related genes (e.g., Nox1, Nox3, Nox4, Enox2). This suggests an oxidative-stress-driven pathogenic mechanism, potentially more relevant than IL-17A production. Yet this aspect is not explored in the manuscript. Assessing ROS levels or oxidative neutrophil effector functions in this model would considerably strengthen the mechanistic link.

Conversely, although IL-17A is upregulated in neutrophils, neutrophil depletion reduces total Il17a expression in skin only partially. This indicates that neutrophils are unlikely to be the dominant IL-17A source in the lesion. The authors' focus on neutrophil-derived IL-17A therefore seems overstated. A more rigorous assessment-e.g., conditional deletion of Il17a specifically in neutrophils-would be required to establish its true contribution. Taken together, the data suggest that oxidative programs, rather than IL-17A production, may represent the principal pathogenic axis downstream of neutrophils, and this deserves deeper discussion.
2. Human data reanalysis (Figure 6):

The re-analysis of bulk and single-cell RNA-seq datasets is valuable but incomplete.

Several mechanistically relevant questions could be addressed with the available data:

2.1. GM-CSF (CSF2) is also strongly upregulated in psoriatic lesions (bulk RNA-seq). It would be informative to determine whether endothelial cells also express CSF2 in the scRNA-seq dataset, as this would suggest coordinated regulation of myeloid-supporting cytokines.

2.2. Myeloid cell subsets should be examined more closely. A comparison of human myeloid transcriptomes with the mouse neutrophil RNA-seq
would clarify whether similar IL-17A-related or NADPH oxidase-related signatures occur in human disease. In particular, which cell types express IL17A in human lesions?

2.3. Chemokine production should be attributed to specific cell types. Bulk RNA-seq confirms strong induction of CXCL1, CXCL2, CXCL5, but the scRNA-seq dataset allows determining whether these chemokines originate from endothelial cells or other stromal/immune populations. This information is important for defining whether endothelial cells coordinate both neutrophil recruitment and granulopoiesis. Addressing these points would make the human-mouse comparison substantially stronger.

Minor points

1. Line 319: the text likely refers to Figure S4, not S3.
2. Line 338: "psoriatic" is misspelled.

Referees cross-commenting

I agree with Reviewer 1 that the contribution of EC-derived G-CSF to circulating G-CSF levels and to emergency myelopoiesis requires additional genetic or neutralization experiments to be fully established.

2. Significance:

Significance (Required)

The study is solid and potentially impactful, particularly for audiences working in inflammation and hematopoiesis, as it uncovers a cross-organ mechanism linking skin-derived G-CSF to emergency granulopoiesis in psoriasis. My expertise lies in inflammation and hematopoiesis, and from this perspective several essential mechanistic issues remain insufficiently addressed. In particular, the neutrophil transcriptomic data highlight strong induction of NADPH oxidase-related pathways, which appears more biologically meaningful than the modest Il17a upregulation emphasized by the authors. Likewise, the human RNA-seq reanalyses leave open key questions regarding CSF2 expression, myeloid heterogeneity, and chemokine cellular sources. These issues affect the strength and interpretation of the central claims. For these reasons, I recommend major revision before the manuscript can be considered further.

3. How much time do you estimate the authors will need to complete the suggested revisions:

Estimated time to Complete Revisions (Required)

(Decision Recommendation)

Between 1 and 3 months

4. *Review Commons* values the work of reviewers and encourages them to get credit for their work. Select 'Yes' below to register your reviewing activity at Web of Science Reviewer Recognition Service (formerly Publons); note that the content of your review will not be visible on Web of Science.

Web of Science Reviewer Recognition

Yes

Review #3

1. Evidence, reproducibility and clarity:

Evidence, reproducibility and clarity (Required)

Summary:

Provide a short summary of the findings and key conclusions (including methodology and model system(s) where appropriate).

Study titled: "Skin-derived G-CSF activates pathological granulopoiesis upon psoriasis" by Kosasih and Takizawa.
Paper show establishment of psoriasis model in C57BL/6 mice. They focus on neutrophils infiltration following the Imiquimod cream induction. Importantly, authors show that the induction of psoriasis in the skin cause a robust enhancement of granulopoiesis in the bone marrow. Mechanistically, G-CSF is produced in the skin, especially by endothelial cells. Blocking of G-CSF gained clear inhibition of psoriatic pathology. They further add human data showing that patient with psoriasis have more neutrophils and more G-CSF in their skin endothelial cells.

Parts of the study are simply in line with previous knowledge (e.g.- neutrophils infiltration into psoriatic skin, IL17a). authors show some data that largely confirm the model used.
Major discovery: skin endothelial cells are secreting G-CSF that induce granulopoiesis in the bone-marrow. This is a conceptual advancement of this study: psoriatic skin not only recruit neutrophils from the blood, but also enhance the generation of new neutrophils in the bone-marrow. That a major- psoriasis at the level of the model used must not be considered as a confined-pathology. It affect systematically, and might also benefit new systemic treatments. There are plenty of follow-up experiments to pursue now, so it is critical to publish this finding and let many laboratories to know of this new direction. I expect this study to attract high interest and many citations.

Major comments:

- Are the key conclusions convincing?

Yes. The study has excellent data, with good quantification, and very solid support for the discovery and interpretations.
- Should the authors qualify some of their claims as preliminary or speculative, or remove them altogether?

No. It is very solid.
- Would additional experiments be essential to support the claims of the paper? Request additional experiments only where necessary for the paper as it is, and do not ask authors to open new lines of experimentation.

Such a discovery clearly opens many options, and it is fascinating to suggest additional experiments for future studies. It is a complete study, best to publish as-is and let many to read and proceed with this new concept.
- Are the suggested experiments realistic in terms of time and resources? It would help if you could add an estimated cost and time investment for substantial experiments.

N/A - I suggest no additional experiments at this point. Get it published and see how many will follow this new direction!
- Are the data and the methods presented in such a way that they can be reproduced?

Yes.
- Are the experiments adequately replicated, and is the statistical analysis adequate?

Yes. The data are of very high quality.

Minor comments:

- Specific experimental issues that are easily addressable.

None. It is good as-is. One may always suggest minor things- but this one is better published so many laboratories may rush for this new direction. I think it will be interesting studying some long-term impacts, and changes not only of neutrophils but also of other innate cells, such as DCs, Macrophages, and Eosinophils - so it is best to let laboratories that focus on these cells know of the discovery and pursue independent studies.
- Are prior studies referenced appropriately?

Yes. I may suggest adding a recent review by Park and Jung, 2024, https://doi.org/10.1016/j.mam.2024.101306 to cover current concepts of innate immunity in psoriasis.
- Are the text and figures clear and accurate?

Yes.
- Do you have suggestions that would help the authors improve the presentation of their data and conclusions?

It is all good. May add graphical-abstract.

2. Significance:

Significance (Required)

- Describe the nature and significance of the advance (e.g., conceptual, technical, clinical) for the field.

Conceptual advancement - discovery of a major impact of psoriasis on bone-marrow granulopoiesis. Explicit finding of endothelial-cells G-CSF as a major communication moiety.

- Place the work in the context of the existing literature (provide references, where appropriate).
Psoriasis is extensively studied, a good recent reference-
https://doi.org/10.1016/j.mam.2024.101306

Neutrophil recruitment and IL17A are well established.
G-CSF of endothelial cells brings the conceptual advancement- psoriasis at the level induced by IMQ develops local pathology, but is tightly linked to systemic changes. The impact on bone-marrow granulopoiesis may have many implications. So far, it was largely considered that chronic inflammation may affect hematopoiesis, but this study reveals an acute and specific communication between skin and bone marrow. The neutrophils are not only recruited from blood- they are made anew, so the disease is enhanced significantly!
This discovery led to a novel basic understanding and suggests novel therapeutic options.
- State what audience might be interested in and influenced by the reported findings.

Dermatologist, immunologist, haematologist - this one goes for a broad audience.
- Define your field of expertise with a few keywords to help the authors contextualize your point of view. Indicate if there are any parts of the paper that you do not have sufficient expertise to evaluate.

Immunology and hematology. I am not an expert of dermatology.

3. How much time do you estimate the authors will need to complete the suggested revisions:

Estimated time to Complete Revisions (Required)

(Decision Recommendation)

Less than 1 month

4. *Review Commons* values the work of reviewers and encourages them to get credit for their work. Select 'Yes' below to register your reviewing activity at Web of Science Reviewer Recognition Service (formerly Publons); note that the content of your review will not be visible on Web of Science.

Web of Science Reviewer Recognition

Yes

Revision Plan

Manuscript number: RC-2025-03286
Corresponding author(s): Hitoshi Takizawa

[The "revision plan" should delineate the revisions that authors intend to carry out in response to the points raised by the referees. It also provides the authors with the opportunity to explain their view of the paper and of the referee reports.
The document is important for the editors of affiliate journals when they make a first decision on the transferred manuscript. It will also be useful to readers of the reprint and help them to obtain a balanced view of the paper.

If you wish to submit a full revision, please use our "[Full Revision]" template. ***It is important to use the appropriate template to clearly inform the editors of your intentions.****]*

- General Statements [optional]

This section is optional. Insert here any general statements you wish to make about the goal of the study or about the reviews.

We appreciated all the valuable and constructive comments from all 2 reviewers except the reviewer #3 who we felt shows an enthusiasm to endorse publication without further revision. We would like to propose the following seven critical experiments to address their comments which we believe would help strengthening our manuscript:

No	Experiment to address reviewers' comments
1	Intradermal injection of G-CSF neutralizing antibody (anti-G-CSF/IgG-isotype) into IMQ-treated mice.
2	Adding more replicate for HPSC analysis in mice treated with anti-G-CSF/IgG-isotype antibody and IMQ.
3	Flow-cytometric analysis of HSPCs cycling status in bone marrow of Vas/IMQ-treated mice.
4	Transplantation of HSPC fraction of BM in Vas/IMQ-treated mice.
5	Immunophenotyping of Th17 and gamma/delta Tcells in the skin of neutrophil-depleted mice (anti-Ly6G/IgG-isotype)
6	Flow-cytometry-based ROS measurement in the neutrophils from Vas/IMQ-treated mice
7	RNA-seq of peripheral blood-circulating and skin-infiltrating neutrophils from both Vas/IMQ-treated mice

- Description of the planned revisions

Reviewer #1

1. First, the authors have not convincingly shown that skin cells, or more specifically skin ECs, are a major source of circulating G-CSF in the psoriasis model as stated in the title and abstract. The data in Figure 4 show selective upregulation of Csf3 gene in skin ECs and their ability to

secrete G-CSF upon IMQ treatment in vitro. However, the provided data do not address to what degree the skin EC-derived G-CSF contributes to the elevated level of circulating G-CSF. Additional experiments to selectively deplete G-CSF in skin ECs, or at least in skin cells of the affected site, are warranted to support the authors' claim. Does intradermal injection of G-CSF neutralizing antibody into the psoriatic skin reduce circulating levels of G-CSF?

Author's response:
Thank you for reviewer's comment. We agree with the Reviewer#1 that it is important to directly block G-CSF to the skin via intradermal injection and measure the G-CSF level in the serum afterwards. Therefore, we will perform intradermal injection of IgG-isotype or anti-G-CSF antibody into the IMQ-induced psoriatic mice.

We also tried to extrapolate G-CSF amount based on *in vitro* and *in vivo* data below:

- **G-CSF amount produced by endothelial cell *in vitro***

ID	Content	Unit	DMSO	IMQ
A	G-CSF conc. (Fig. 4G)	pg/mL	11.6457	17.7792
B	Volume medium used/well	mL	0.05	0.05
C	Total G-CSF in media/well (AxB)	pg	0.5823	0.8898
D	Number of cells plated/well	cells	60000	60000
E	G-CSF/cell (AxB/D)	pg/cell	9.705 e^{-6}	14.820 e^{-6}

- **Extrapolation of G-CSF production in the skin region treated by Vas or IMQ *in vivo***

ID	Content	Unit	Vas	IMQ
F	Number of EC cells/treated skin ($4cm^2$)	cells	0.7753 e^{6}	1.0501 e^{6}
G	Estimated G-CSF/treated skin (ExF)	pg	7.524	15.5625

- **Calculation of G-CSF amount in a mouse whole body**
  - Assumption
    - Whole blood is 6% of TBW *(Jackson lab, https://www.jax.org/news-and-insights/2005/october/how-much-blood-can-i-take-from-a-mouse-without-endangering-its-health)*
    - Serum is 50% whole blood *(CDC, chrome-extension://efaidnbmnnnibpcajpcglclefindmkaj/https://www.cdc.gov/anthrax/pdf/serology-collection-shipping-final-jul2016-508.pdf#:~:text=10%20mL%20of%20whole%20blood%20will%20approximately,the%20minimum%20acceptable%20volume%20needed%20for%20testing.)*
    - Blood density is 1.057 g/mL (Schneck, Biomedical engineering, 2015)
    - Adult B6 mouse weight used in our study is 20g on average (Bachmanov AA, et al., Behav Genet, 2002)
- serum volume in an adult mouse

ID	Content	Unit	Measurement
H	Mouse body weight	g	20
I	Blood weight to total body weight	%	6-8

J	Blood weight to total body weight (HxI)	g	1.2-1.6
K	Whole blood Density	g/mL	1.057
L	Whole blood volume (JxK)	mL	1.1353-1.6912
M	Serum volume to total blood volume	%	50
N	Serum volume (LxM)	mL	0.5677-0.8456

- Absolute G-CSF amount in 0.5677-0.8456 mL serum of whole body

Time after treatment	Vas (pg/mL)	IMQ (pg/mL)	Vas (pg)	IMQ (pg)	Difference between in vitro and in vivo data (fold)
16h	425.5	1249.2	241.6-359.8	709.2-1056.3	32.1-67.9
2d	156.8	2294.8	89.0-132.6	1302.8-1940.5	11.8-124.7
4d	202.6	1301.7	115.0-171.3	739.0-1100.7	15.3-70.7

***Conclusion on comparison in G-CSF amount between In vitro- vs in vivo* data-based extrapolation**

The range of difference in G-CSF amount between *in vitro* and *in vivo* data-based extrapolation is ~12-125 fold, which is surprisingly small for us, given that *in vitro* culture of endothelial cells do not often recapitulate *in vivo* situation and therefore, do not optimize the condition for G-CSF production. However, this may suggest that skin-EC is not the only source of G-CSF production in response to TLR activation but also other sources that contribute to G-CSF level *in vivo*. We will discuss this.

2. Another concern is insufficient demonstration of G-CSF-mediated emergency granulopoiesis in the psoriasis model. All data in Figure 5 were obtained from experiments with only n=3, and adding more replicates, in particular to those in Figure 5B, which show quite some variation in MPP numbers, is recommended. The relatively small reduction of BM granulocyte numbers (Figure 5C) compared to greater depletion of circulating granulocytes (Figure S5A) raises the possibility that it is the mobilization effect rather than granulopoiesis-stimulating effect that skin-derived G-CSF exerts to promote supply of circulating neutrophils that eventually infiltrate into the affected skin. This could also explain the negligible effect of IL-1blockade (Figure S4), which selectively shut off myelopoiesis-stimulating effect of IL-1 (Pietras et al. Nat Cell Biol 2016, PMID: 27111842). Are the HSPCs in the psoriasis model more cycling? Do they show myeloid-skewed differentiation when cultured ex vivo or upon transplantation?

Author's response: Thank you for these critical comments. We agree to do the following experiments to address them:
1) HSPCs quantification in Figure 5 especially the MPPs will be added with more replicates.
2) We will assess cycling status of HSPCs by flow cytometric analysis of Ki67and Propidium Iodide to characterize G0, G1 and G2/M cell cycle phase.

3) To test myeloid-skewed differentiation, Lin^{-} $c\text{-}Kit^{+}$ $Sca\text{-}1^{+}$ cells containing HSPCs will be isolated from bone marrow of Vas/IMQ-treated mice and transplanted into lethally irradiated syngeneic mice.

4. It remains unclear how skin-derived G-CSF accumulates pathogenic neutrophils. The authors state "pathogenic granulopoiesis," but are the circulating neutrophils in the psoriatic mice already "pathogenic" or do they acquire pathogenic phenotype after cutaneous infiltration? Additional RNA-seq to compare circulating and infiltrated neutrophils would answer this question.

Author's response: We appreciate this valuable comment. We will perform RNA-seq with the peripheral blood-circulating neutrophils ($CD45^{+}$ $CD11b^{+}$ $Ly6G^{+}$ $Ly6C^{mid}$) versus skin-infiltrating neutrophils from both Vas/IMQ mice.

5. In addition, how the accumulated pathogenic neutrophils exacerbate the psoriatic changes remains obscure. Although the authors have attempted to correlate Il17a gene expression in infiltrated neutrophils with psoriatic skin changes, the data do not address to what degree it contributes to cutaneous IL-17A protein levels. The data that cutaneous neutrophil depletion leads to subtle decrease in skin IL-17A expression (Figure 2H) rather supports alternative possibilities. For instance, as indicated elsewhere, IL-17A cutaneous tone could be enhanced by neutrophil-mediated augmentation of Th17 or gamma/delta T cell function (Lambert et al. J Invest Dermatol. 2019, PMID: 30528823). Does neutrophil depletion or G-CSF neutralization alter cell numbers or function of cutaneous Th17 and gamma/delta T cells?

Author's response: Thank you for this insightful comment. To better understand the relative contribution of neutrophils to the cutaneous IL-17A tone in the psoriatic skin, we will perform flowcytometric analysis of Th17 and gamma/delta T cells which are widely known as the major source of IL-17 in psoriatic skin of IMQ-induced mice following injection of isotype-matched or anti-Ly6G antibody.

6. Finally, as the above conclusions rely solely on the IMQ-induced acute psoriasis model, it would be informative if they could be derived from another psoriasis model. IMQ is known to induce unintended systemic inflammation due to grooming-associated ingestion (Gangwar et al. J Invest Dermatol. 2022, PMID: 34953514), and "pathological crosstalk between skin and BM in psoriatic inflammation" could be strengthened by an intradermal injection model.

Author's response: We appreciate the reviewer for bringing this important point. Regarding the systemic inflammation upon psoriasis, the above-cited study reported increased IFN-B expression in the intestines of IMQ-ingested animal (Grine L et al. Sci Rep. 2016, PMID: 26818707 in Gangwar et al. J Invest Dermatol. 2022, PMID: 34953514). We examined several pro-inflammatory cytokines including IFN-β, IFN-γ, and IL-6 and in contrast, found no systemic increase in all these cytokines, except for IFN-γ downregulation (**Explanation Figure 1**), which suggests no evidence of grooming-associated ingestion.

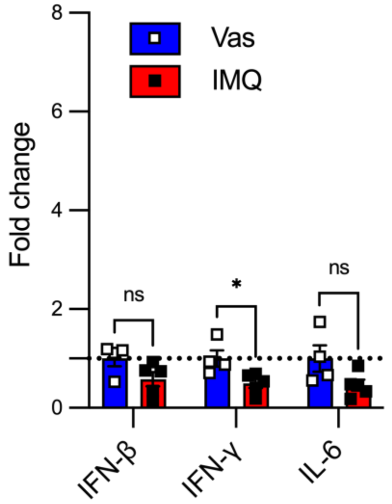

Explanation Figure 1. Level of inflammatory cytokines in the mouse serum

We also examined the *Csf3* expression across several distinctively located tissues which showed a selective upregulation in the skin (**Figure 4C**), suggesting a skin-restricted perturbation. In addition, one study showed that IMQ-ingestion didn't alter number of gut injury-associated CXCR3^{+} macrophages nor did it aggravate skin inflammation (Pinget et al. Cell Reports. 2022, PMID: 35977500). Together, these findings support that IMQ-induced psoriasis by topical cutaneous application used in our study elicit a local inflammation but not systemic inflammation.

The authors, however, realize that testing alternative psoriasis model such as intradermal injection of IL-23 (Chan et al. J Exp Med. 2006, PMID: 17074928) will strengthen the skin-local insults within the psoriasis model employed, and should be tested in the future.

9. For the BM analysis in Figures 3, 5, S3, and S5, it would be informative if BM cellularity and numbers of committed myeloid progenitors (e.g., GMPs) are shown.

Author's response: We appreciate that Reviewer#1 bring up this point. We examined the kinetics of the bone marrow cellularity and GMPs across 4 days of psoriasis induction in mice. The bone marrow cell number was lowered along that span with lowermost count at 2 days. Consistent to the BM-cellularity, the GMP number was also lowered about one-third in the first 2 days of psoriasis. This kinetic is consistent with the previous report showing a rapid reduction of GMPs in the bone marrow within 2 days following systemic G-CSF administration driven emergency granulopoiesis (Hirai et al. Nat. Immunol. 2006, PMID: 16751774). From 2 days to 4 days, the GMP number rapidly increased to slightly above basal number (**Explanation Figure 2**). This timely coordinated expansion suggests a significant supply of GMPs from the differentiating upstream myeloid progenitors (**Figure 3B**).

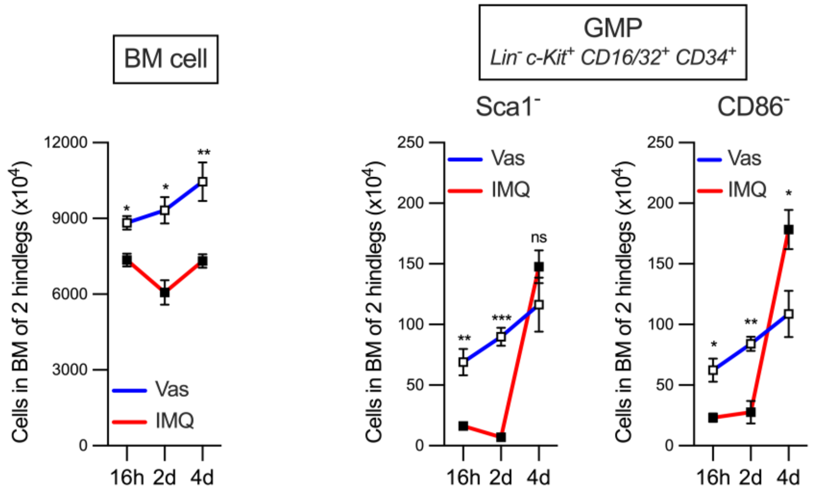

Explanation Figure 2. Number of bone marrow and GMP cells in the mice treated with topical Vas/IMQ.

When the psoriatic mice with elevated G-CSF is injected with anti-G-CSF or IgG-isotype antibody, the bone marrow cellularity and GMP numbers at 4 days were (**Explanation Figure 3**). Firstly, as psoriasis reduced bone marrow cellularity (**Explanation Figure 2**), the unchanged number after anti-G-CSF injection indicates that administration of 10µg/day for 4 days does not significantly affect mobilization of psoriatic bone marrow cells. Secondly, the similar GMP numbers at 4 days psoriasis is plausibly due to snapshot analysis when it has already in the numerical recovery period (**Explanation Figure 2**). Importantly, the notion that anti-G-CSF injection to psoriatic mice reduced granulocytes in the bone marrow, peripheral blood, and skin suggesting G-CSF as a key mediator in psoriatic driven emergency granulopoiesis on top of unlikely case of ineffective anti-G-CSF treatment.

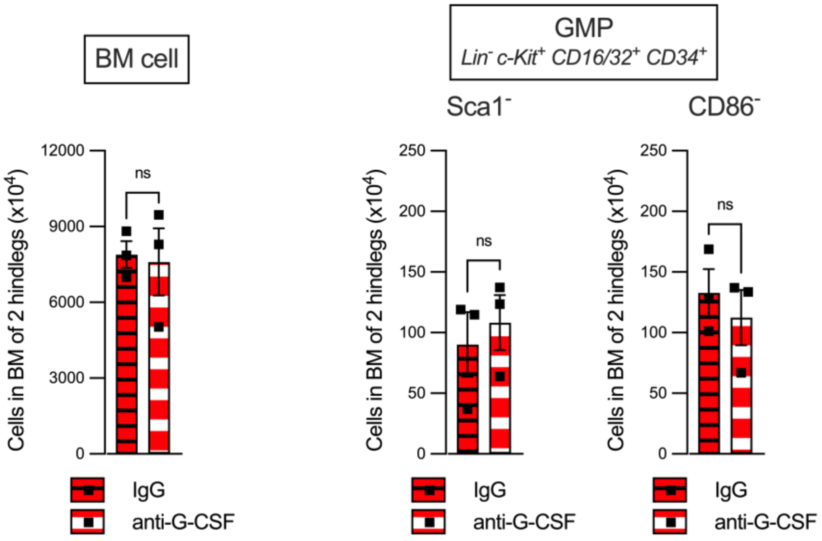

Explanation Figure 3. Bone marrow and GMP cell number in the IMQ-treated mice injected with anti-G-CSF or IgG-isotype at 4 days post-treatment.

Taken together, these data suggest a G-CSF mediated emergency granulopoiesis occurrence in the IMQ-induced psoriasis. We will put these data into a revised Figure.

10. In Figures 6B, in which cluster of human skin cells IL-17A expression would be enriched?
Author's response: Thank you for this important point. The IL-17A expression is found in the T-cell cluster (**Explanation Figure 4**). We also expected to see IL-17A contribution from other cell subset(s), in particular neutrophil. However, due to the fragile nature of neutrophils and thereby, technical difficulty to get their sequencing reads, this dataset (GSE173706) doesn't contain neutrophils, but rather monocytes, macrophages, and dendritic cells among the myeloid subset (**Explanation Figure 5**). With this, it leaves open the question on what potential contribution of IL-17A produced by neutrophils is in human psoriasis (Reich et al. Exp. Dermatol. 2015, PMID: 25828362).

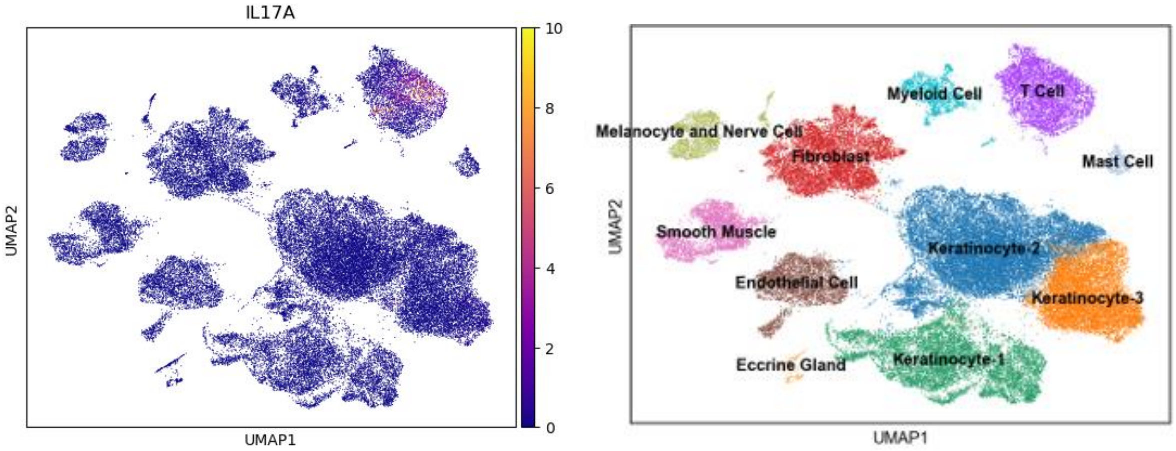

Explanation Figure 4. *IL17A* expressional map in human skin scRNAseq dataset (GSE173706)

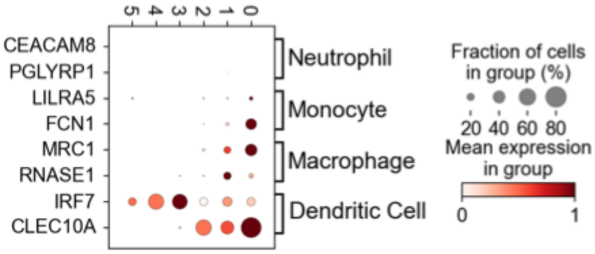

Explanation Figure 5. Cell annotation of myeloid subset (GSE173706)

Reviewer#2
1. Interpretation of neutrophil transcriptomic changes (Figure 2)
The RNA-seq analysis reveals substantial downregulation of several canonical pro inflammatory pathways in neutrophils from psoriatic skin, including IL-6, IL-1, and type II interferon signaling. The authors should discuss the functional relevance of this unexpected transcriptional repression. For example, does this indicate a shift toward specialized effector functions rather

than classical cytokine responsiveness? More importantly, the most striking transcriptional change is the upregulation of NADPH oxidase-related genes (e.g., Nox1, Nox3, Nox4, Enox2). This suggests an oxidative stress-driven pathogenic mechanism, potentially more relevant than IL-17A production. Yet this aspect is not explored in the manuscript. Assessing ROS levels or oxidative neutrophil effector functions in this model would considerably strengthen the mechanistic link. Conversely, although IL-17A is upregulated in neutrophils, neutrophil depletion reduces total Il17a expression in skin only partially. This indicates that neutrophils are unlikely to be the dominant IL-17A source in the lesion. The authors' focus on neutrophil-derived IL 17A therefore seems overstated. A more rigorous assessment-e.g., conditional deletion of Il17a specifically in neutrophils-would be required to establish its true contribution. Taken together, the data suggest that oxidative programs, rather than IL-17A production, may represent the principal pathogenic axis downstream of neutrophils, and this deserves deeper discussion.

Author’s response: Thank you for raising this valuable views. We have agreed to address these critical points by the following approaches:
1) To address the changes in NADPH oxidase-related gene signature, we will measure ROS production in the neutrophils from skin and peripheral blood with DHR123.
2) Responding to the IL17A contribution by neutrophils, we will flow cytometrically assess the Th17 and gamma/delta T cell population in the skin of psoriatic mice treated with anti-Ly6G or isotype-matched antibody as was suggested by Reviewer#1.
3) We will discuss downregulation of the canonical pro inflammatory and IL-17 pathways in the psoriatic neutrophils in the discussion.

2. Human data reanalysis (Figure 6):
The re-analysis of bulk and single-cell RNA-seq datasets is valuable but incomplete. Several mechanistically relevant questions could be addressed with the available data:
2.1. GM-CSF (CSF2) is also strongly upregulated in psoriatic lesions (bulk RNA-seq). It would be informative to determine whether endothelial cells also express CSF2 in the scRNA-seq dataset, as this would suggest coordinated regulation of myeloid-supporting cytokines.
2.2. Myeloid cell subsets should be examined more closely. A comparison of human myeloid transcriptomes with the mouse neutrophil RNA-seq would clarify whether similar IL-17A-related or NADPH oxidase-related signatures occur in human disease. In particular, which cell types express IL17A in human lesions?
2.3. Chemokine production should be attributed to specific cell types. Bulk RNA-seq confirms strong induction of CXCL1, CXCL2, CXCL5, but the scRNA-seq dataset allows determining whether these chemokines originate from endothelial cells or other stromal/immune populations. This information is important for defining whether endothelial cells coordinate both neutrophil recruitment and granulopoiesis.
Addressing these points would make the human-mouse comparison substantially stronger.

Author’s response: Thank you for pointing these important issues. By reanalyzing the dataset, we found several points regarding the comments, as follows:

2.1) *CSF2* is expressed by T-cell cluster in the human skin dataset (**Explanation Figure 4**), in agreement with previous murine study (Hartwig et al. Cell Reports. 2018, PMID: 30590032). We will add this data in the revised manuscript.

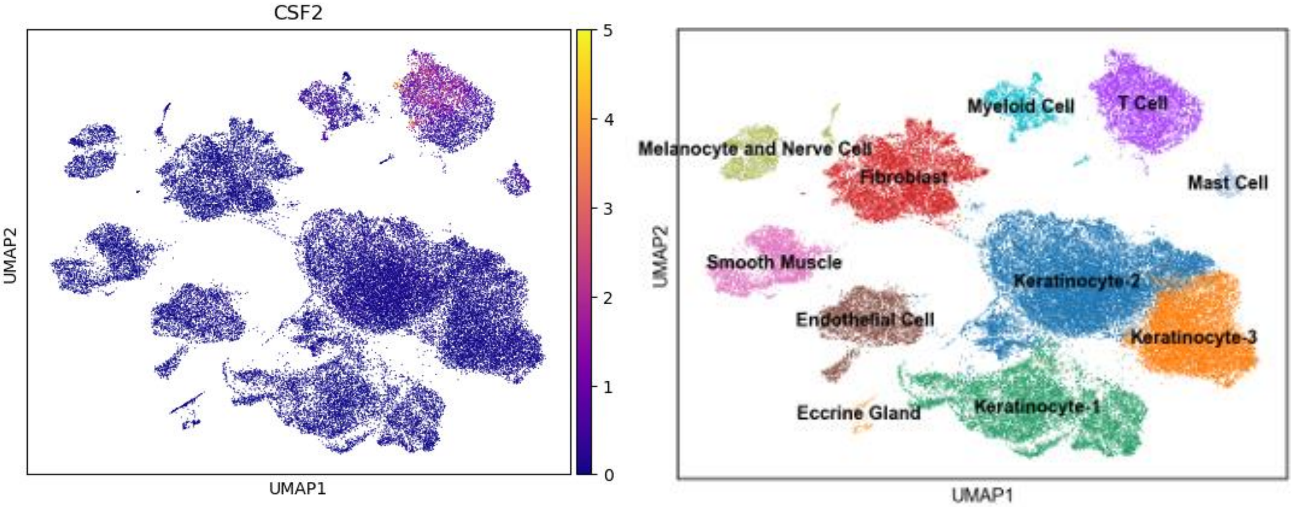

Explanation Figure 6. CSF2 expressional map in human skin scRNAseq dataset (GSE 173706)

2.2) In line with point#10 from Reviewer#1, the dataset clearly shows T-cell cluster as the main *IL17A* source (**Explanation Figure 4 above**). The dataset, however, doesn't contain phenotypic neutrophils *(CEACAM (CD66b*) and *PGLYRP1*) but monocytes, macrophages, and dendritic cells (**Explanation Figure 5 above**). This loss was probably due to a technical limitation given the difficulty in capturing sequencing reads from fragile neutrophils. Therefore, it is no longer possible to reanalyze IL-17 expression in the absence of neutrophils in the datapool.

2.3) Reanalysis of CXCLs in the human scRNAseq dataset (GSE173706) clarified their secretion dynamics and cellular sources under normal and psoriatic condition. In normal skin, all examined cell subsets show only low *CXCLs* expression. In contrast, psoriatic skin exhibits significant *CXCLs* upregulation with distinct cell subsets clearly showing dramatic upregulation, potentially being the major *CXCLs* source. *CXCL1* is markedly upregulated in fibroblasts, myeloid cells, and melanocyte and nerve cells. *CXCL2* is strikingly upregulated to myeloid cells, while *CXCL5* is hugely increased in fibroblasts, myeloid cells, and mast cells (**Explanation Figure 7**). Taken together, these results suggest that *CXCLs* upregulation in the psoriatic skin is coordinatively executed by both stromal and immune compartments. Of note, the endothelial cells show minimal changes in *CXCL*s expression, even downregulate *CXCL2* in psoriasis, indicating that they are unlikely to be the major contributor to CXCL-mediated neutrophil recruitment.

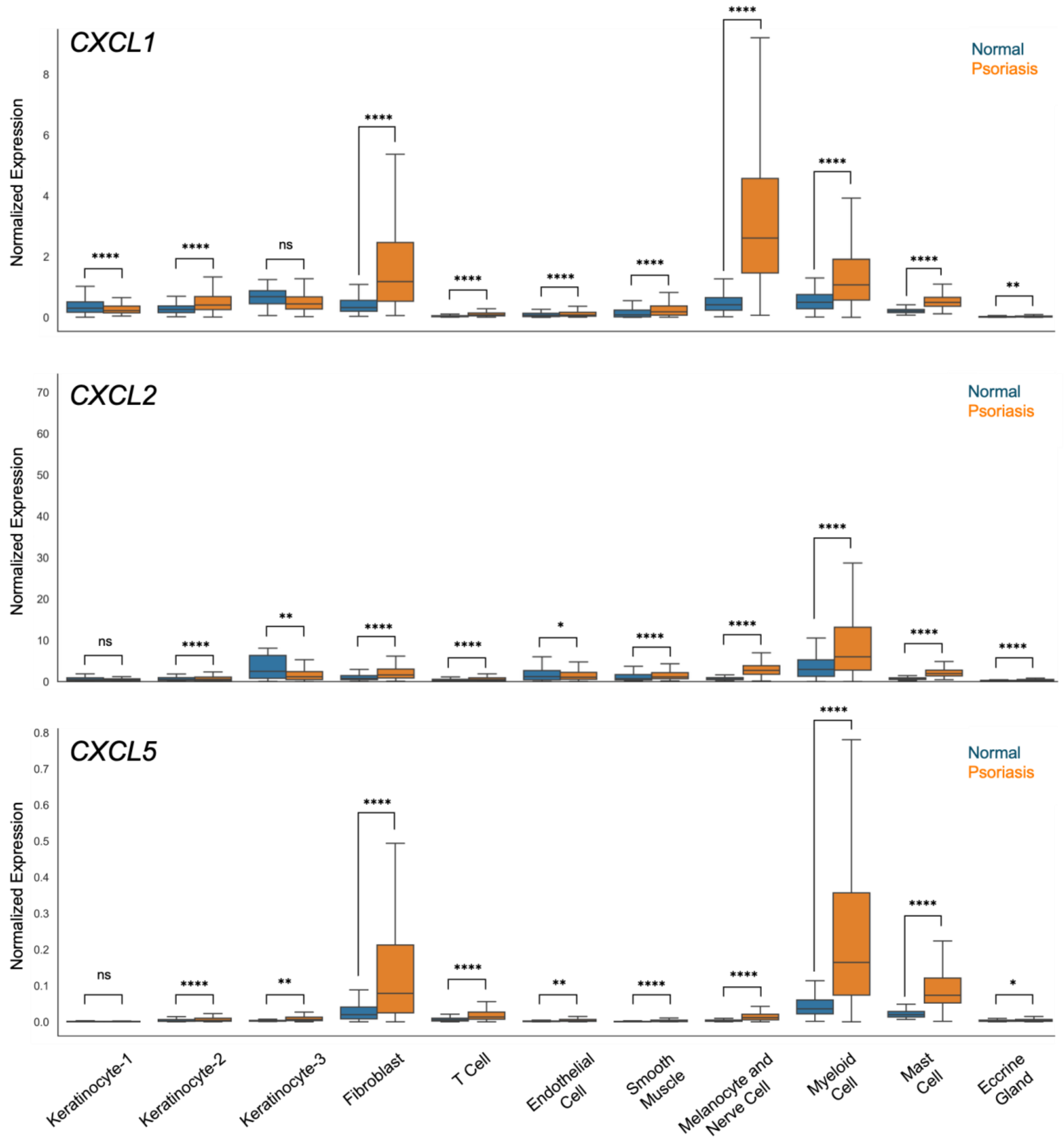

Explanation Figure 7. *CXCL1, CXCL2, CXCL5* expression in normal and psoriatic human skin from scRNAseq data (GSE173706)

Referees cross-commenting

I agree with Reviewer 1 that the contribution of EC-derived G-CSF to circulating G-CSF levels and to emergency myelopoiesis requires additional genetic or neutralization experiments to be fully established.

Author's response: We appreciate that Reviewer#2 raised this key point. In addition to examining the serum G-CSF upon intradermal anti-G-CSF administration in point#1 from Reviewer#1 above, we will also examine the emergency myelopoiesis signs *in vivo*.

Reviewer#3

- Place the work in the context of the existing literature (provide references, where appropriate). Psoriasis is extensively studied, a good recent reference- https://doi.org/10.1016/j.mam.2024.101306

Author's response: Thank you for Reviewer#3's suggestion. The referenced study highlights the current paradigm that largely focus on skin-restricted mechanism and overlook potential cross-organ interaction in the psoriasis inflammation. Our findings provide a new insight into the skin-bone marrow crosstalk in the disease context. In addition, the suggested reference underscores the key roles of diverse innate immune cells including neutrophils, eosinophils, dendritic cells, etc. which is fundamental for our study and might also guide future exploration of additional innate cell subsets beyond neutrophils. We will therefore include the mentioned reference to our revised manuscript.

- Do you have suggestions that would help the authors improve the presentation of their data and conclusions?

It is all good. May add graphical-abstract.

Author's response: Thank you for the reviewer's input, we agree that a graphical-abstract will help the readers more clearly grasp the key messages of our manuscript. We will include it in the revised manuscript.

- Description of the revisions that have already been incorporated in the transferred manuscript

Please insert a point-by-point reply describing the revisions that were already carried out and included in the transferred manuscript. If no revisions have been carried out yet, please leave this section empty.

Reviewer#1

Minor comments

7. Figure 1E shows multiple elongated Ly6G+ structures in d0-2 control and d0 IMQ skins that do not appear to be neutrophils.

Author's response: We appreciate the Reviewer#1 pointing this issue. As mentioned by the Reviewer#1, the elongated structures detected in the intravital microscopy are not neutrophils, but autofluorescence from the skin bulge regions (Wun et al. J Invest Dermatol. 2005, PMID: 15816847). We have eliminated these unspecific signals from the transformation and quantification (**Figure 1F, S1G, and S1H**). We will also add an explanatory sentence in Materials and Methods section "Of note, *the fluorescent signal with elongated structures resembling hair bulge were autofluorescence and thus removed from further analysis.*" to be more precise about our methods.

8. In Figure 2C, the bottom GSEA seems to be showing type II IFN response, not type I IFN, according to the text.

Author's response: Thank you for the comment, we will correct this misspelling.

Reviewer#2

Minor points

1. Line 319: the text likely refers to Figure S4, not S3.

Author's response: Thank you, we will correct the nomenclature.

2. Line 338: "psoriatic" is misspelled.

Author's response: Thank you, we will change this to "psoriatic".

- Description of analyses that authors prefer not to carry out

Please include a point-by-point response explaining why some of the requested data or additional analyses might not be necessary or cannot be provided within the scope of a revision. This can be due to time or resource limitations or in case of disagreement about the necessity of such additional data given the scope of the study. Please leave empty if not applicable.

Reviewer#1

3. The authors' claim that skin-derived G-CSF "induces" neutrophil infiltration warrants further clarification. Alternative explanation is that the upregulated neutrophil-attracting chemokines (Figure S1D) could induce infiltration, whereas G-CSF increase the number of neutrophils to circulate in the vessels near the psoriatic skin. This notion seems supported elsewhere (Moos et al. J Invest Dermatol. 2019, PMID: 30684554). Can the infiltration be inhibited by systemically injecting neutralizing antibody of their receptor, CXCR2?

Author's response: The manuscript focuses on the skin-derived G-CSF function as a long-distance signal for emergency granulopoiesis in the bone marrow upon psoriasis, not the chemoattractant property of it. The sentence of interest is "*We found that upon psoriasis*

induction, skin-resident endothelial cells are activated to produce G-CSF which activates emergency granulopoiesis in bone marrow and induces cutaneous ***infiltration and*** *accumulation of neutrophil that are functionally inflammatory."* in line 28-30. In agreement with point #2 from Reviewer#2, the fact that neutrophil recruitment factors (*CXCL1*, *CXCL2*, and *CXCL5*) were upregulated in psoriatic skin (**Figure S1D**), suggesting a CXCL-mediated neutrophil recruitment. The sentence of concern need to be changed to "*We found that upon psoriasis induction, skin-resident endothelial cells are activated to produce G-CSF which activates emergency granulopoiesis in bone marrow,* ***leading to*** *cutaneous accumulation of neutrophil that are functionally inflammatory."*. This revised sentence has omitted the proposal that G-CSF directly dictates neutrophils mobilization to the skin, which is not the key message of the study. Therefore, we found that the CXCR2 (CXCLs receptor) blockade experiment may be of the benefit of future studies.

Reviewer#3
Major comments:
- Should the authors qualify some of their claims as preliminary or speculative, or remove them altogether?
No. It is very solid.

Author's response: We appreciate the reviewer's view that the claims in our paper are solid.

- Would additional experiments be essential to support the claims of the paper? Request additional experiments only where necessary for the paper as it is, and do not ask authors to open new lines of experimentation.
Such a discovery clearly opens many options, and it is fascinating to suggest additional experiments for future studies. It is a complete study, best to publish as-is and let many to read and proceed with this new concept.

Author's response: We thank the reviewer for noting that the current experimental evidence is complete that no additional experiments are necessary at this stage. We agree that the discovery opens prospective directions for future studies.

- Are the suggested experiments realistic in terms of time and resources? It would help if you could add an estimated cost and time investment for substantial experiments.
N/A - I suggest no additional experiments at this point. Get it published and see how many will follow this new direction!

Author's response: We thank the reviewer for recognizing that the experimental data has been sufficient to be a foundation for the future research.

- Are the data and the methods presented in such a way that they can be reproduced?
Yes.

Author’s response: We thank the reviewer for recognizing that our methods are reproducible.

- Are the experiments adequately replicated, and is the statistical analysis adequate?
Yes. The data are of very high quality.

Author’s response: We are grateful that the reviewer view our replication strategy and statistical analysis are of a high quality.

Minor comments:
- Specific experimental issues that are easily addressable.
None. It is good as-is. One may always suggest minor things- but this one is better published so many laboratories may rush for this new direction. I think it will be interesting studying some long-term impacts, and changes not only of neutrophils but also of other innate cells, such as DCs, Macrophages, and Eosinophils - so it is best to let laboratories that focus on these cells know of the discovery and pursue independent studies.

Author’s response: We appreciate the reviewer’s assessment that our paper is already well set for the community to explore the newly proposed direction.

- Are the text and figures clear and accurate?
Yes.

Author’s response: We thank the reviewer’s evaluation. We have ensured that the text and figures in our manuscript are clear and accurate. Once again, we thank the reviewer for the encouraging and constructive appraisal. We are pleased that the reviewer find the manuscript has already been strong and suitable for publication.

7th Jan 2026

Dear Prof. Takizawa,

Thank you for the submission of your manuscript to our editorial offices, and please accept my apologies for the delay in getting back to you, which is due to the reduced activity of our office at this time of the year. I have now had the opportunity to read your manuscript, together with the referees' reports and your rebuttal letter, and to discuss them with the other members of our editorial team.

We agree that the study fits the scope of the journal, and we appreciate that you are willing to address most of the referees' concerns experimentally (the remaining points should be addressed by adequate discussion). We thus encourage you to submit a revised version of your manuscript, including the modifications and revisions described in your point-by-point letter.

If you would like to discuss further the points raised by the referees, I am available to do so via email or video. Let me know if you are interested in this option.

Acceptance of the manuscript will entail a second round of review. EMBO Molecular Medicine encourages a single round of revision only and therefore, acceptance or rejection of the manuscript will depend on the completeness of your responses included in the next, final version of the manuscript. For this reason, and to save you from any frustrations in the end, I would strongly advise against returning an incomplete revision.

When preparing your revised manuscript, please refer to our guidelines: https://link.springer.com/journal/44321/submission-guidelines#cms-Revised-submissions.

We require:

1) A .docx formatted version of the manuscript text (including legends for main figures, EV figures and tables). Please make sure that the changes are highlighted to be clearly visible.

2) Individual production quality figure files as .eps, .tif, .jpg (one file per figure). For guidance, download the 'Figure Guide PDF': https://media.springernature.com/original/springer-cms/rest/v1/content/27825798/data/v1.

3) A .docx formatted letter INCLUDING the reviewers' reports and your detailed point-by-point responses to their comments. As part of the EMBO Press transparent editorial process, the point-by-point response is part of the Review Process File (RPF), which will be published alongside your paper.

4) A complete author checklist, which you can download from our author guidelines. Please insert information in the checklist that is also reflected in the manuscript. The completed author checklist will also be part of the RPF.

5) Please note that all corresponding authors are required to supply an ORCID ID for their name upon submission of a revised manuscript.

6) It is mandatory to include a 'Data Availability' section after the Materials and Methods. Before submitting your revision, primary datasets produced in this study need to be deposited in an appropriate public database, and the accession numbers and database listed under 'Data Availability'. Please remember to provide a reviewer password if the datasets are not yet public.

In case you have no data that requires deposition in a public database, please state so in this section. Note that the Data Availability Section is restricted to new primary data that are part of this study.

7) For data quantification: please specify the name of the statistical test used to generate error bars and P values, the number (n) of independent experiments (specify technical or biological replicates) underlying each data point and the test used to calculate p-values in each figure legend. The figure legends should contain a basic description of n, P and the test applied. Graphs must include a description of the bars and the error bars (s.d., s.e.m.).

8) At EMBO Press we ask authors to provide source data for the main manuscript figures. You will receive a separate email with instructions for providing source data with your revised manuscript, including how to upload and organize the files.

9) Our journal encourages inclusion of *data citations in the reference list* to directly cite datasets that were re-used and obtained from public databases. Data citations in the article text are distinct from normal bibliographical citations and should directly link to the database records from which the data can be accessed. In the main text, data citations are formatted as follows: "Data ref: Smith et al, 2001" or "Data ref: NCBI Sequence Read Archive PRJNA342805, 2017". In the Reference list, data citations must be labeled with "[DATASET]". A data reference must provide the database name, accession

number/identifiers and a resolvable link to the landing page from which the data can be accessed at the end of the reference.

10) We replaced Supplementary Information with Expanded View (EV) Figures and Tables that are collapsible/expandable online. A maximum of 5 EV Figures can be typeset. EV Figures should be cited as 'Figure EV1, Figure EV2" etc... in the text and their respective legends should be included in the main text after the legends of regular figures.

- For the figures that you do NOT wish to display as Expanded View figures, they should be bundled together with their legends in a single PDF file called *Appendix*, which should start with a short Table of Content. Appendix figures should be referred to in the main text as: "Appendix Figure S1, Appendix Figure S2" etc.

- Additional Tables/Datasets should be labeled and referred to as Table EV1, Dataset EV1, etc. Legends have to be provided in a separate tab in case of .xls files. Alternatively, the legend can be supplied as a separate text file (README) and zipped together with the Table/Dataset file.

11) The paper explained: EMBO Molecular Medicine articles are accompanied by a summary of the articles to emphasize the major findings in the paper and their medical implications for the non-specialist reader. Please provide a draft summary of your article highlighting
- the medical issue you are addressing,
- the results obtained and
- their clinical impact.

This may be edited to ensure that readers understand the significance and context of the research. Please refer to any of our published articles for an example.

12) Author contributions: You will be asked to provide CRediT (Contributor Role Taxonomy) terms in the submission system. These replace a narrative author contribution section in the manuscript.

13) A Conflict of Interest statement should be provided in the main text.

14) Every published paper includes a 'Synopsis' to further enhance discoverability. Synopses are displayed on the journal webpage and are freely accessible to all readers. They include a short stand first (maximum of 300 characters, including space) as well as 2-5 one-sentences bullet points that summarizes the paper. Please write the bullet points to summarize the key NEW findings. They should be designed to be complementary to the abstract - i.e. not repeat the same text. We encourage inclusion of key acronyms and quantitative information (maximum of 30 words / bullet point). Please use the passive voice. Please attach these in a separate file or send them by email, we will incorporate them accordingly.

Please also suggest a striking image or visual abstract to illustrate your article as a PNG file 550 px wide x 300-600 px high.

15) Include a Reagents and Tools Table as part of the Methods section, which can be downloaded from our author guidelines.

EMBO Molecular Medicine has a "scooping protection" policy, whereby similar findings that are published by others during review or revision are not a criterion for rejection. Should you decide to submit a revised version, I do ask that you get in touch after three months if you have not completed it, to update us on the status.
Molecular Medicine

I look forward to receiving your revised manuscript.

Yours sincerely,

Lise Roth

Lise Roth, PhD
Senior Editor
EMBO Molecular Medicine

***

Rev_Com_number: RC-2025-03286
New_manu_number: EMM-2025-23207-T
Corr_author: Takizawa
Title: Skin-derived G-CSF activates pathological granulopoiesis upon psoriasis

Full Revision

Manuscript number: RC-2025-03286 (EMM-2025-23207-T)
Corresponding author(s): Hitoshi Takizawa

[Please use this template only if the submitted manuscript should be considered by the affiliate journal as a full revision in response to the points raised by the reviewers.

If you wish to submit a preliminary revision with a revision plan, please use our "Revision Plan" template. ***It is important to use the appropriate template to clearly inform the editors of your intentions.****]*

1. General Statements [optional]

This section is optional. Insert here any general statements you wish to make about the goal of the study or about the reviews.

2. Point-by-point description of the revisions

This section is mandatory. Please insert a point-by-point reply describing the revisions that were already carried out and included in the transferred manuscript.

Reviewer #1
1. First, the authors have not convincingly shown that skin cells, or more specifically skin ECs, are a major source of circulating G-CSF in the psoriasis model as stated in the title and abstract. The data in Figure 4 show selective upregulation of Csf3 gene in skin ECs and their ability to secrete G-CSF upon IMQ treatment in vitro. However, the provided data do not address to what degree the skin EC-derived G-CSF contributes to the elevated level of circulating G-CSF. Additional experiments to selectively deplete G-CSF in skin ECs, or at least in skin cells of the affected site, are warranted to support the authors' claim. Does intradermal injection of G-CSF neutralizing antibody into the psoriatic skin reduce circulating levels of G-CSF?

Author’s response:
Thank you for this comment. As pointed out, we performed intradermal administration of anti-G-CSF antibody or IgG control into the 4 evenly spaced dorsal skin site with each receiving 2.5µg antibody in 50µL PBS per day for 4 consecutive days of IMQ application. At 16-18 hours after the last injection, we harvested the serum and measured its G-CSF level with ELISA. The results below (Explanation Figure 1) showed IMQ application alone significantly elevated G-CSF in the serum more than 10-fold compared to the basal level (Vas), which remained similar with both intraperitoneal and intradermal IgG injection. However, anti-G-CSF antibody administration by both intraperitoneal and intradermal route reduced the serum G-CSF close to the basal level (Explanation Figure 1). This result supports that the main source of G-CSF driving psoriasis is skin derived one. We have included this data in the new Appendix Figure S5A.

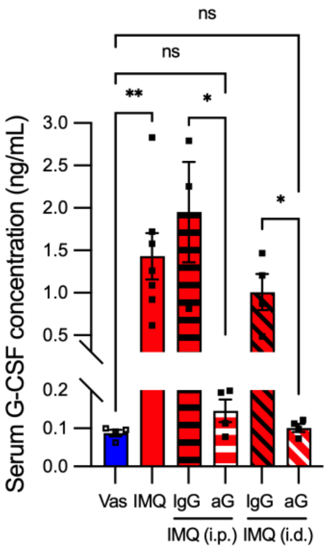

Explanation Figure 1. Serum G-CSF level in psoriasis mice treated with G-CSF. Vas: Vaseline, IMQ: Imiquimod, IgG: Immunoglobulin G isotype, aG: anti-G-CSF, i.p.: intraperitoneal, and i.d.: intradermal.

2. Another concern is insufficient demonstration of G-CSF-mediated emergency granulopoiesis in the psoriasis model. All data in Figure 5 were obtained from experiments with only n=3, and adding more replicates, in particular to those in Figure 5B, which show quite some variation in MPP numbers, is recommended. The relatively small reduction of BM granulocyte numbers (Figure 5C) compared to greater depletion of circulating granulocytes (Figure S5A) raises the possibility that it is the mobilization effect rather than granulopoiesis-stimulating effect that skin-derived G-CSF exerts to promote supply of circulating neutrophils that eventually infiltrate into the affected skin. This could also explain the negligible effect of IL-1blockade (Figure S4), which selectively shut off myelopoiesis-stimulating effect of IL-1 (Pietras et al. Nat Cell Biol 2016, PMID: 27111842). Are the HSPCs in the psoriasis model more cycling? Do they show myeloid-skewed differentiation when cultured ex vivo or upon transplantation?

Author's response:
Thank you for pointing out this critical point. We have added more experimental replicates to n=5 in each group which show a significant selective MPP3 reduction by about half in the psoriatic mice injected with anti-G-CSF antibody compared to IgG-isotype matched control (Explanation Figure 2). We have revised Figure 5B accordingly.

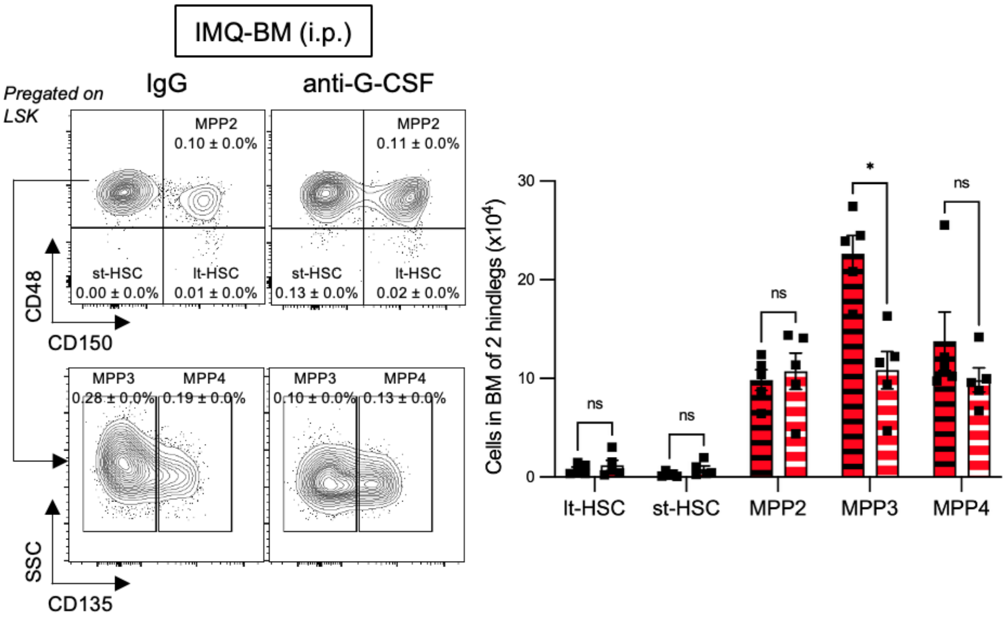

Explanation Figure 2. HSPC immunophenotyping of psoriatic mice treated with anti-G-CSF antibody.

Regarding the mobilization effect, we acknowledge that G-CSF has a dual role in promoting mobilization and granulopoiesis. These functions are mechanistically distinct. G-CSF-induced mobilization occurs indirectly through CXCL12-CXCR4 retention axis (Pettit et al. Nat Immunol 2002, PMID: 12068293), while its granulopoiesis effect is a direct consequence of G-CSFR signaling activation (Richards et al. Blood 2003, PMID: 12893769). Importantly, *Csf3*-deficient (KO) mice are chronically neutropenic, and have 20-30% of granulocyte number in steady state compared to wild type mice. Restoring the neutrophil with exogenous G-CSF administration took longer (~24h) than its maximum mobilization (~8h) (Lieschke et al. Blood 1994, PMID: 7521686, Stroncek et al. Transfusion 2002, PMID: 12084168), indicating that G-CSF-mediated granulopoiesis comes later than its mobilization modulation. Our kinetic analysis showed that while mature neutrophil slight reduced in BM between 16h and 2d (Fig. S3F), neutrophil infiltrating into skin increased, indicated of migration effect of G-CSF which increased (Fig. 4B). On top, there was a significant increase of both immature and mature neutrophil in BM at 4d which could explain an additional granulopoiesis effect. Since neutrophil number was significantly reduced in both skin and BM (less reduction than skin) at day 4 (Fig. 5) when G-CSF level was normalized by intradermal injection of anti-G-CSF antibody (Fig. S5A), we think skin-derived G-CSF drives both mobilization effect and granulopoiesis at day 4.

We, agree with Reviewer#1'comment that the stronger depletion observed in the circulation than in the bone marrow possibly indicate a contribution of impaired mobilization. To explore this possibility, we checked expression of egress- and retention-related receptor on bone marrow granulocytes from psoriatic mice without systemic G-CSF blockade (Explanation Figure 3). While we didn't observe a change in expression of CXCR4, CXCR2 expression was reduced in G-CSF-blocked psoriatic mice, indicating a less egress-primed granulocytes from this mouse (Explanation Figure 3). These findings suggest that G-CSF neutralization did not affect CXCR4-

mediated retention but rather reduced egress property of the granulocytes unexpectedly, proposing a minimal contribution of systemic G-CSF to the mobilization effect in psoriatic BM.

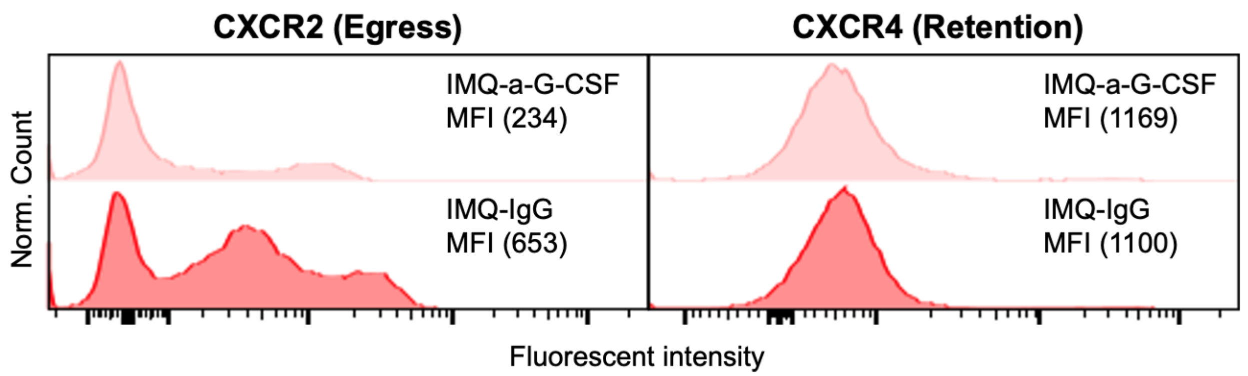

Explanation Figure 3. Mobilization receptors expression in psoriatic mice's bone marrow granulocytes (CD45^{+} CD11b^{+} Ly6G^{+}) treated with anti-G-CSF/IgG. IMQ: Imiquimod, a-G-CSF: anti-G-CSF.

To more definitively assess the role of IL-1 in BM-emergency granulopoiesis, we administered the IL1R inhibitor (Anakinra) at escalating doses from the dose previously used in Fig S4 (37µg/d) to 10-fold (370µg/d) and 100-fold (3700µg/d) across 4 days during IMQ application. Psoriasis condition (IMQ) augments granulopoiesis by enhanced both immature (Ly6G^{lo}) and mature (Ly6G^{hi}) granulocytes compared to steady state condition (Vas). However, IL1R inhibition had no dose-dependent effect on granulopoiesis. Consistent with the PBS injected psoriatic mice, both immature and mature granulocyte fractions remained at ~35% of the total CD45^{+} cells in the bone marrow across all psoriatic group, regardless of the dosages (Explanation Figure 4). Under these conditions, IL1R inhibition didn't seem to alter psoriatic driven granulopoiesis, suggesting that IL-1 signaling is not a major driver for granulopoiesis in psoriatic mice.

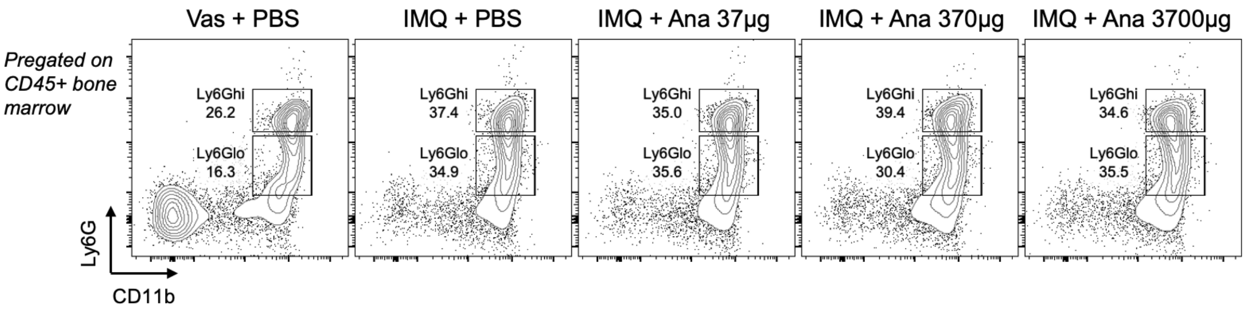

Explanation Figure 4. IL1R blockade with intraperitoneal administration of Anakinra to psoriatic mice. Vas: Vaseline, IMQ: Imiquimod, PBS: Phosphate Buffer Saline, Ana: Anakinra.

Next, we analyzed cell cycle status of the HSPC fractions both in normal (Vas) and psoriatic (IMQ) BM, and found that lt-HSC, MPP3, and MPP4 had a significant increase in its S/G2/M phase proportion (Explanation Figure 5), indicating psoriasis activate HSPC into proliferation.

We have added this data to Figure 3C and Appendix Figure S3D and edited the corresponding text.

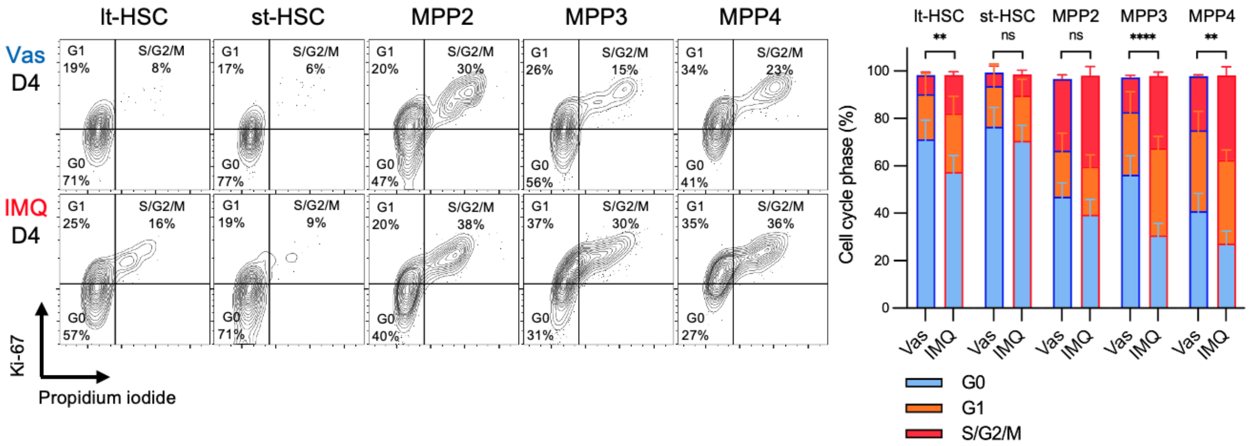

Explanation Figure 5. Cell cycle analysis of HSPC. Vas: Vaseline, IMQ: Imiquimod.

To address the lineage output skewing of HPSCs activated by psoriasis, we performed a transplantation assay with 700 LSK donor from Vas- or IMQ-treated BM ($CD45.1^+$) together with $3x10^5$ competitor BM cells ($CD45.1/2^+$) to the irradiated mice ($CD45.2^+$) (9.0 Gy) (Explanation Figure 6). We checked donor chimerism as well as lineage distribution in peripheral blood cells for up to 8 weeks after transplantation. We observed a significantly lower donor chimerism with no lineage skewing from psoriatic HSPCs compared with Vas control, (Explanation Figure 6). This, together with the previous cell cycle data, suggest psoriasis condition impairs HSPC function without changing lineage output, possibly through the forced proliferation (Takizawa et al. JEM 2011, PMID: 21300914, Essers et al. Nature 2009, PMID: 19212321). However, it remains unclear what is the underlying signal associated with psoriasis that induces this functional impairment and it is beyond the scope of this study. We have added this result to Figure 3D and Appendix Figure S3E.

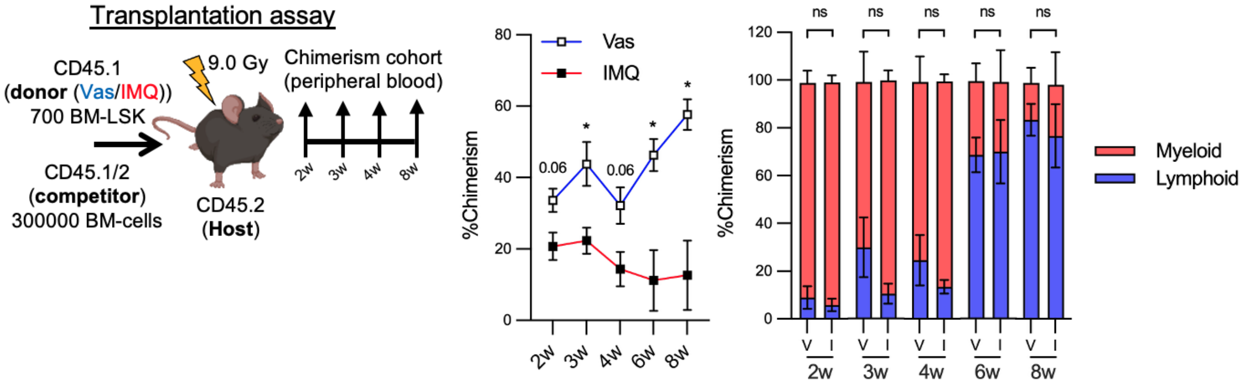

Explanation Figure 6. Transplantation assay of the HSPCs from Vas- or IMQ-treated BM. V: Vaseline, I: Imiquimod

In summary, psoriatic mice exhibit elevated G-CSF, increased immature granulocytes, and a more actively cycling HSPCs, all of which phenocopy the emergency granulopoiesis shown in other inflammation models (Bottcher et al. Blood 2014, PMID:24990886). We agree there is the possibility of G-CSF exerting mobilization effect in addition to the granulopoiesis driving action. However, the former effect could be less dominant than granulopoiesis effect at day 4, as we did not find a clear evidence that mobilization-regulating proteins are unchanged or rather decreased. Moreover, comparing to the experimental system which shows IL-1 driven myelopoiesis by administration of exogenous IL-1 (Pietras et al. Nat Cell Biol 2016, PMID: 27111842), our psoriatic model represents a broad multi-cytokine inflammation. This could explain the negligible IL-1 effect observed in our model which suggest that emergency granulopoiesis in psoriasis model is predominantly driven by G-CSF which may override IL-1-dependent effect.

3. The authors' claim that skin-derived G-CSF "induces" neutrophil infiltration warrants further clarification. Alternative explanation is that the upregulated neutrophil-attracting chemokines (Figure S1D) could induce infiltration, whereas G-CSF increase the number of neutrophils to circulate in the vessels near the psoriatic skin. This notion seems supported elsewhere (Moos et al. J Invest Dermatol. 2019, PMID: 30684554). Can the infiltration be inhibited by systemically injecting neutralizing antibody of their receptor, CXCR2?

Author’s response: We thank Reviewer#1 to bring this important point. CXCR2 antagonist administration in IMQ-treated psoriatic mice has been shown to significantly reduce granulocytes infiltration into the inflamed skin (Sumida et al. J. Immunol 2014, PMID: 24663678). Therefore, our study didn’t focus on the granulocyte mobilization dynamics. We agree that the original sentence may cause a misinterpretation as it may imply that G-CSF directly drives skin-selective granulocytes infiltration. To avoid this potential misunderstanding, we have revised the corresponding sentence in page 2, line 29 from “*We found that upon psoriasis induction, skin-resident endothelial cells are activated to produce G-CSF which activates emergency granulopoiesis in bone marrow and induces cutaneous **infiltration and** accumulation of neutrophil that are functionally inflammatory.”* to “*We found that upon psoriasis induction, skin-resident endothelial cells are activated to produce G-CSF which activates emergency granulopoiesis in bone marrow, **leading to** cutaneous accumulation of neutrophil that are functionally inflammatory.”*

4. It remains unclear how skin-derived G-CSF accumulates pathogenic neutrophils. The authors state "pathogenic granulopoiesis," but are the circulating neutrophils in the psoriatic mice already "pathogenic" or do they acquire pathogenic phenotype after cutaneous infiltration? Additional RNA-seq to compare circulating and infiltrated neutrophils would answer this question.

Author’s response: We thank for this valuable comment. To address this, we performed an additional RNA-seq of peripheral blood-derived neutrophils. The PCA analysis revealed that psoriatic (IMQ) neutrophils in skin and peripheral blood showed differential signature from

steady-state (Vas) neutrophils as they are distinctively clustered (Explanation Figure 7). We have added this to Figure 2A.

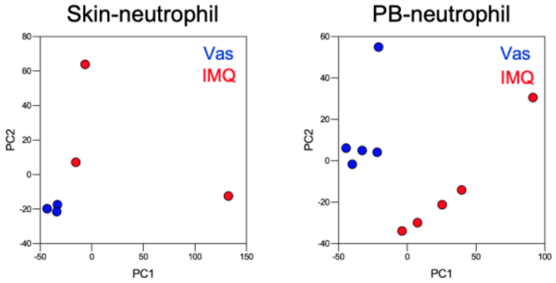

Explanation Figure 7. PCA analysis of neutrophils from skin and peripheral blood treated with Vas (Vaseline) or IMQ (Imiquimod).

GSEA analysis revealed a similar functional pattern observed in both skin and peripheral blood-derived neutrophil under psoriasis conditions, including reduction in antigen processing and presentation, and increased NADPH oxidase family, except classical IL-1 signaling which did not significantly change in peripheral blood-derived neutrophil (p=0.4520). Moreover, we also observed a skin neutrophil-restricted *Il17a* upregulation, suggesting a secondary functional acquisition in skin-infiltrating neutrophils upon psoriasis inflammation (Explanation Figure 8). We have added this to Figure 2B.

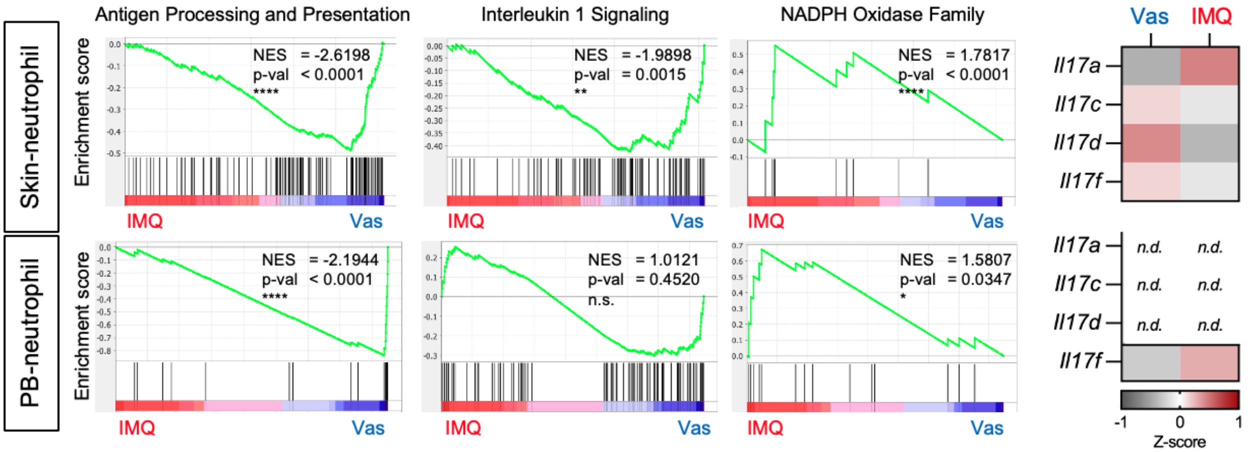

Explanation Figure 8. GSEA analysis and *Il17a* heatmap of skin and peripheral blood treated with Vas (Vaseline) or IMQ (Imiquimod).

Moreover, we observed a limited or inconsistent changes of gene signatures related to matrix metalloproteinase, defensin antimicrobial, IL-6 signaling, TNFA signaling, and type I IFN response in psoriatic skin- and peripheral blood-derived neutrophils. The type II IFN response was found downregulated in skin-neutrophils whereas unchanged in peripheral blood-derived neutrophil (Explanation Figure 9). We have transferred this figure to Appendix Figure S2A. Together, RNA-seq analysis suggests that the psoriatic-neutrophils have developed pathogenic signature such as oxidative burst before infiltrating the skin, and additionally acquired pro-

inflammatory program for instance Defensin Antimicrobial and IL-17A expression upon cutaneous entry.

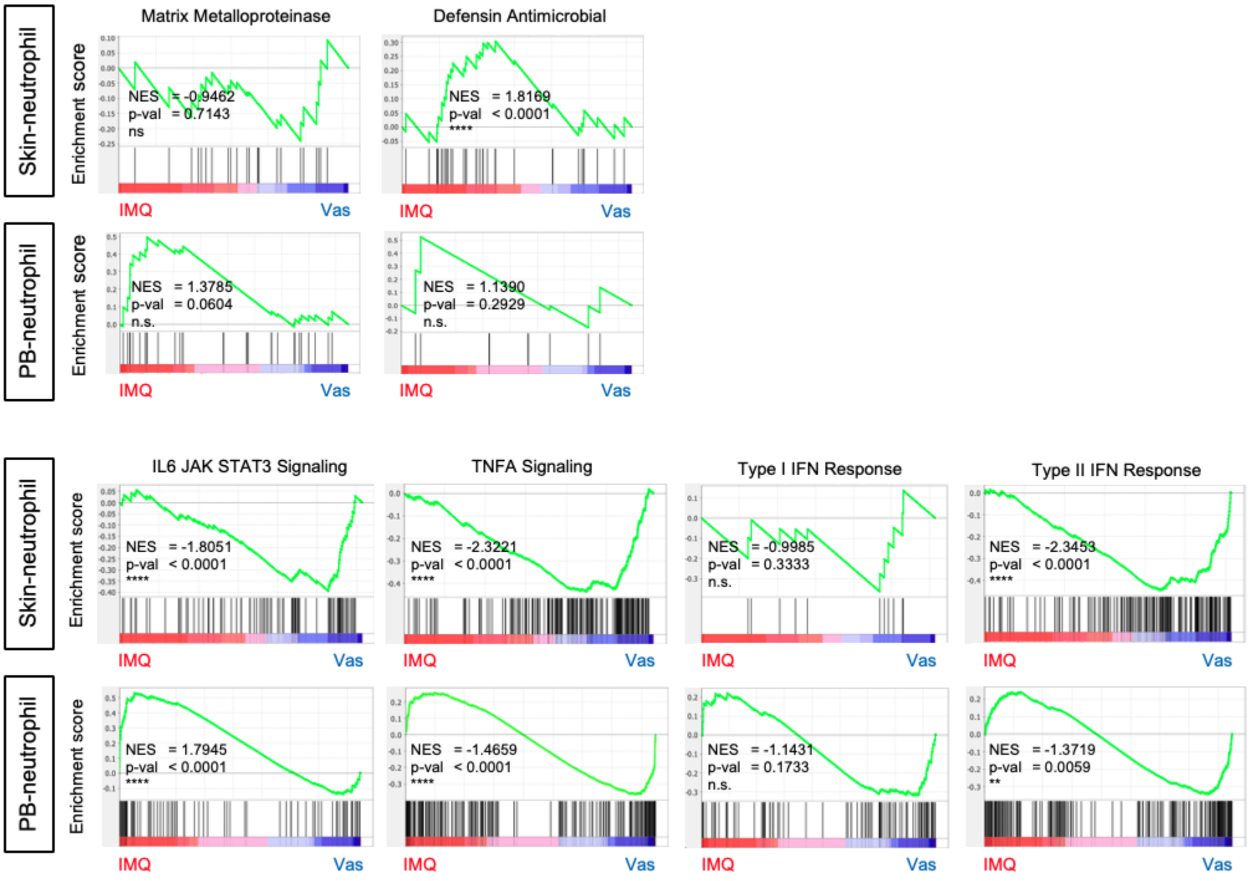

Explanation Figure 9. GSEA analysis of skin and peripheral blood treated with Vas (Vaseline) or IMQ (Imiquimod).

5. In addition, how the accumulated pathogenic neutrophils exacerbate the psoriatic changes remains obscure. Although the authors have attempted to correlate Il17a gene expression in infiltrated neutrophils with psoriatic skin changes, the data do not address to what degree it contributes to cutaneous IL-17A protein levels. The data that cutaneous neutrophil depletion leads to subtle decrease in skin IL-17A expression (Figure 2H) rather supports alternative possibilities. For instance, as indicated elsewhere, IL-17A cutaneous tone could be enhanced by neutrophil-mediated augmentation of Th17 or gamma/delta T cell function (Lambert et al. J Invest Dermatol. 2019, PMID: 30528823). Does neutrophil depletion or G-CSF neutralization alter cell numbers or function of cutaneous Th17 and gamma/delta T cells?

Author's response: We appreciate this thoughtful comment. Provided fluorophore-conjugated IL-17A is commercially available and used to detect IL-17A expression in CD4 T-cell (Th17), we attempted to apply the method described by another group (Enamorado et al. Cell 2023, PMID: 36640762). However, we failed to detect a clear IL-17A fluorescent signal above a threshold in

CD4+ helper T and TCRγδ+ T cells irrespective of stimulation with PMA/ionomycin for 150 min (Explanation Figure 10). We speculate that this failure of detection was due to the difference in tissue preparation: Enamorado et al., used ear skin that was digested for 90 min, while we used dorsal skin which requires the digestion for 180 min, which is twice longer than the cited protocol (for the details, see Explanation Table 1 below). The extended intensive digestion might have degraded the IL-17A protein and impaired cellular function to *de novo* production of IL-17A upon stimulation.

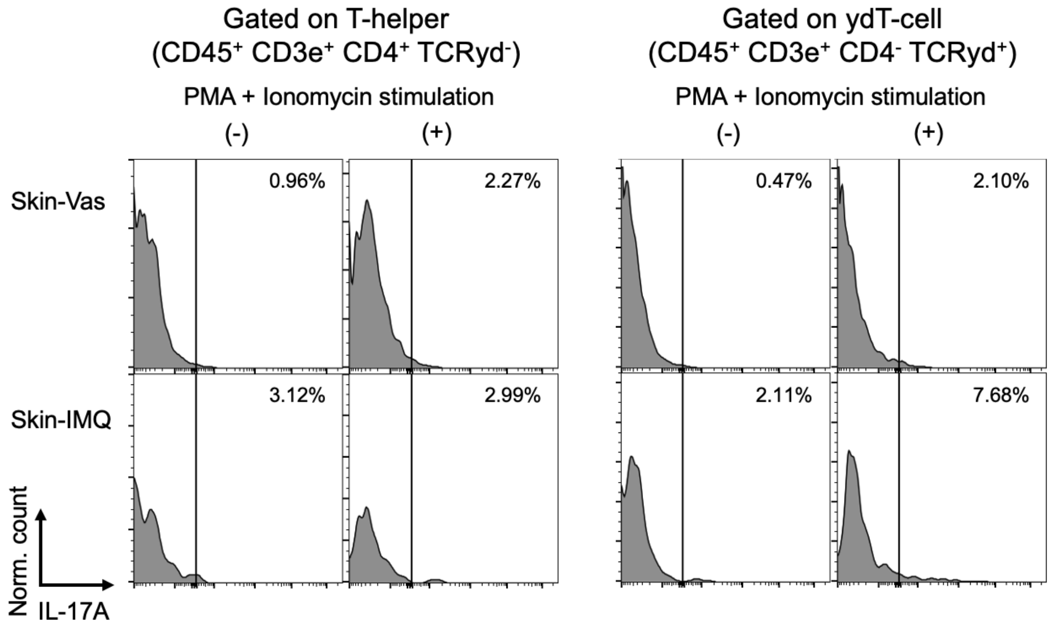

Explanation Figure 10. Flowcytometric IL-17A measurement from digested dorsal skin.

**Digestion condition**	Kosasih et al.	Enamorado et al.
Skin source	Dorsal skin	Ear skin
Digestion condition	37C, 5% CO_2, 180 min	37C, 5% CO_2, 90 min
Base media	RPMI 1640 with 10mM HEPES	RPMI 1640 with 20mM HEPES
Digestion enzymes	Liberase TM mix 62.5 µg/mL and DNAse 50µg/mL	Liberase TM mix 250 µg/mL and DNAse 100 µg/mL
Media supplements	-	2mM glutamine, 1mM sodium pyruvate, 1mM NEAA, 50µM B-mercaptoethanol, 100 U/mL penicillin, 100 mg/mL streptomycin

Explanation Table 1. Digestion and *in vitro* stimulation of the skin for IL-17A detection

Following unsuccessful flow cytometric analysis, we switched our methodology to qPCR to quantify the *Il17a* gene expression. Firstly, the *Il17a* level in the steady state (Vas) myeloid cells including neutrophils and CD11b$^+$ Ly6G$^-$ cells was detectable by qPCR and one log lower than that of γδ T or CD4+ helper T cells. Upon psoriasis induction (IMQ), neutrophils upregulate *Il17a*

expression by about 100-fold like γδ T cells as a positive control, whereas other myeloid cells defined by $CD11b^+$ $Ly6G^-$ cells and CD4+ helper T cells showed no change (Explanation Figure 11). We have added this figure to Figure 2D.

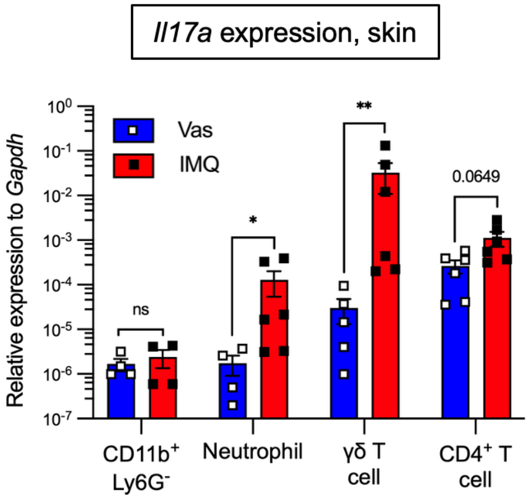

Explanation Figure 11. *Il17a* level in healthy and psoriatic skin in different cell types in skin.

Next, we tested whether neutrophils regulate number of CD4+ helper T and TCRγδ+ T cells and their IL-17A production. To this end, we depleted neutrophils with anti-Ly6G antibody injection in the psoriatic mice and compare the *Il17a* gene expression in TCRγδ+ T cells (γδ T cells) and CD4+ helper T cells cell. There was no significant alteration in the cell number of both fractions inside the psoriatic skin with or without neutrophil depletion. Functionally, neutrophil depletion resulted in about one-fifth log lower in *Il17a* expression in γδ T cells, with no detectable effect on CD4+ helper T cell fraction in psoriatic skin (Explanation Figure 12). These findings suggest that although neutrophil marginally affect abundance of γδ T cells or CD4+ helper T cells, they supports IL-17A production by γδ T cells. We have transferred this figure to Appendix Figure S2D and S2E

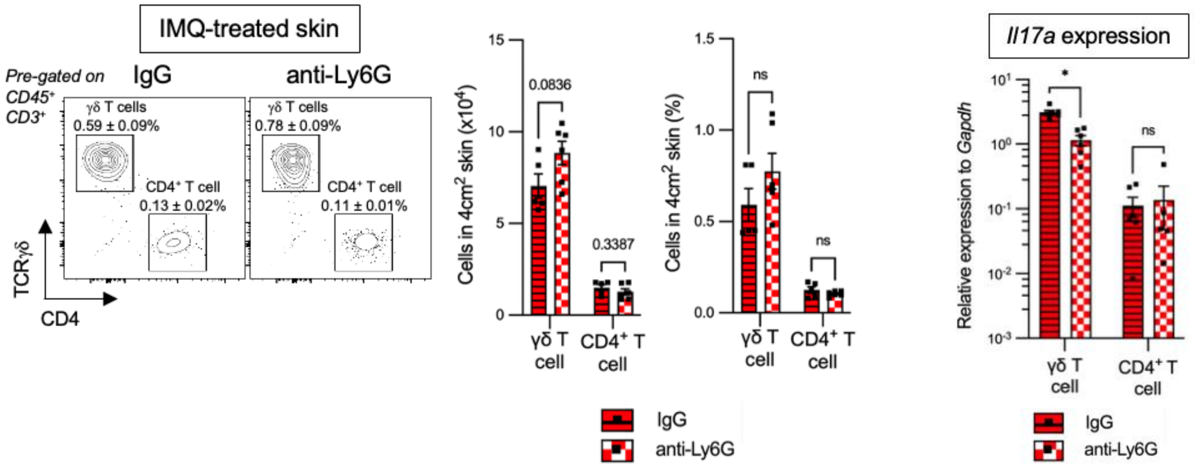

Explanation Figure 12. γδ T cells and CD4+ helper T cells' abundance (upper) and their *Il17a* expression (lower) in the psoriatic skin following neutrophil depletion.

Together, these data suggest that neutrophils represent a key regulator of cutaneous IL-17A upon psoriasis, either through direct secretion or by promoting γδ T cells' IL-17A production.

6. Finally, as the above conclusions rely solely on the IMQ-induced acute psoriasis model, it would be informative if they could be derived from another psoriasis model. IMQ is known to induce unintended systemic inflammation due to grooming-associated ingestion (Gangwar et al. J Invest Dermatol. 2022, PMID: 34953514), and "pathological crosstalk between skin and BM in psoriatic inflammation" could be strengthened by an intradermal injection model.

Author's response: Thank you for bringing this important point. Regarding the systemic inflammation upon psoriasis, the above-cited study reported increased IFN-B expression in the intestine of IMQ-ingested animal (Grine L et al. Sci Rep. 2016, PMID: 26818707 in Gangwar et al. J Invest Dermatol. 2022, PMID: 34953514). We examined systemic level of several pro-inflammatory cytokines including IFN-β, IFN-γ, and IL-6 and found no systemic increase in any of these cytokines tested, except for a slight IFN-γ downregulation (Explanation Figure 13), which suggests that grooming-associated ingestion most unlikely occurred.

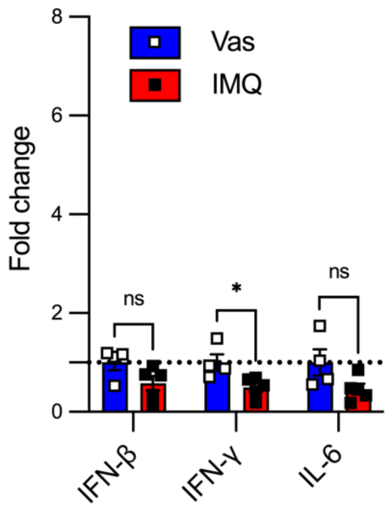

Explanation Figure 13. Level of inflammatory cytokines in the mouse serum

Having that shown, we also agreed with the reviewer's comment that intradermal system would strengthen the skin-bone marrow crosstalk. We did intradermal administration of anti-G-CSF antibody and demonstrated a skin-BM communication phenomenon (Explanation Figure 1 and Explanation Figure 21-26 below in the Reviewer#2's comment). Regarding the cytokine injection model for psoriasis, there are at least 9 different models with each accounting for a specific cytokine pathway that is useful to delineate targeted mechanisms despite the obvious weakness in an artificially exaggerated signaling (Gangwar et al. J. Invest. Dermatol 2022, PMID: 34953514). We believe that our study has provided a framework that would benefit future investigations to identify additional molecular pathways regulating skin-BM communication either using some/all of those cytokines or any other alternative models.

7. Figure 1E shows multiple elongated Ly6G+ structures in d0-2 control and d0 IMQ skins that do not appear to be neutrophils.

Author's response: Thank you for this. The elongated structures detected in the intravital microscopy are not of neutrophil's morphology, but autofluorescence from the skin bulge regions (Wun et al. J Invest Dermatol. 2005, PMID: 15816847). We eliminated these unspecific signals from any quantification (Figure 1F, S1G, and S1H). We have therefore added an additional explanation in Materials and Methods section page 15 line 531- line 532. "*Of note, the elongated structures detection resembling hair bulge were autofluorescent signals. They were removed from further analysis.*"

8. In Figure 2C, the bottom GSEA seems to be showing type II IFN response, not type I IFN, according to the text.

Author's response: Thank you for the comment, we have corrected this misspelling in Figure S2A.

9. For the BM analysis in Figures 3, 5, S3, and S5, it would be informative if BM cellularity and numbers of committed myeloid progenitors (e.g., GMPs) are shown.

Author's response: We examined the kinetics of the bone marrow cellularity and GMPs across 4 days of psoriasis induction. The bone marrow cell number was lower than Vas control along that span with reaching the bottom at 2 days. The GMP number followed a similar kinetics to that of the BM-cellularity. This has an analogy with the finding in previous report showing a rapid reduction of GMPs in the bone marrow within 2 days following systemic G-CSF administration (Hirai et al. Nat. Immunol. 2006, PMID: 16751774). From 2 days to 4 days, the GMP number rapidly increased to slightly above basal number (Explanation Figure 14). This timely coordinated expansion suggests a significant supply of GMPs from the differentiating upstream myeloid progenitors (Figure 3B).

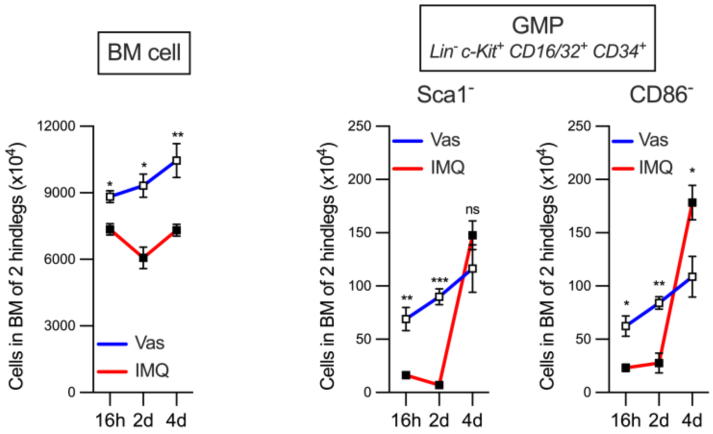

Explanation Figure 14. Number of bone marrow and GMP cells in the mice treated with topical Vas/IMQ.

When the psoriatic mice with elevated G-CSF level was injected with anti-G-CSF or IgG-isotype antibody, both bone marrow cellularity and GMP numbers showed no difference at 4 days (Explanation Figure 15). Similar GMP number is observed in IgG or anti-G-CSF antibody injected mice at 4 days IMQ. This is likely due to the snapshot analysis when the numerical recovery period has been achieved for the HSPCs fraction (Explanation Figure 14). Importantly, the notion that anti-G-CSF antibody injection to psoriatic mice reduced granulocytes in the bone marrow, peripheral blood, and skin suggest that G-CSF is a key mediator in psoriatic driven emergency granulopoiesis on top of unlikely case of ineffective anti-G-CSF antibody treatment.

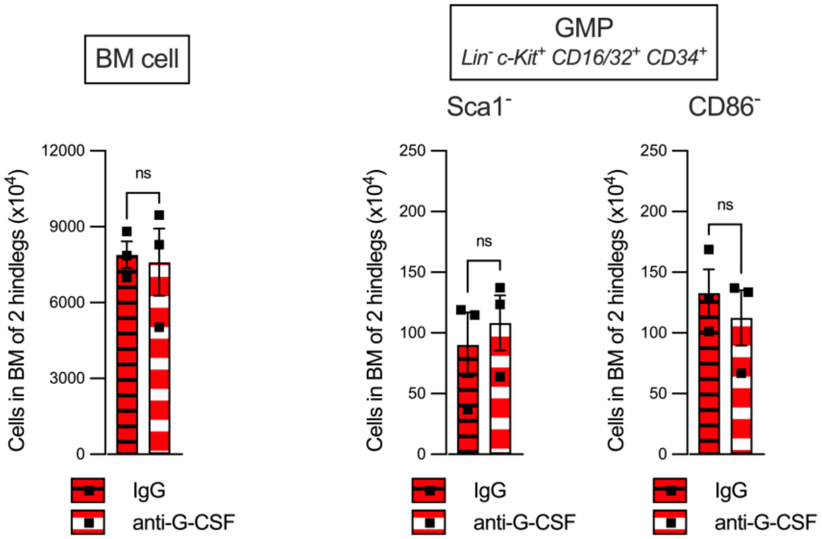

Explanation Figure 15. Bone marrow and GMP cell number in the IMQ-treated mice injected with anti-G-CSF or IgG-isotype antibody at 4 days post-treatment.

Taken together, these data suggest a G-CSF mediated emergency granulopoiesis occurrence in the IMQ-induced psoriasis.

10. In Figures 6B, in which cluster of human skin cells IL-17A expression would be enriched?

Author's response: Thank you for this important point. The IL-17A expression is found in the T-cell cluster (Explanation Figure 16). We also expected to see IL-17A contribution from other cell subset/s, in particular neutrophil. However, due to the fragile nature of tissue-infiltrating neutrophils (similar to our experience with skin-neutrophil RNA-seq in Figure 2, S2) and thereby, technical difficulty to get enough sequencing reads from them, this dataset (GSE173706) doesn't contain neutrophils, but monocytes, macrophages, and dendritic cells among the myeloid subset (Explanation Figure 17). With this, it leaves open the question on what potential contribution of IL-17A produced by neutrophils is in human psoriasis (Reich et al. Exp. Dermatol. 2015, PMID: 25828362).

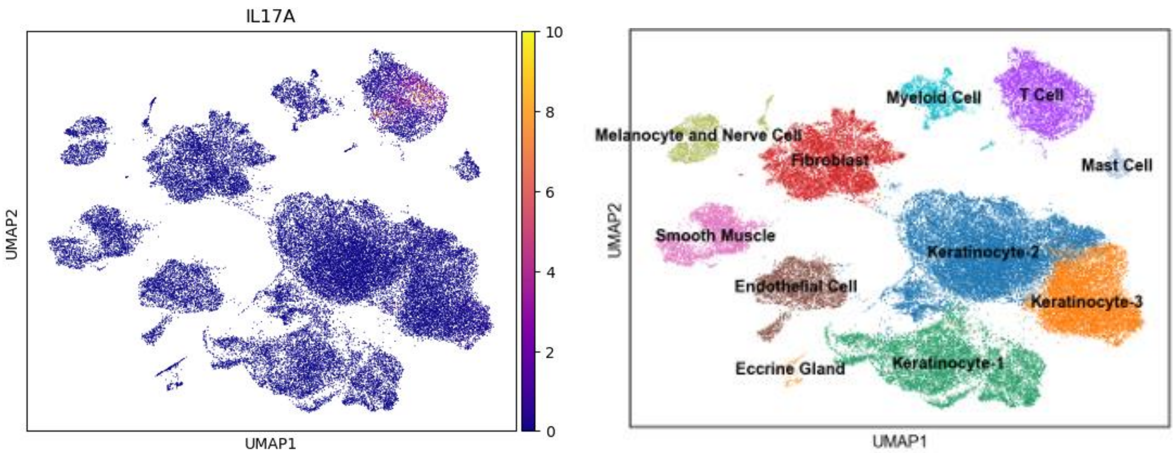

Explanation Figure 16. *IL17A* expressional map in human skin scRNAseq dataset (GSE173706)

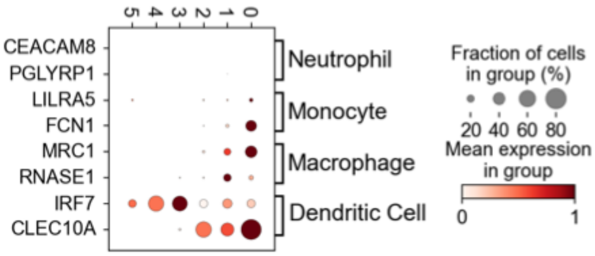

Explanation Figure 17. Cell annotation of myeloid subset (GSE173706)

Reviewer#2

1. Interpretation of neutrophil transcriptomic changes (Figure 2)

The RNA-seq analysis reveals substantial downregulation of several canonical pro inflammatory pathways in neutrophils from psoriatic skin, including IL-6, IL-1, and type II interferon signaling. The authors should discuss the functional relevance of this unexpected transcriptional repression. For example, does this indicate a shift toward specialized effector functions rather than classical cytokine responsiveness? More importantly, the most striking transcriptional change is the upregulation of NADPH oxidase-related genes (e.g., Nox1, Nox3, Nox4, Enox2). This suggests an oxidative stress-driven pathogenic mechanism, potentially more relevant than IL-17A production. Yet this aspect is not explored in the manuscript. Assessing ROS levels or oxidative neutrophil effector functions in this model would considerably strengthen the mechanistic link. Conversely, although IL-17A is upregulated in neutrophils, neutrophil depletion reduces total Il17a expression in skin only partially. This indicates that neutrophils are unlikely to be the dominant IL-17A source in the lesion. The authors' focus on neutrophil-derived IL 17A therefore seems overstated. A more rigorous assessment-e.g., conditional deletion of Il17a specifically in neutrophils-would be required to establish its true contribution. Taken together, the data suggest that oxidative programs, rather than IL-17A production, may represent the principal pathogenic axis downstream of neutrophils, and this deserves deeper discussion.

Author's response: We thank Reviewer#2 for this valuable input. We agree that our RNA-seq data suggest that neutrophils may exert broader functional effects beyond IL-17A alone. Neutrophils possess multiple effector mechanisms, including cytokine secretion, oxidative burst, protease release, activation of adaptive immunity, and NETosis (Burn GL et al. Immunity 2021, PMID: 34260886). Moreover, neutrophils are functionally heterogeneous, and their effector functions are highly dependent on environmental cues such as pathogen signals, immune checkpoint molecules, neuroimmune mediators, metabolic signals, etc. (Zhang F et al. STTT, PMID: 39638788).

In our RNA-seq dataset, psoriatic neutrophils exhibited downregulation of canonical pro-inflammatory pathways, such as IL-6, IL-1, and type II interferon signaling, while showing enrichment of NADPH oxidase related genes together with a modest *Il17a* expression. To further validate this observation, we performed ROS measurement together with RT-PCR validation of *Il17a*. We found that psoriatic neutrophils displayed significantly elevated ROS level as predicted from RNA-seq and the elevated ROS in the psoriatic neutrophils were observed in bone marrow, peripheral blood, and skin. These data suggest that neutrophils could acquire an enhanced oxidative capacity during their development, trafficking, as well as upon reaching the target tissue in psoriasis condition (Explanation Figure 18). We have split and added this figure to Figure 2C and Appendix Figure 2B.

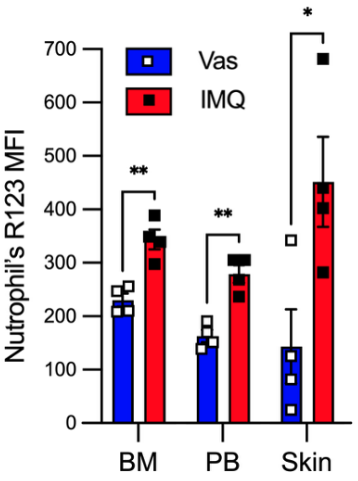

Explanation Figure 18. ROS measurement in the neutrophils from different pools. Vas: Vaseline, IMQ: Imiquimod.

We therefore agree that neutrophil-derived IL-17A does not represent the dominant pathogenic effector, but rather enhances the pathogenesis, as its psoriatic-induced upregulation was approximately one-tenth of that observed in $\gamma\delta$ T cells (Explanation Figure 9) and neutrophil depletion diminishes IL-17A production from $\gamma\delta$ T cells without affecting their cell number (Explanation Figure 12).

Based on these findings, we added a paragraph (Discussion, page 10-11, par. 4, line 331 to line 350), *"Neutrophils harbor several modes of action covering cytokine secretion, oxidative burst, protease release, adaptive immunity induction, and NETosis, which preferential initiation is*

highly dependent on microenvironmental cues (pathogen signals, neuroimmune mediators, metabolic signals, etc.).(Burn et al. Immunity 2021, PMID: 34260886 and Zhang et al. Signal Transduct Target Ther. 2024, PMID: 39638788) Our analysis show that neutropoiesis in psoriasis are primed for ROS production which would be the net results of chemoattractant, pro-inflammatory cytokines, or microbial signals sensing.(El-Benna et al. Immunol Rev. 2016, PMID: 27558335) High ROS production may induce DNA damage that compromises cellular function of surrounding cells and themselves, namely cytokine secretion (Harbort et al. Blood 2015, PMID: 26491069). This notion is in line with our observation that psoriatic neutrophils and $\gamma\delta$ T cells had a downregulation of canonical cytokine pathway and IL-17A, respectively, plausibly linked to their heightened superoxide level. An alternative possibility is that it may simply reflect an amplified ROS-loaded immature neutrophils generally observed in inflammatory granulopoiesis (Wang et al. JCI Insight 2020, PMID: 32960815). The egress of immature neutrophils position them in a transitional functional state, thereby allowing further reprogramming/adaptation in target tissue (Ng et al. Science 2024, PMID: 38207030). Our psoriatic skin indeed showed that skin-infiltrating neutrophils acquire IL-17A secretion property alongside IFNG downregulation. Given that psoriatic skin milieu is overloaded with pro-inflammatory cytokine, IL-23 (Griffiths et al. Lancet 2021, PMID: 33812489) and that IL-23 license neutrophils for IL-17A production (Li et al. Cell Mol Immunol. 2018, PMID: 28690333 and Furuya et al. J Autoimmun. 2024, PMID: 38301504), we think that IL-23 may contribute to the observed functional shift in psoriatic neutrophils. Of note, downregulated IFNG signature supports this interpretation, as IL-17A and IFNG are inversely correlated across multiple model systems (Lee et al. PLoS One 2013, PMID: 23613752, Ajendra et al. Mucosal Immunol. 2020, PMID: 32636457, and Marié et al. eLife 2021, PMID: 34378531)".

2. Human data reanalysis (Figure 6):
The re-analysis of bulk and single-cell RNA-seq datasets is valuable but incomplete. Several mechanistically relevant questions could be addressed with the available data:
2.1. GM-CSF (CSF2) is also strongly upregulated in psoriatic lesions (bulk RNA-seq). It would be informative to determine whether endothelial cells also express CSF2 in the scRNA-seq dataset, as this would suggest coordinated regulation of myeloid-supporting cytokines.
2.2. Myeloid cell subsets should be examined more closely. A comparison of human myeloid transcriptomes with the mouse neutrophil RNA-seq would clarify whether similar IL-17A-related or NADPH oxidase-related signatures occur in human disease. In particular, which cell types express IL17A in human lesions?
2.3. Chemokine production should be attributed to specific cell types. Bulk RNA-seq confirms strong induction of CXCL1, CXCL2, CXCL5, but the scRNA-seq dataset allows determining whether these chemokines originate from endothelial cells or other stromal/immune populations. This information is important for defining whether endothelial cells coordinate both neutrophil recruitment and granulopoiesis.
Addressing these points would make the human-mouse comparison substantially stronger.

Author's response: Thank you for pointing these important issues. By reanalyzing the dataset, we found several points regarding the comments, as follows:

2.1) *CSF2* is expressed by T-cell cluster in the human skin dataset (Explanation Figure 19), in agreement with previous murine study (Hartwig et al. Cell Reports. 2018, PMID: 30590032). We have added this data to the revised Figure S6F. We have added this figure to Appendix Figure S6F.

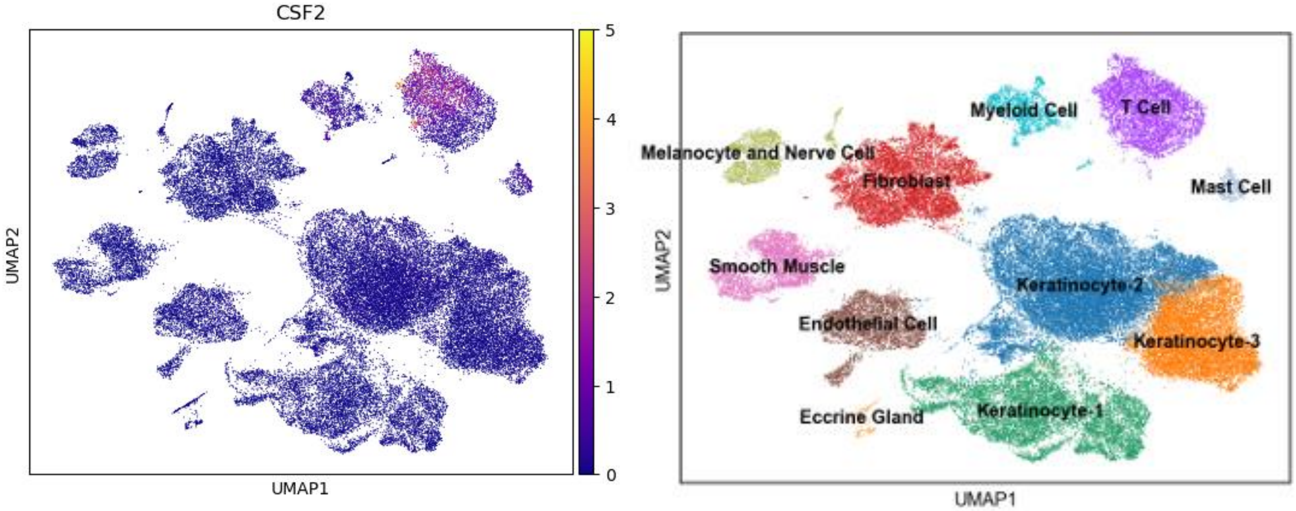

Explanation Figure 19. CSF2 expression in human skin scRNAseq dataset (GSE 173706)

2.2) The dataset clearly shows T-cell cluster as the main *IL17A* source (Explanation Figure 18 above) (also see the point #10 from Reviewer#1). The dataset, however, doesn't contain phenotypic neutrophils *(CEACAM (CD66b*) and *PGLYRP1*) but monocytes, macrophages, and dendritic cells (Explanation Figure 16 above). This loss was probably due to a technical limitation given the difficulty in capturing sequencing reads from fragile neutrophils. Therefore, it is no longer possible to reanalyze IL-17 expression in the absent neutrophils.

2.3) Reanalysis of CXCLs in the human scRNAseq dataset (GSE173706) clarified their secretion dynamics and cellular sources under normal and psoriatic condition. In normal skin, all examined cell subsets show only low *CXCLs* expression. In contrast, psoriatic skin exhibits significant *CXCLs* upregulation with distinct cell subsets, clearly showing dramatic upregulation that could potentially be the major *CXCLs* source. *CXCL1* is markedly upregulated in fibroblasts, myeloid cells, and melanocyte and nerve cells. *CXCL2* is strikingly upregulated to myeloid cells, while *CXCL5* is dramatically increased in fibroblasts, myeloid cells, and mast cells (Explanation Figure 20). Taken together, these results suggest that *CXCLs* upregulation in the psoriatic skin is coordinatively executed by both stromal and immune compartments. Of note, psoriatic endothelial cells show minimal changes in *CXCL*s expression and downregulation of *CXCL2*, indicating that they are unlikely to be the major contributor to CXCL-mediated neutrophil recruitment. We have transferred this figure to Appendix Figure S6G.

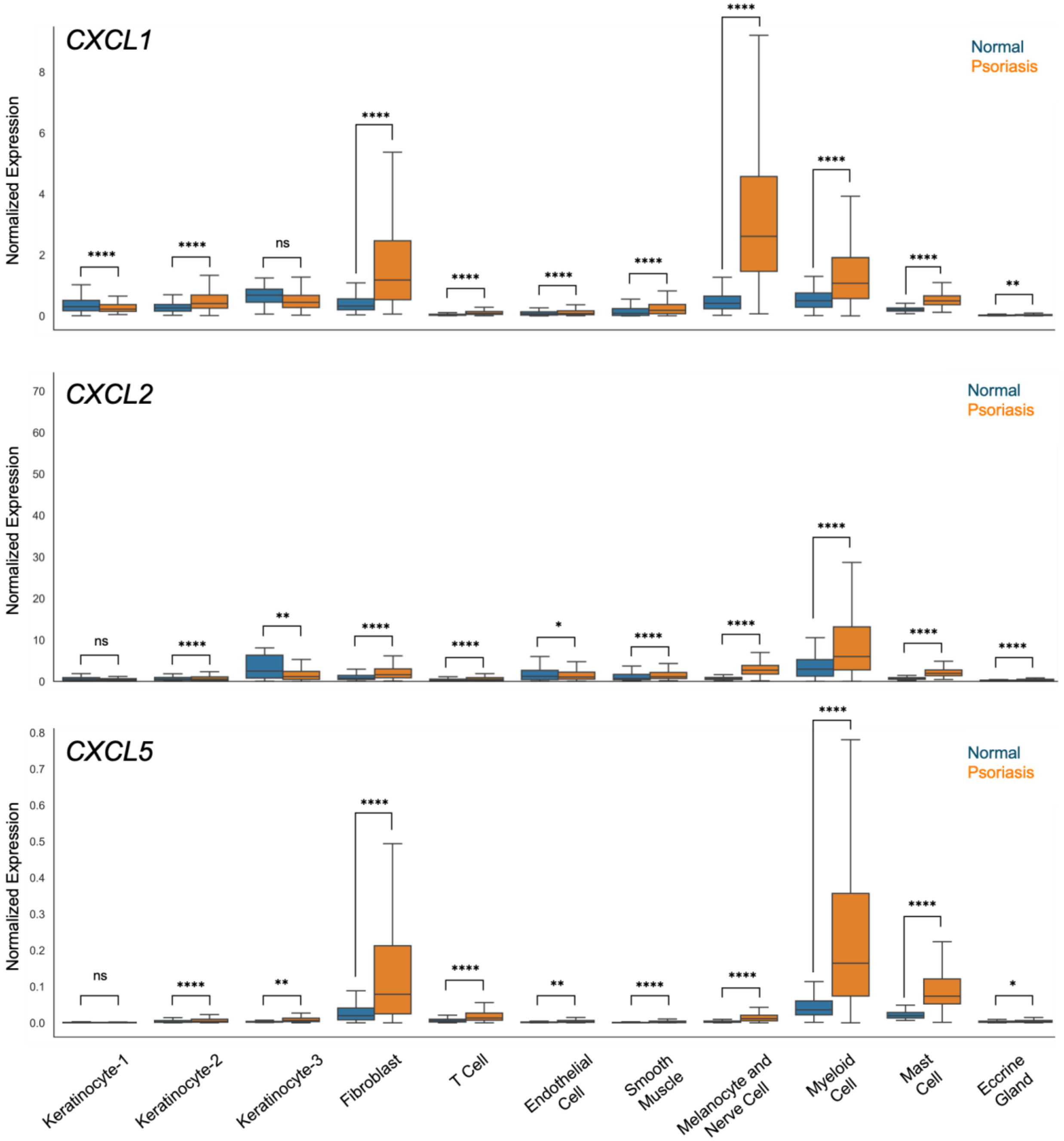

Explanation Figure 20. *CXCL1, CXCL2, CXCL5* expression in normal and psoriatic human skin from scRNAseq data (GSE173706).

Minor points

1. Line 319: the text likely refers to Figure S4, not S3.

Author's response: It has been corrected.

2. Line 338: "psoriatic" is misspelled.
Author's response: It has been corrected to *"psoriatic"* now in page 7, line 200.

Referees cross-commenting
I agree with Reviewer 1 that the contribution of EC-derived G-CSF to circulating G-CSF levels and to emergency myelopoiesis requires additional genetic or neutralization experiments to be fully established.

Author's response: In addition to examining the serum G-CSF, we also assessed the emergency granulopoiesis-driving effect along with the other relevant biological readouts in the intradermal anti-G-CSF experiment:
Intradermal anti-G-CSF antibody injection in the psoriatic mice didn't significantly alter the LSK number in the bone marrow (Explanation Figure 21). It, however, significantly reduced the circulating neutrophils (Explanation Figure 22), as well as the erythema and scaling score particularly at the later disease period (Explanation Figure 23). We will add these figures to Appendix Figure S5B, S5C, and S5D.

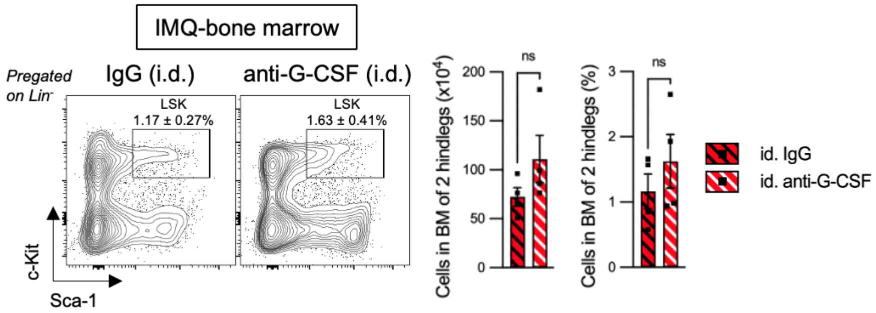

Explanation Figure 21. LSK fraction in the bone marrow. IMQ: Imiquimod, i.d.: intradermal.

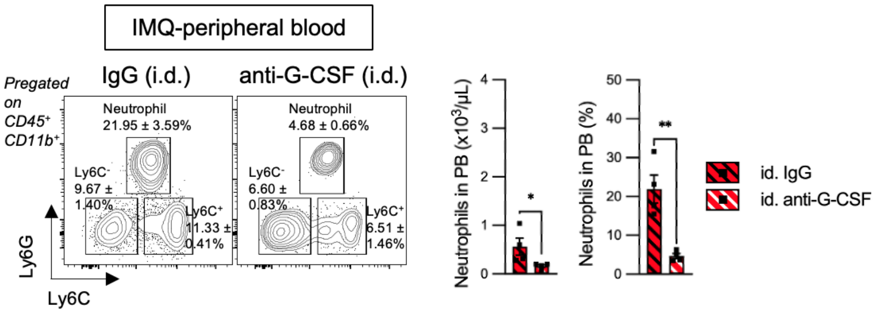

Explanation Figure 22. Neutrophil compartment in the peripheral blood. IMQ: Imiquimod, i.d.: intradermal.

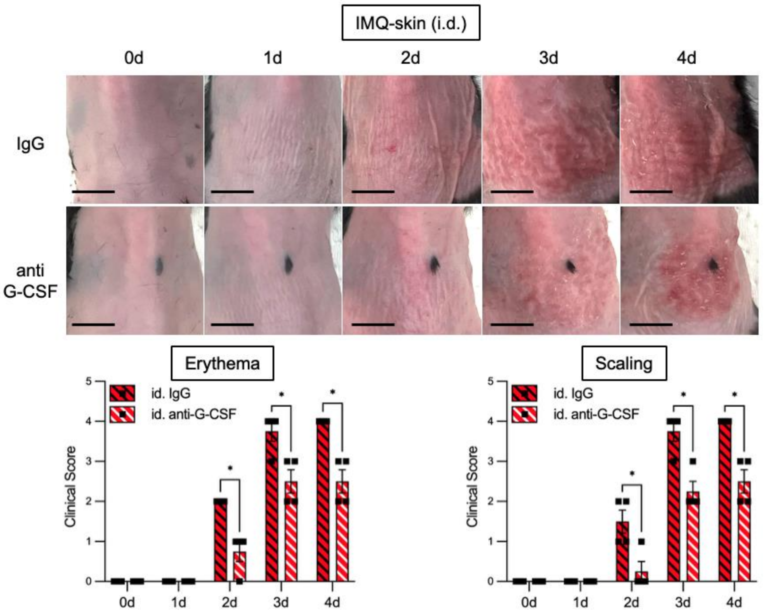

Explanation Figure 23. Clinical symptoms of the dorsal skin. IMQ: Imiquimod, i.d.: intradermal.

We observed a significant reduction in the cardinal emergency granulopoiesis signs, in which the immature granulocyte was lowered by about 30% without mature granulocytes alteration in the psoriatic BM treated with anti-G-CSF antibody intradermally (Explanation Figure 24). Also, the intradermal anti-G-CSF antibody treatment reduced ~75% of skin neutrophils (Explanation Figure 25), and ultimately lessened the psoriatic epidermal thickness by ~40% (Explanation Figure 26). We have added these figures to Main Figure 5C, 5D, and 5E.

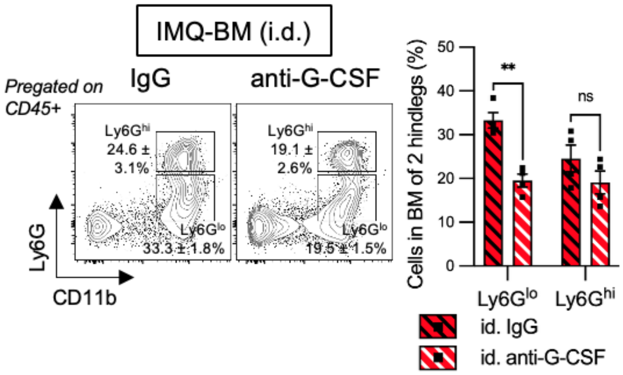

Explanation Figure 24. Immature and mature granulocytes in bone marrow. IMQ: Imiquimod, i.d.: intradermal.

Review COMMONS

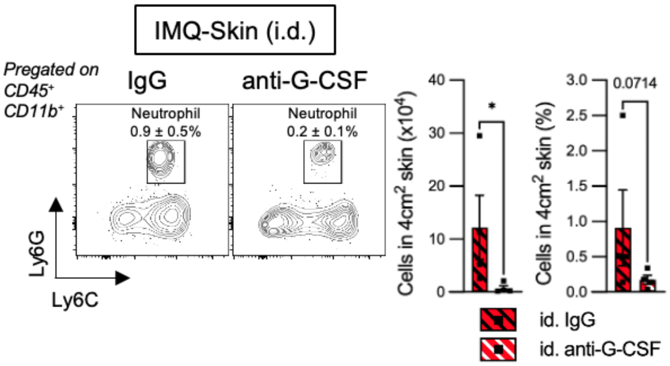

Explanation Figure 25. Neutrophils in the skin. IMQ: Imiquimod, i.d.: intradermal.

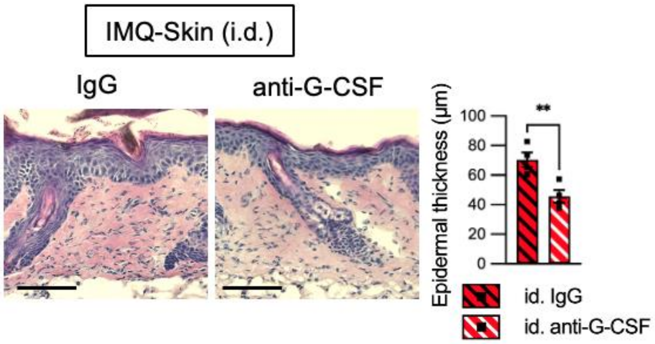

Explanation Figure 26. Cutaneous histology from the dorsal lesion. IMQ: Imiquimod, i.d.: intradermal.

Together, these data suggest that the emergency granulopoiesis and its entailing pathogenicity are driven by the skin-derived G-CSF as its cutaneous selective blocking recapitulated the systemic G-CSF inhibition.

Reviewer#3

- Place the work in the context of the existing literature (provide references, where appropriate). Psoriasis is extensively studied, a good recent reference- https://doi.org/10.1016/j.mam.2024.101306

Author's response: Thank you for Reviewer#3's suggestion. The suggested reference is important for an additional insight of myeloid cells contribution to psoriasis, both in isolated and across multicompartmental context. Therefore, we add a discussion point for this notion in the discussion, page 12, par. 7, line 391- line 394: "*Additionally, it would be informative to investigate other myeloid cells' contribution to psoriatic inflammation, especially eosinophils or mast cells that show their promising roles as potent inflammatory orchestrator in concert with multiple cell types encompassing neutrophils and beyond (Park et al. Mol. As. Med. 2024, PMID: 39191143).*"

- Do you have suggestions that would help the authors improve the presentation of their data and conclusions?
It is all good. May add graphical-abstract.

Author's response: Thank you for the reviewer's input, we agree that a graphical abstract will help the readers more clearly grasp the key messages of our manuscript. We have included this figure (Explanation Figure 27) in the revised manuscript.

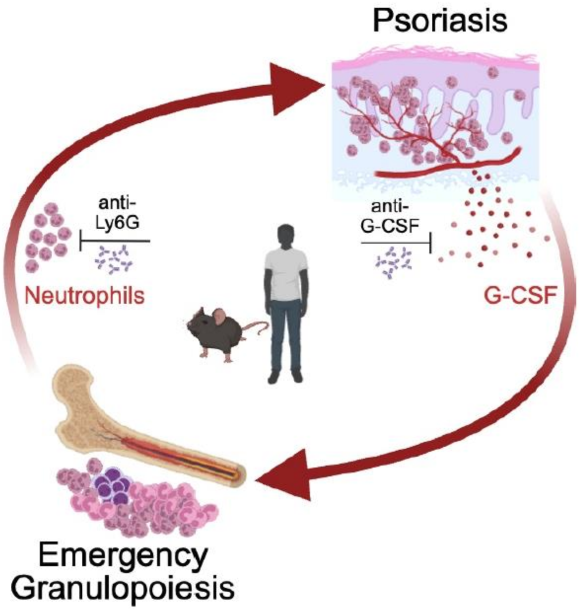

Explanation Figure 27. Graphical Abstract.

Major comments:
- Should the authors qualify some of their claims as preliminary or speculative, or remove them altogether?
No. It is very solid.

Author's response: Thank you for this comment.

- Would additional experiments be essential to support the claims of the paper? Request additional experiments only where necessary for the paper as it is, and do not ask authors to open new lines of experimentation.
Such a discovery clearly opens many options, and it is fascinating to suggest additional experiments for future studies. It is a complete study, best to publish as-is and let many to read and proceed with this new concept.

Author's response: We thank the reviewer for noting that the current experimental evidence is complete that no additional experiments are necessary at this stage. We agree that the discovery opens prospective directions for future studies.

- Are the suggested experiments realistic in terms of time and resources? It would help if you could add an estimated cost and time investment for substantial experiments.
N/A - I suggest no additional experiments at this point. Get it published and see how many will follow this new direction!

Author's response: We thank the reviewer for recognizing that the experimental data has been sufficient to be a foundation for the future research.

- Are the data and the methods presented in such a way that they can be reproduced?
Yes.

Author's response: Thank you.

- Are the experiments adequately replicated, and is the statistical analysis adequate?
Yes. The data are of very high quality.

Author's response: We are grateful that the reviewer view our replication strategy and statistical analysis are of a high quality.

Minor comments:
- Specific experimental issues that are easily addressable.
None. It is good as-is. One may always suggest minor things- but this one is better published so many laboratories may rush for this new direction. I think it will be interesting studying some long-term impacts, and changes not only of neutrophils but also of other innate cells, such as DCs, Macrophages, and Eosinophils - so it is best to let laboratories that focus on these cells know of the discovery and pursue independent studies.

Author's response: We appreciate the reviewer's assessment that our paper is already well set for the community to explore the newly proposed direction.

- Are the text and figures clear and accurate?
Yes.

Author's response: We thank the reviewer's evaluation. We have ensured that the text and figures in our manuscript are clear and accurate. Once again, we thank the reviewer for the encouraging and constructive appraisal. We are pleased that the reviewer found the manuscript already been strong and suitable for publication.

4th May 2026

Dear Prof. Takizawa,

Thank you for submitting your revised study. We have now received feedback from referees #1 and #2. As you will see below, they are overall satisfied with the revisions, and we will therefore be able to accept your manuscript once the following editorial matters are addressed:

1/ Referees' concerns:
Please address the remaining concerns by adequate discussion in the text.

2/ Manuscript text:
- Please remove the blue font text and only keep in track changes mode any new modification.
- Please update the order and headings of the sections in the manuscript text to: Abstract / The Paper Explained / Introduction / Results / Discussion / Methods / Data Availability / Acknowledgements / Disclosure and Competing Interests Statement / References / Figure Legends /
- Remove "Data not shown" (l.206): as per our guidelines, all new results discussed in the manuscript should be presented in the main or supplementary figures.
- Rename "Materials and Methods" to "Methods":
o Antibodies: please provide dilutions / concentrations.
o Statistics: please include a statement statement about sample size, and exclusion/inclusion criteria.
- BioRender: Please remove the sentence about the cartoon figures from the Acknowledgements and add a dedicated paragraph to the Methods using the following format:
Graphics:
(some of the... OR Figure #... OR synopsis) Graphics were created with BioRender.com.
- Data Availability: please provide specifif URLs for the deposited datasets.
- Please remove Author contributions from the manuscript text, and provide instead CRediT (Contributor Role Taxonomy) terms in the submission system. These replace a narrative author contribution section in the manuscript.
- Please rename "Ethics Declarations" to "Disclosure and competing interests statement".
- Our journal encourages inclusion of *data citations in the reference list* to directly cite datasets that were re-used and obtained from public databases. Data citations in the article text are distinct from normal bibliographical citations and should directly link to the database records from which the data can be accessed. In the main text, data citations are formatted as follows: "Data ref: Smith et al, 2001" or "Data ref: NCBI Sequence Read Archive PRJNA342805, 2017". In the Reference list, data citations must be labeled with "[DATASET]". A data reference must provide the database name, accession number/identifiers and a resolvable link to the landing page from which the data can be accessed at the end of the reference.

3/ Figures:
- Main and EV figures should be uploaded individually. Please note that we replaced Supplementary Information with Expanded View (EV) Figures and Tables that are collapsible/expandable online. EV Figures should be cited as 'Figure EV1, Figure EV2" etc... in the text and their respective legends should be included in the main text after the legends of regular figures. For the figures that you do NOT wish to display as Expanded View figures, they should be bundled together with their legends in a single PDF file called *Appendix*, which should start with a short Table of Content. Appendix figures should be referred to in the main text as: "Appendix Figure S1, Appendix Figure S2" etc. In the Appendix, blue font should be removed and if possible, figure legends should be moved under each corresponding figure.
- Please address the queries from our data editors in the figure legends:
1. Please note that the exact p values are not provided in the legends of figures 1B, C, F; 2B, C, D, F, G; 3B-E; 4B, C, E, F, G; 5B-E; 6A, C; S1 A, B, D, H; S2 A-D, F; S3C, S4 B, S5 A-C, D; S6B, E, G
2. Please indicate the statistical test used for data analysis in the legends of figures 1B, C, F; 2B, C, D, F, G, H; 3B-E; 4B, C, E, F, G; 5B-E; 6A, C; S1 A, B, D, E, H; S2 A-F; S3 C, E; S4 B, D, E, F; S5 A-C, D; S6B, E, G
3. Please note that the box plots need to be defined in terms of minima, maxima, centre, bounds of box and whiskers, and percentile in the legends of figures 6C, S6 G
4. Please note that information related to n is missing in the legends of figures 6C, S6 G
5. Please note that the scale bar needs to be defined for figures S1 A, S2 F, S5 D

4/ Thank you for providing Source Data. Please also provide FACS raw data.

5/ Please remove the visual abstract and synopsis text from the manuscript file and upload them as individual files. I introduced minor edits in your text, please let me know if you agree:

A G-CSF-mediated skin-bone marrow axis that contributes to psoriasis pathogenesis was characterized both in mouse and human.

• Excess neutrophil number in psoriatic skin exacerbates inflammation.
• Skin endothelial cells are a key source of G-CSF in mouse and human psoriasis.
• Skin-derived G-CSF drives pathogenic granulopoiesis in psoriasis.
• G-CSF inhibition attenuates psoriasis symptoms.

The visual abstract to illustrate should be uploaded as jpeg/tiff/PNG file 550 px wide x 300 px high.

6/ As part of the EMBO Publications transparent editorial process initiative (see our Editorial at http://embomolmed.embopress.org/content/2/9/329), EMBO Molecular Medicine will publish online a Review Process File (RPF) to accompany accepted manuscripts.
This file will be published in conjunction with your paper and will include the anonymous referee reports, your point-by-point response and all pertinent correspondence relating to the manuscript. Let us know whether you agree with the publication of the RPF and as here, if you want to remove or not any figures from it prior to publication.
Please note that the Authors checklist will be published at the end of the RPF.

I look forward to receiving your revised manuscript.

Yours sincerely,

Lise Roth

Lise Roth, PhD
Senior Editor
EMBO Molecular Medicine

***** Reviewer's comments *****

Referee #1 (Remarks for Author):

The authors have performed additional experiments, and the newly provided data address most of my previous concerns regarding the cellular source of circulating G-CSF and its role in the infiltration and accumulation of pathogenic neutrophils in the skin. However, several important issues remain and should be addressed in greater depth to ensure an accurate interpretation of the data.

1. Source of circulating G-CSF
Figure S5A suggests that affected skin is largely responsible for the elevated circulating G-CSF levels in the psoriasis model. However, the current presentation does not conclusively demonstrate that skin endothelial cells (ECs) are the predominant source of G-CSF. The trend toward increased G-CSF expression across multiple non EC populations (Figures 4F-4G) suggests that additional skin compartments may contribute meaningfully. A more accurate interpretation would therefore be that psoriatic skin is a major source of elevated G-CSF, with skin ECs representing one of several contributing cell types rather than the exclusive or dominant source.

2. Mobilization versus emergency granulopoiesis
Although the authors now include sufficient biological replicates for bone marrow progenitor analyses and additional supporting data, the effect of skin-derived G-CSF on mobilization remains substantial and should not be considered negligible. Circulating G-CSF levels remain elevated from 16 hours through day 4, indicating sustained mobilization, in contrast to the single-injection G-CSF models referenced by the authors (PMIDs: 7521686, 12084168).

The kinetic changes in bone marrow cellularity and absolute GMP numbers shown in the Explanation Figure 14 are critical for interpreting the remaining bone marrow results and should be incorporated into the main figures. The sustained reduction in bone marrow cellularity through day 4 strongly suggests that the apparent increase in Ly6G-lo granulocyte frequencies (Figure 3E) primarily reflects mobilization of Ly6G-hi mature granulocytes into the peripheral blood rather than increased production of immature granulocytes from upstream progenitors. The marked depletion of GMPs by day 2 further suggests either direct progenitor mobilization or rapid differentiation followed by mobilization. Consistent with this interpretation, transplanted HSPCs do not exhibit differentiation skewing (Figure S3E), arguing against G-CSF-driven myeloid bias at the HSPC level. The increased cycling observed in HSPCs (Figure 3C) may instead reflect a compensatory response to granulocyte depletion, as previously described (PMID: 31886826). These alternative interpretations should be discussed explicitly and acknowledged as

limitations of the study.

3. Role of CXCR2 in neutrophil infiltration
Based on the Explanation Figure 3, G-CSF may enhance neutrophil infiltration through upregulation of CXCR2. The authors should cite the study by Sumida et al. (PMID: 24663678) and explicitly discuss the role of skin-derived G-CSF during the neutrophil infiltration phase, as this mechanism is directly relevant to the manuscript's central conclusions.

Referee #2 (Comments on Novelty/Model System for Author):

The imiquimod-induced mouse model is appropriate for this study despite its known limitations. Psoriasis is a complex systemic inflammatory disease involving multiple interacting compartments, including skin-resident cells, immune cells, vasculature, circulating mediators, and bone marrow hematopoiesis. At present, there are no non-animal alternatives that can faithfully reproduce this level of cellular and organ-level complexity.

In this particular study, the model is especially justified because the central finding concerns a skin-bone marrow axis, in which inflamed skin induces systemic G-CSF production, emergency granulopoiesis, and subsequent neutrophil accumulation in the skin. This type of cross-organ communication cannot be adequately modeled in vitro.

The imiquimod model also has specific limitations, including its acute nature and the fact that imiquimod-driven inflammation does not fully reproduce chronic human psoriasis. These limitations should be acknowledged. Where feasible, the conclusions could be further strengthened by validating key findings in complementary psoriasis models or human datasets. However, for the mechanistic question addressed here, the use of mice is justified and appropriate.

From an ethical perspective, the model induces skin inflammation and discomfort, so experiments should use the minimum number of animals required for statistical robustness, include appropriate monitoring and humane endpoints, and apply refinement measures to minimize distress.

Referee #2 (Remarks for Author):

I have carefully evaluated the revised version of the manuscript and the authors' detailed point-by-point responses . Overall, I am satisfied with the revisions and the substantial effort made to address the concerns raised in the initial review.

The authors have significantly strengthened the manuscript, particularly by:

* providing additional experimental evidence supporting the role of G-CSF in driving emergency granulopoiesis,
* addressing the functional interpretation of neutrophil transcriptomic changes, including the relevance of NADPH oxidase pathways and ROS production,
* clarifying the contribution of neutrophils beyond IL-17A, and appropriately moderating previous claims,
* improving the human data analysis, including the identification of CSF2-expressing cell types and the cellular sources of chemokines.

Regarding my previous point on the human data (point 2.2), the authors clearly explain that neutrophils are not represented in the available scRNA-seq dataset due to technical limitations. While this is unfortunate and leaves an open question regarding the direct contribution of neutrophils in human psoriasis, I find the explanation reasonable and the limitation appropriately acknowledged.

Importantly, none of the remaining limitations undermine the main conclusions of the study, which are well supported by the experimental data.

In summary, I believe the manuscript is now suitable for publication and recommend acceptance.

***
Rev_Com_number: RC-2025-03286
New_manu_number: EMM-2025-23207-V2
Corr_author: Takizawa
Title: Skin-derived G-CSF activates pathological granulopoiesis upon psoriasis

The authors addressed the remaining editorial issues.

20th May 2026

Dear Prof. Takizawa,

Thank you for submitting your revised files. We are pleased to inform you that your manuscript is accepted for publication and is now being sent to our publisher to be included in the next available issue of EMBO Molecular Medicine.

Your manuscript will be processed for publication by EMBO Press. It will be copy edited and you will receive page proofs prior to publication. Please note that you will be contacted by Springer Nature Author Services to complete licensing and payment information.

You may qualify for financial assistance for your publication charges - either via a Springer Nature fully open access agreement or an EMBO initiative. Check your eligibility: https://link.springer.com/journal/44321/how-to-publish-with-us

Should you be planning a Press Release on your article, please notify the production team via this form in order to coordinate publication and release dates: https://forms.cloud.microsoft/e/eH4A8JVP6g

If you have any questions, please do not hesitate to contact the Editorial Office. Thank you for your contribution to EMBO Molecular Medicine.

Yours sincerely,

Lise Roth, Ph.D
Senior Editor
EMBO Molecular Medicine

>>> Please note that it is EMBO Molecular Medicine policy for the transcript of the editorial process (containing referee reports and your response letter) to be published as an online supplement to each paper. If you do NOT want this, you will need to inform the Editorial Office via email immediately. More information is available here: https://link.springer.com/partners/embo-press/editorial-policies#Peer%20review

***
Rev_Com_number: RC-2025-03286
New_manu_number: EMM-2025-23207-V3
Corr_author: Takizawa
Title: Skin-derived G-CSF activates pathological granulopoiesis upon psoriasis

EMBO Press Author Checklist

Corresponding Author Name: Hitoshi Takizawa	
Journal Submitted to: EMBO Molecular Medicine	
Manuscript Number: RC-2025-03286 (EMM-2025-23207-T)	

USEFUL LINKS FOR COMPLETING THIS FORM
The EMBO Journal - Author Guidelines
EMBO Reports - Author Guidelines
Molecular Systems Biology - Author Guidelines
EMBO Molecular Medicine - Author Guidelines

Reporting Checklist for Life Science Articles (updated January
This checklist is adapted from Materials Design Analysis Reporting (MDAR) Checklist for Authors. MDAR establishes a minimum set of requirements in transparent reporting in the life sciences (see Statement of Task: 10.31222/osf.io/9sm4x). Please follow the journal's guidelines in preparing your
Please note that a copy of this checklist will be published alongside your article.

Abridged guidelines for figures
1. Data
The data shown in figures should satisfy the following conditions:
- the data were obtained and processed according to the field's best practice and are presented to reflect the results of the experiments in an accurate and unbiased manner.
- ideally, figure panels should include only measurements that are directly comparable to each other and obtained with the same assay.
- plots include clearly labeled error bars for independent experiments and sample sizes. Unless justified, error bars should not be shown for technical
- if n<5, the individual data points from each experiment should be plotted. Any statistical test employed should be justified.
- Source Data should be included to report the data underlying figures according to the guidelines set out in the authorship guidelines on Data

2. Captions
Each figure caption should contain the following information, for each panel where they are relevant:
- a specification of the experimental system investigated (eg cell line, species name).
- the assay(s) and method(s) used to carry out the reported observations and measurements.
- an explicit mention of the biological and chemical entity(ies) that are being measured.
- an explicit mention of the biological and chemical entity(ies) that are altered/varied/perturbed in a controlled manner.
- the exact sample size (n) for each experimental group/condition, given as a number, not a range;
- a description of the sample collection allowing the reader to understand whether the samples represent technical or biological replicates (including how many animals, litters, cultures, etc.).
- a statement of how many times the experiment shown was independently replicated in the laboratory.
- definitions of statistical methods and measures:
  - common tests, such as t-test (please specify whether paired vs. unpaired), simple χ2 tests, Wilcoxon and Mann-Whitney tests, can be unambiguously identified by name only, but more complex techniques should be described in the methods section;
  - are tests one-sided or two-sided?
  - are there adjustments for multiple comparisons?
  - exact statistical test results, e.g., P values = x but not P values < x;
  - definition of 'center values' as median or average;
  - definition of error bars as s.d. or s.e.m.

Please complete ALL of the questions below.
Select "Not Applicable" only when the requested information is not relevant for your study.

Materials

Newly Created Materials	Information included in the manuscript?	In which section is the information available? (Reagents and Tools Table, Materials and Methods, Figures, Data Availability Section)
New materials and reagents need to be available; do any restrictions apply?	Not Applicable	

Antibodies	Information included in the manuscript?	In which section is the information available? (Reagents and Tools Table, Materials and Methods, Figures, Data Availability Section)
For **antibodies** provide the following information:<br>- Commercial antibodies: RRID (if possible) or supplier name, catalogue number and or/clone number<br>- Non-commercial: RRID or citation	Yes	Reagents and Tools Table

DNA and RNA sequences	Information included in the manuscript?	In which section is the information available? (Reagents and Tools Table, Materials and Methods, Figures, Data Availability Section)
**Short novel DNA or RNA including primers, probes**: provide the sequences.	Yes	Table EV1

Cell materials	Information included in the manuscript?	In which section is the information available? (Reagents and Tools Table, Materials and Methods, Figures, Data Availability Section)
**Cell lines:** Provide species information, strain. Provide accession number in repository **OR** supplier name, catalog number, clone number, and/**OR** RRID.	Not Applicable	
**Primary cultures:** Provide species, strain, sex of origin, genetic modification status.	Yes	Materials and Methods
Report if the cell lines were recently **authenticated** (e.g., by STR profiling) and tested for mycoplasma contamination.	Not Applicable	

Experimental animals	Information included in the manuscript?	In which section is the information available? (Reagents and Tools Table, Materials and Methods, Figures, Data Availability Section)
**Laboratory animals or Model organisms:** Provide species, strain, sex, age, genetic modification status. Provide accession number in repository **OR** supplier name, catalog number, clone number, **OR** RRID.	Yes	Materials and Methods
**Animal observed in or captured from the field:** Provide species, sex, and age where possible.	Not Applicable	
Please detail **housing and husbandry conditions**.	Yes	Materials and Methods

Plants and microbes	Information included in the manuscript?	In which section is the information available? (Reagents and Tools Table, Materials and Methods, Figures, Data Availability Section)
**Plants:** provide species and strain, ecotype and cultivar where relevant, unique accession number if available, and source (including location for collected wild specimens).	Not Applicable	
**Microbes:** provide species and strain, unique accession number if available, and source.	Not Applicable	

Human research participants	Information included in the manuscript?	In which section is the information available? (Reagents and Tools Table, Materials and Methods, Figures, Data Availability Section)
If collected and within the bounds of privacy constraints report on age, sex and gender or ethnicity for all study participants.	Not Applicable	

Core facilities	Information included in the manuscript?	In which section is the information available? (Reagents and Tools Table, Materials and Methods, Figures, Data Availability Section)
If your work benefited from core facilities, was their service mentioned in the acknowledgments section?	Yes	Acknowledgements

Design

Study protocol	Information included in the manuscript?	In which section is the information available? (Reagents and Tools Table, Materials and Methods, Figures, Data Availability Section)
If study protocol has been **pre-registered, provide DOI in the manuscript**. For clinical trials, provide the trial registration number **OR** cite DOI.	Not Applicable	
Report the **clinical trial registration number** (at ClinicalTrials.gov or equivalent), where applicable.	Not Applicable	

Laboratory protocol	Information included in the manuscript?	In which section is the information available? (Reagents and Tools Table, Materials and Methods, Figures, Data Availability Section)
Provide DOI OR other citation details if **external detailed step-by-step protocols** are available.	Not Applicable	

Experimental study design and statistics	Information included in the manuscript?	In which section is the information available? (Reagents and Tools Table, Materials and Methods, Figures, Data Availability Section)
Include a statement about **sample size** estimate even if no statistical methods were used.	Yes	Figure Legend
Were any steps taken to minimize the effects of subjective bias when allocating animals/samples to treatment (e.g. **randomization procedure**)? If yes, have they been described?	Yes	Materials and Methods
Include a statement about **blinding** even if no blinding was done.	Yes	Materials and Methods
Describe **inclusion/exclusion criteria** if samples or animals were excluded from the analysis. Were the criteria pre-established?<br><br>If sample or data points were omitted from analysis, report if this was due to attrition or intentional exclusion and provide justification.	Yes	Materials and Methods
For every figure, are **statistical tests** justified as appropriate? Do the data meet the assumptions of the tests (e.g., normal distribution)? Describe any methods used to assess it. Is there an estimate of variation within each group of data? Is the variance similar between the groups that are being statistically compared?	Yes	Materials and Methods

Sample definition and in-laboratory replication	Information included in the manuscript?	In which section is the information available? (Reagents and Tools Table, Materials and Methods, Figures, Data Availability Section)
In the figure legends: state number of times the experiment was **replicated** in laboratory.	Yes	Figure Legend
In the figure legends: define whether data describe **technical or biological replicates**.	Yes	Figure Legend

Ethics

Ethics	Information included in the manuscript?	In which section is the information available? (Reagents and Tools Table, Materials and Methods, Figures, Data Availability Section)
Studies involving **human participants**: State details of **authority granting ethics approval** (IRB or equivalent committee(s), provide reference number for approval.	Not Applicable	
Studies involving **human participants**: Include a statement confirming that **informed consent** was obtained from all subjects and that the experiments conformed to the principles set out in the WMA Declaration of Helsinki and the Department of Health and Human Services Belmont Report.	Not Applicable	
Studies involving **human participants: For** publication of **patient photos**, include a statement confirming that consent to publish was obtained.	Not Applicable	
Studies involving experimental **animals**: State details of **authority granting ethics approval** (IRB or equivalent committee(s), provide reference number for approval. Include a statement of compliance with ethical regulations.	Yes	Materials and Methods
Studies involving **specimen and field samples:** State if relevant **permits** obtained, provide details of authority approving study; if none were required, explain why.	Not Applicable	

Dual Use Research of Concern (DURC)	Information included in the manuscript?	In which section is the information available? (Reagents and Tools Table, Materials and Methods, Figures, Data Availability Section)
Could your study fall under dual use research restrictions? Please check biosecurity documents and list of **select agents and toxins** (CDC): https://www.selectagents.gov/sat/list.htm	Not Applicable	
If you used a select agent, is the security level of the lab appropriate and reported in the manuscript?	Not Applicable	
If a study is subject to dual use research of concern regulations, is the name of the **authority granting approval and reference number** for the regulatory approval provided in the manuscript?	Not Applicable	

Reporting

The MDAR framework recommends adoption of discipline-specific guidelines, established and endorsed through community initiatives. Journals have their own policy about requiring specific guidelines and recommendations to complement MDAR.

Adherence to community standards	Information included in the manuscript?	In which section is the information available? (Reagents and Tools Table, Materials and Methods, Figures, Data Availability Section)
State if relevant guidelines or checklists (e.g., **ICMJE, MIBBI, ARRIVE, PRISMA**) have been followed or provided.	Not Applicable	
For **tumor marker prognostic studies,** we recommend that you follow the **REMARK** reporting guidelines (see link list at top right). See author guidelines, under 'Reporting Guidelines'. Please confirm you have followed these guidelines.	Not Applicable	
For **phase II and III randomized controlled trials**, please refer to the **CONSORT** flow diagram (see link list at top right) and submit the CONSORT checklist (see link list at top right) with your submission. See author guidelines, under 'Reporting Guidelines'. Please confirm you have submitted this list.	Not Applicable	

Data Availability

Data availability	Information included in the manuscript?	In which section is the information available? (Reagents and Tools Table, Materials and Methods, Figures, Data Availability Section)
Have **primary datasets** been deposited according to the journal's guidelines (see 'Data Deposition' section) and the respective accession numbers provided in the Data Availability Section?	Yes	Reagents and Tools Table
Were **human clinical and genomic datasets** deposited in a public access-controlled repository in accordance to ethical obligations to the patients and to the applicable consent agreement?	Not Applicable	
Are **computational models** that are central and integral to a study available without restrictions in a machine-readable form? Were the relevant accession numbers or links provided?	Not Applicable	
If publicly available data were reused, provide the respective **data citations in the reference list**.	Yes	Materials and Methods
